# Cyclo[2]carbazole[2]pyrrole: a preorganized calix[4]pyrrole analogue†

**DOI:** 10.1039/d2sc06376j

**Published:** 2022-12-26

**Authors:** Areum Lee, Ju Ho Yang, Ju Hyun Oh, Benjamin P. Hay, Kyounghoon Lee, Vincent M. Lynch, Jonathan L. Sessler, Sung Kuk Kim

**Affiliations:** a Department of Chemistry and Research Institute of Natural Science, Gyeongsang National University Jinju-si Gyeongsangnam-do 52828 Korea sungkukkim@gnu.ac.kr; b Supramolecular Design Institute Oak Ridge Tennessee 37830 USA; c Department of Chemistry Education and Research Institute of Natural Science, Gyeongsang National University Jinju 52828 Korea; d Department of Chemistry, The University of Texas at Austin 105 E. 24th, Street-Stop A5300 Austin Texas 78712-1224 USA sessler@cm.utexas.edu

## Abstract

A cyclo[2]carbazole[2]pyrrole (2) consisting of two carbazoles and two pyrroles has been synthesized by directly linking the carbazole 1- and 8-carbon atoms to the pyrrole α-carbon atoms. Macrocycle 2 is an extensively conjugated 16-membered macrocyclic ring that is fixed in a pseudo-1,3-alternate conformation. This provides a preorganized anion binding site consisting of two pyrrole subunits. ^1^H NMR spectroscopic analysis revealed that only the two diagonally opposed pyrrole NH protons, as opposed to the carbazole protons, take part in anion binding. Nevertheless, cyclo[2]carbazole[2]pyrrole 2 binds representative anions with higher affinity in CD_2_Cl_2_ than calix[4]pyrrole (1), a well-studied non-conjugated tetrapyrrole macrocycle that binds anions *via* four pyrrolic NH hydrogen bond interactions. On the basis of computational studies, the higher chloride anion affinity of receptor 2 relative to 1 is rationalized in terms of a larger binding energy and a lower host strain energy associated with anion complexation. In the presence of excess fluoride or bicarbonate anions, compound 2 loses two pyrrolic NH protons to produce a stable dianionic macrocycle [2–2H]^2−^ displaying a quenched fluorescence.

## Abstract 2

Less is more: two NH hydrogen bond donors in a preorganized receptor provide greater anion affinity than the four NH moieties present in the classic anion receptor, calix[4]pyrrole.

## Graphical abstract

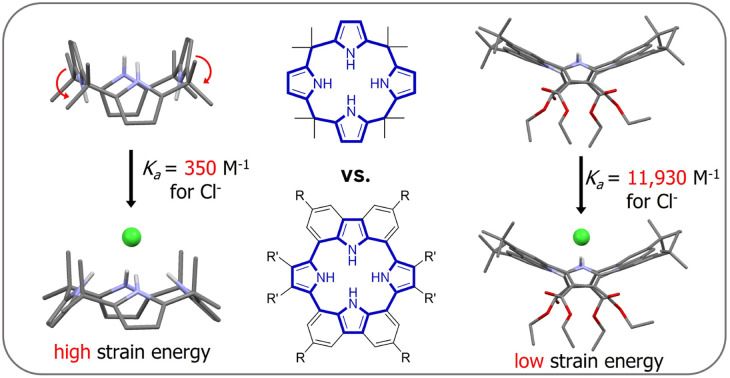

## Introduction

**Fig. 1:**
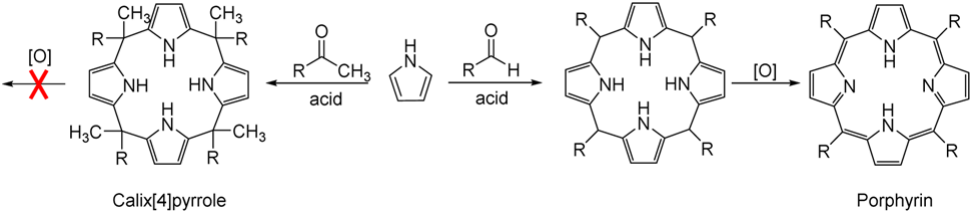
General synthetic schemes for a calix[4]pyrrole and a porphyrin.

Anion recognition by either natural or artificial receptors is involved in myriad biological, chemical, and environmental processes.^1–6^ Therefore, anion receptors capable of binding certain anions with high affinity and selectivity continue to attract interest because of their potential utility in catalysis, anion extraction, and anion sensing, as well as possible treatments for ion transport-related diseases, including cancer.^1–19^ To date, hydrogen bonding donors, such as urea, amide, squaramide, indole, and pyrrole, have been extensively studied as anion binding motifs.^1–19^ In this context, calix[4]pyrrole (1) has attracted attention as an anion receptor because of its facile, one-step synthesis and the relative ease with which it can be modified at its *meso*- and β-pyrrolic positions.^20–26^ Calix[4]pyrrole is a non-conjugated tetrapyrrole macrocycle containing four pyrrole subunits linked *via* four sp^3^ hybridized *meso*-carbons. In contrast to porphyrins (shown generically in Fig. 1), generally synthesized by condensation reactions of pyrrole with aldehydes followed by oxidation reactions, calix[4]pyrroles derived from ketones instead of aldehydes are resistant to oxidation because all four sp^3^ hybridized *meso*-carbon atoms are fully functionalized (Fig. 1).^20–26^ Thus, in contrast to porphyrins, which contain two rather acidic central protons, all four pyrrole subunits in calix[4]pyrroles can act as NH hydrogen bond donors, making them effective for anion recognition.

**Fig. 2:**
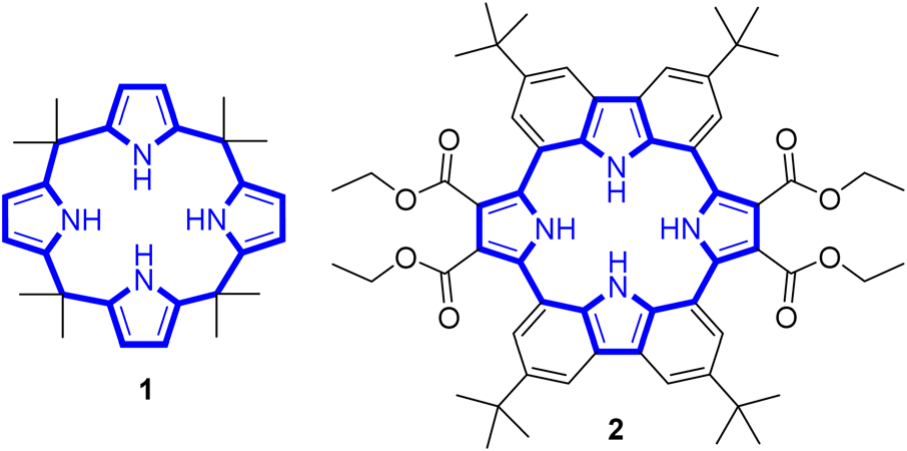
Chemical structures of receptors 1 and 2.

Calix[4]pyrrole adopts four possible limiting conformations, *i.e.*, cone, partial cone, 1,2-alternate, and 1,3-alternate conformations which are interconvertible in solutions.^20–26^ Typically, the 1,3-alternate conformation dominates in the absence of a bound substrate. In contrast, upon interaction with a strongly bound anionic guest, the cone-conformation becomes favoured as the result of forming four N–H⋯anion hydrogen bonds.^27,28^ Calix[4]pyrroles in the cone-conformation also provide π-electron rich cavities that can act as a cation binding site for large charge-diffuse cations such as cesium, thallium, tetraalkylammonium, and imidazolium cations.^26–29^ Here we report the preparation of the cyclo[2]carbazole[2]pyrrole derivative 2 (Fig. 2) and show that it provides a preorganized binding site with two pyrrole-derived NH hydrogen bond donors. As detailed below, this new conjugated macrocycle is a more effective anion receptor for common test anions than calix[4]pyrrole 1 in CH_2_Cl_2_, notwithstanding the difference in available NH recognition sites. Moreover, and in contrast to calix[4]pyrrole, the present cyclo[2]carbazole[2]pyrrole is fluorescent with anion binding serving to reduce the emission intensity. Double deprotonation is seen in the presence of excess fluoride anion with a particularly high level of fluorescence quenching being observed.

## Results and discussion

**Scheme 1:**
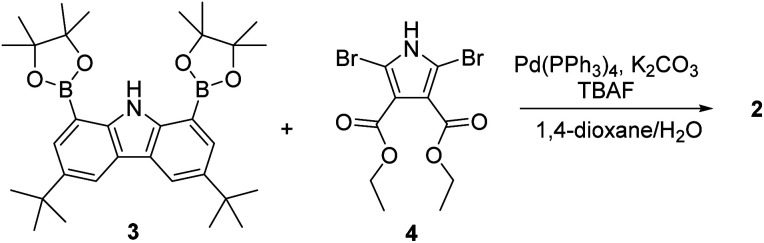
Synthesis of receptor 2.

The synthesis of macrocycle 2 is shown in Scheme 1. The 1,8-carbazolediboronic acid bis(pinacol) ester (3) and 2,5-dibromopyrrole-3,4-diethyl ester (4) were prepared following literature procedures.^30,31^ Under the Suzuki reaction condition using Pd(PPh_3_)_4_ as a catalyst and K_2_CO_3_ as a base in the presence of 1.0 equiv. of tetrabutylammonium fluoride (TBAF) as a template, 3 and 4 were found to undergo 2 : 2 cyclization to give the 2 in 5.2% yield. Compound 2 was characterized by means of ^1^H and ^13^C NMR spectroscopy and high-resolution mass spectrometry, as well as a single crystal X-ray diffraction analysis. Compound 2 was found to be resistant to oxidation when treated with MnO_2_, FeCl_3_ and DDQ (2,3-dichloro-5,6-dicyano-1,4-benzoquinone). This stands in stark contrast to what is seen for related compounds lacking ester groups on the β-pyrrolic positions and presumably reflects the electron-withdrawing ester groups that reduce the electron density within the macrocycle.^32^ The presence of four NH protons in the core in combination with the conjugated structure was expected to limit considerably the mobility of receptor 2 leading to it being locked in the 1,3-alternate conformation. Support for these suppositions came from a combination of structural, spectroscopic, and computational studies (*vide infra*).

**Fig. 3:**
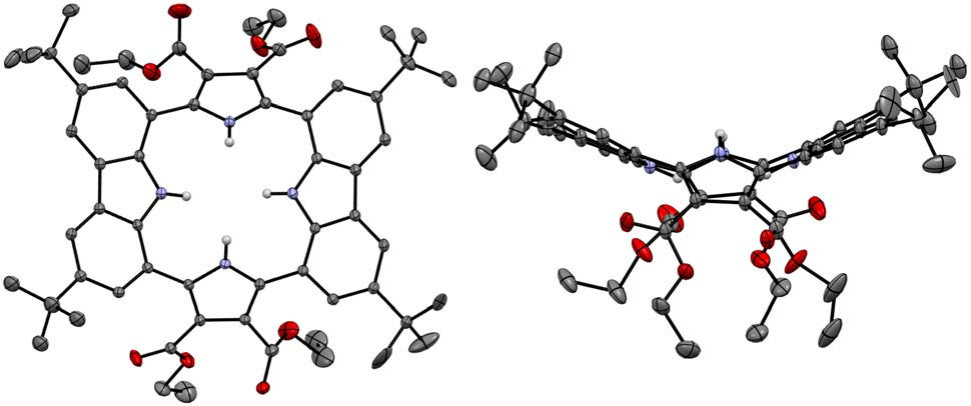
Two different views of the X-ray crystal structure of receptor 2. Most hydrogen atoms and solvent molecules have been removed for clarity. Displacement ellipsoids are scaled to the 30% probability level.

Single crystals of the anion-free receptor 2 suitable for an X-ray diffraction analysis were grown by subjecting a chloroform/methanol solution containing receptor 2 to slow evaporation. The resulting X-ray crystal structure revealed that receptor 2 adopts a pseudo-1,3-alternate conformation in the solid state in which the two pyrrole NH and two carbazole NH moieties are facing in opposite directions. The two pyrrole rings deviate by 41° and 36°, respectively, from the mean plane consisting of the two respective carbazole 1- and 8-carbon atoms, while the two carbazoles are tilted by −24° and −25°, respectively, with respect to the mean plane (Fig. 3). One methanol molecule was found to be hydrogen-bonded to the two opposing pyrrole NH protons.

**Fig. 4:**
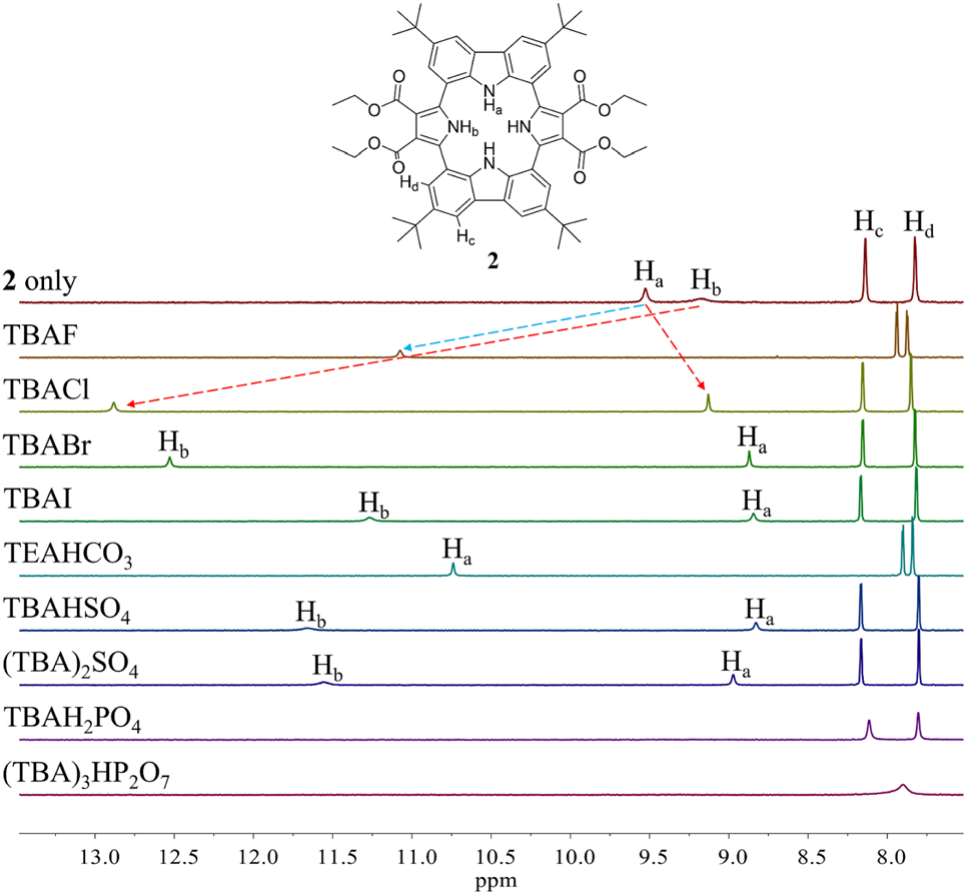
Partial ^1^H NMR spectra of (a) 2 (3 mM) only, (b) 2 + excess TBAF (tetrabutylammonium fluoride), (c) 2 + excess TBACl, (d) 2 + excess TBABr, (e) 2 + excess TBAI, (f) 2 + excess TEAHCO_3_, (g) 2 + excess TBAHSO_4_, (h) 2 + excess (TBA)_2_SO_4_, (i) 2 + excess TBAH_2_PO_4_, and (g) 2 + excess (TBA)_3_HP_2_O_7_ in CD_2_Cl_2_.

Initial evidence that receptor 2 would bind various test anions came from observing the ^1^H NMR spectral changes that take place in the presence of the corresponding tetraalkylammonium salts in CD_2_Cl_2_ (Fig. 4). Receptor 2 in its anion-free form features two broad singlet signals at *δ* = 9.53 ppm and 9.18 ppm corresponding to the carbazole and the pyrrole NH protons, respectively. Upon exposure of receptor 2 to various anions, including F^−^, Cl^−^, Br^−^, I^−^, HSO_4_^−^, SO_4_^2−^, H_2_PO_4_^−^, and HP_2_O_7_^3−^ (as their respective tetrabutylammonium (TBA^+^) salts), as well as HCO_3_^−^ as its tetraethylammonium (TEA^+^) salt, chemical shift changes in the NH proton signals of both the pyrrole and the carbazole subunits are seen in the corresponding ^1^H NMR spectra recorded in CD_2_Cl_2_ (Fig. 4). For most test anions when added in excess, the pyrrole NH proton signal (H_b_) was downfield shifted. *i.e.*, to *δ* = 12.88 ppm (Δ*δ* = 3.7 ppm) for Cl^−^, *δ* = 12.53 ppm (Δ*δ* = 3.35 ppm) for Br^−^, *δ* = 11.27 ppm (Δ*δ* = 2.09 ppm) for I^−^, *δ* = 11.66 ppm (Δ*δ* = 2.48 ppm) for HSO_4_^−^, and *δ* = 11.56 ppm (Δ*δ* = 2.38 ppm) for SO_4_^2−^ (Fig. 4). By contrast, the carbazole NH protons (H_a_) underwent discernible upfield shifts and were found to resonate as a singlet, a singlet signal at *δ* = 9.13 ppm (Δ*δ* = −0.4 ppm) for Cl^−^, *δ* = 8.87 ppm (Δ*δ* = −0.66 ppm) for Br^−^, *δ* = 8.85 ppm (Δ*δ* = −0.68 ppm) for I^−^, *δ* = 8.83 ppm (Δ*δ* = −0.70 ppm) for HSO_4_^−^, and *δ* = 8.97 ppm (Δ*δ* = −0.56 ppm) for SO_4_^2−^ (Fig. 4). This observance led us to suggest that only the two pyrrole NH protons participate in the binding of these anions *via* hydrogen bonding interactions. This stands in contrast to what was seen with calix[4]pyrrole 1, which binds most anions using all four of its potential pyrrole NH hydrogen bond donors. In the presence of excess fluoride and bicarbonate anion salts, the carbazole NH proton signals of 2 were seen to shift to lower field, giving final values of *δ* = 11.07 ppm (Δ*δ* = 1.54 ppm) and *δ* = 10.74 ppm (Δ*δ* = 1.21 ppm) for the fluoride and bicarbonate anions, respectively. The pyrrole NH proton resonance was seen to disappear (Fig. 4).

**Fig. 5:**
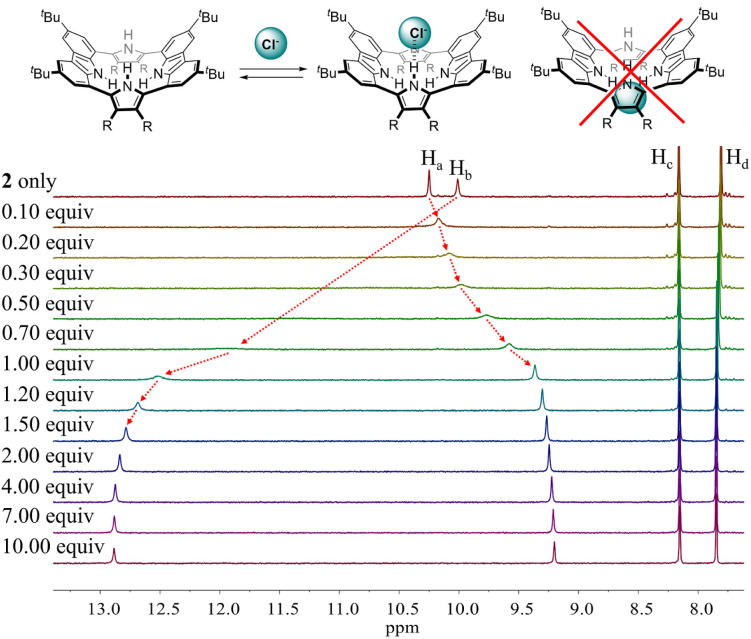
(Top) Putative binding mode for the chloride anion complex of receptor 2. (Bottom) Partial ^1^H NMR spectra recorded during the titration of receptor 2 (3 mM) with tetrabutylammonium chloride (TBACl) in CD_2_Cl_2_.

To evaluate the affinity of receptor 2 for the test anions, we carried out ^1^H NMR spectroscopic titrations in CD_2_Cl_2_. In a first study, when receptor 2 was subjected to titration with the chloride anion, the pyrrole NH proton signal gradually shifted to lower field region while the carbazole NH protons experienced continuous upfield shifts before saturation was achieved upon addition of ≈2 equiv. (Fig. 5). These findings are rationalized by the presumption that only the pyrrole NH protons but not the carbazole NHs bind the chloride anion *via* hydrogen bonds.

**Fig. 6:**
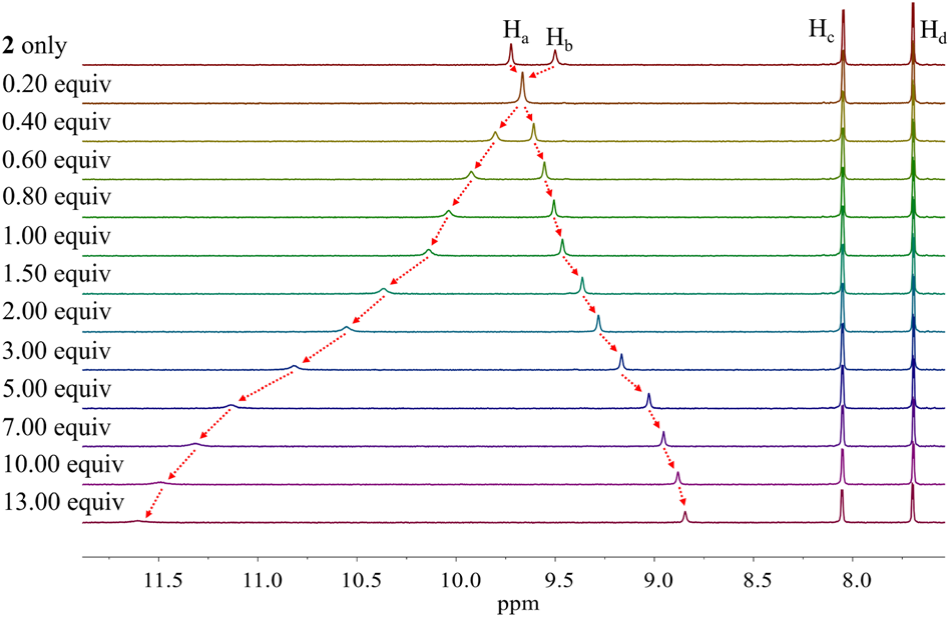
Partial ^1^H NMR spectra recorded during the titration of receptor 2 (3 mM) with tetrabutylammonium iodide (TBAI) in CD_2_Cl_2_.

Similar chemical shift changes were observed when receptor 2 was titrated with other anions such as Br^−^, I^−^, HSO_4_^−^, SO_4_^2−^, H_2_PO_4_^−^, and HP_2_O_7_^3−^ (Fig. 6 and S1–S5†), albeit in the case of H_2_PO_4_^−^, and HP_2_O_7_^3−^, the pyrrole NH proton signal disappears during the course of the titration. This is ascribed to peak broadening resulting from the interaction between these relatively basic anions and the pyrrole NH protons as seen true for numerous anion receptors.^33^ From these titrations, association constants (*K*_a_) for 2 in CD_2_Cl_2_ were calculated to be (1.20 ± 0.18) × 10^4^ M^−1^ for chloride, (6.93 ± 0.12) × 10^2^ M^−1^ for bromide, (1.35 ± 0.08) × 10^2^ M^−1^ for iodide, (3.10 ± 0.18) × 10^2^ M^−1^ for hydrogen sulfate, (5.46 ± 1.03) × 10^2^ M^−1^ for sulfate, and (1.78 ± 0.32) × 10^3^ M^−1^ for dihydrogen phosphate, respectively (Table 1).^34^ Compared with those of calix[4]pyrrole 1 determined in the same solvent system, these values are larger by 34-fold, 69-fold, >14-fold, >31-fold and 18-fold for chloride, bromide, iodide, hydrogen sulfate, and dihydrogen phosphate, respectively (Table 1). The enhanced anion affinity of receptor 2 relative to calix[4]pyrrole is ascribable to its rigid and highly preorganized anion binding motif consisting of two diagonal pyrroles, as well as the relatively high acidity of the pyrrole NHs, and corresponding hydrogen bond donor ability, due to the electron-withdrawing diester groups on the pyrrole β-carbons, and extended conjugation length of the pyrrole subunits to the carbazole moieties.^35^ On the basis of a theoretical analysis (*vide infra*), we believe the former determinant represents the dominant contribution.

**Table 1:** Association constants (*K*_a_, M^−1^) of receptors 1 and 2 for anions as estimated by ^1^H NMR spectroscopic titrations in CD_2_Cl_2_ at room temperature

Anions^a^	1 (*K*_a1_)^b^	2 (*K*_a2_)^c^	*K*_a2_/*K*_a1_
F^−^	(1.72 ± 0.09) × 10^4^	ND	—
Cl^−^	(3.50 ± 0.06) × 10^2^	(1.20 ± 0.18) × 10^4^	34
Br^−^	10 ± 0.5	(6.93 ± 1.29) × 10^2^	69
I^−^	<10	(1.35 ± 0.08) × 10^2^	>14
HSO_4_^−^	<10	(3.10 ± 0.18) × 10^2^	>31
SO_4_^2−^	ND	(5.46 ± 1.03) × 10^2^	—
H_2_PO_4_^−^	97 ± 3	(1.78 ± 0.32) × 10^3^	18

a All anions were used in the form of their respective tetrabutylammonium (TBA^+^) salts.

b From ref. 20; ND, not determined.

c The *K*_a_ values were approximated using BindFit v5.0 available from URL: “https://app.supramolecular.org/bindfit/”.

**Fig. 7:**
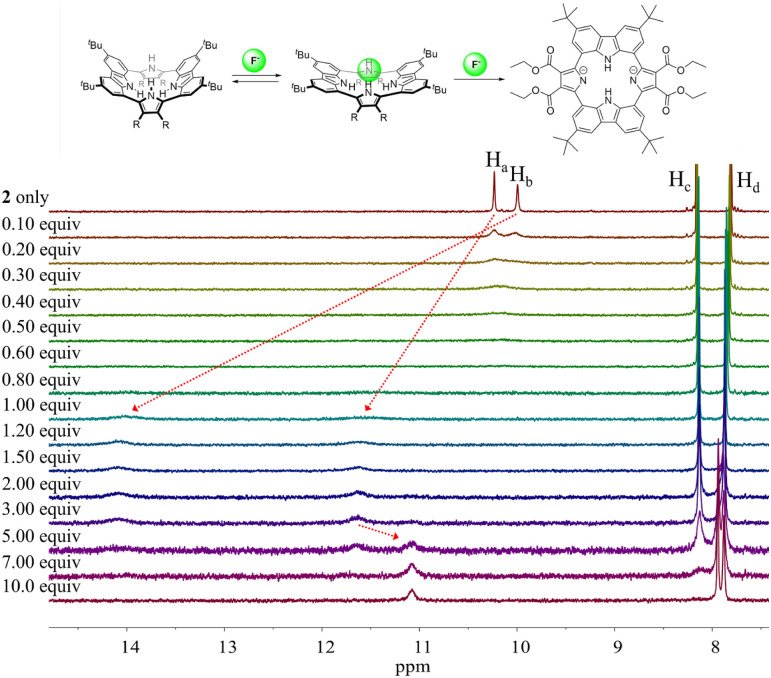
(Top) Proposed interaction modes between receptor 2 and the fluoride anion. (Bottom) Partial ^1^H NMR spectra recorded during the titration of receptor 2 (3 mM) with tetrabutylammonium fluoride (TBAF) in CD_2_Cl_2_.

Different changes in the ^1^H NMR spectra were observed when receptor 2 was titrated with the fluoride or bicarbonate anions in CD_2_Cl_2_ (Fig. 7 and S6†). For example, when receptor 2 was treated with increasing quantities of tetrabutylammonium fluoride (TBAF), the NH proton signals of both the pyrrole and the carbazole subunits were seen to shift to lower field before the chemical shift changes reached a plateau upon the addition of 1.2 fluoride anion equiv. At this juncture the NH protons signals corresponding to the pyrroles and the carbazole resonated at 14.08 ppm (Δ*δ* = 4.09 ppm) and 11.61 ppm (Δ*δ* = 1.38 ppm), respectively. These findings are rationalized in terms of the relatively small fluoride anion binding deep within the receptor cavity forming weak hydrogen bonds to the carbazole NH protons in addition to relatively strong hydrogen bonds to the pyrrole NHs protons (Fig. 7). The saturation achieved upon adding *ca.* 1.2 fluoride anion equiv. is consistent with receptor 2 binding the fluoride anion with a high affinity and with a 1 : 1 stoichiometry (Fig. 7).

Further addition of TBAF beyond 3.0 equiv. gave rise to a new singlet at 11.07 ppm attributable to the carbazole NH protons. A noticeable upfield shift of the carbazole CH signal on C4 (H_c_) was also seen. In addition, the pyrrole NH proton resonance was seen to disappear in the presence of >7.0 equiv. of TBAF. These chemical shift changes are attributed to deprotonation of the pyrrole protons in the presence of excess fluoride anion, which results in an intramolecular hydrogen bond interaction between the carbazole NHs and the deprotonated pyrrolic nitrogen atoms. The noticeable upfield shift of the carbazole CH proton signal (H_d_) is thought to reflect the increased electron density of the macrocycle that results from deprotonation of the pyrrole NHs. Support for the suggestion that deprotonation occurs under these conditions came from an X-ray crystal structural analysis of single crystals of receptor 2 grown in the presence of excess TBAF (*vide infra*). Unfortunately, an association constant corresponding to the interaction of receptor 2 with the fluoride anion could not be approximated from these ^1^H NMR spectral titrations due to the disappearance of the pyrrole and carbazole NH proton signals and the absence of appreciable chemical shift changes for the carbazole CH proton signals before saturation was reached upon the addition of 1.2 equiv. of fluoride (Fig. 7).

More dramatic chemical shift changes were observed when receptor 2 was exposed to fluoride anions in more polar solvents, such as acetone-d_6_, acetonitrile-d_3_, and DMSO-d_6_ (Fig. S7–S9†). For instance, upon titration of receptor 2 with TBAF in acetone-d_6_ and acetonitrile-d_3_, respectively, the carbazole CH proton signal (H_c_) was shifted to higher field with saturations being seen upon the addition of only *ca.* 1.0 anion equiv. in both solvent systems (Fig. S7 and S8†). Evidence of pyrrole NH proton deprotonation was seen in the presence of 2.0 equiv. and 1.4 equiv. of fluoride in acetone-d_6_ and acetonitrile-d_3_, respectively (Fig. S7 and S8†). These findings lead us to suggest that deprotonation of the pyrrole NH protons by the fluoride anion is facilitated in these relatively polar solvents. In contrast, when receptor 2 was titrated with fluoride in DMSO-d_6_, the pyrrole NH proton resonance underwent a gradual downfield shift whereas the carbazole NH proton signal moved upfield continuously, a finding consistent with the notion that only the pyrrole subunits are involved in fluoride binding (Fig. S8†). Deprotonation of the pyrrolic NHs is already observable upon the addition of 0.4 equiv. of fluoride in DMSO-d_6_, *i.e.*, before saturation in the fluoride induced chemical shift changes of 2 are seen (Fig. S9†). In acetone-d_6_, the association constant of receptor 2 for fluoride was calculated to be *K*_a_ = 6.8 × 10^4^ M^−1^ before subsequent pyrrole NH deprotonation occurred (Fig. S7†).

**Fig. 8:**
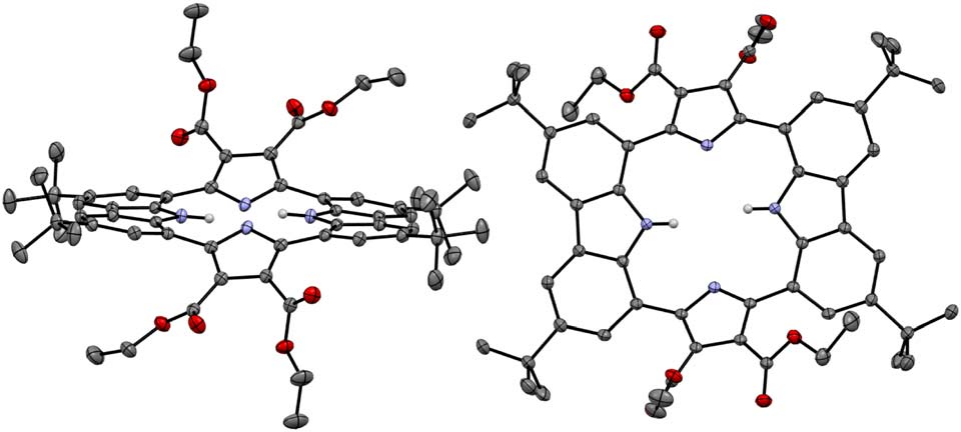
X-ray crystal structure of the deprotonated form of receptor 2 ([2–2H]^2−^). The data crystal was twinned. Most hydrogen atoms, solvent molecules, and two tetrabutylammonium cations positioned above and below the dianionic macrocycle have been removed for clarity. Displacement ellipsoids are scaled to the 50% probability level.

The presumption that the pyrrole subunits of receptor 2 could be deprotonated by the fluoride anion was supported by an X-ray diffraction analysis of single crystals grown by slow evaporation of an acetone/diethyl ether solution of receptor 2 containing excess TBAF. The resulting twinned crystal structure revealed that the two pyrrole NH protons of receptor 2 are lost and the macrocycle exists as a dianion ([2–2H]^2−^) with two TBA^+^ counter cations. This dianionic form is flattened relative to the charge neutral receptor (2). While the two pyrrole rings are tilted by 33° and −33°, respectively, with respect to the mean plane of the macrocycle while the carbazole subunits lie in approximately the same plane as the mean plane (Fig. 8). The carbazole NH protons were found to form hydrogen bonds with the deprotonated pyrrole N atoms at a distance of 2.32 Å for the N–H⋯N^−^ interaction (Fig. 8). The two TBA^+^ cations lie above and below the macrocycle pseudo plane.

**Fig. 9:**
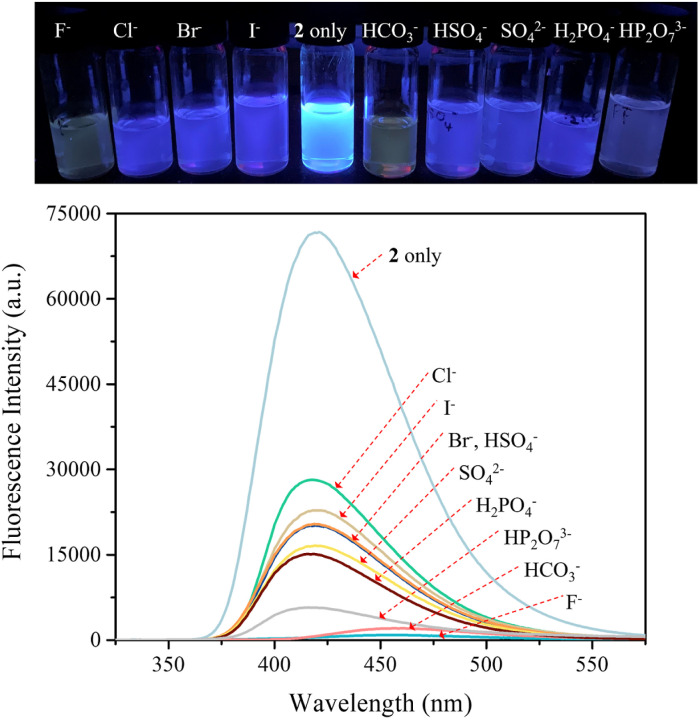
(Top) Photographs showing the fluorescence changes of CH_2_Cl_2_ solutions of receptor 2 (10 μM) in the presence of the indicated anions (as their respective TBA^+^ salts for all anions but the bicarbonate anion, which was used in its TEA^+^ salt form). (Bottom) Corresponding fluorescence spectra of receptor 2 (10 μM) upon excitation at 301 nm.

**Fig. 10:**
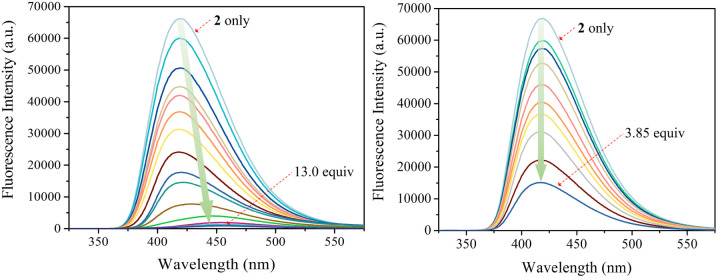
Fluorescence spectra of receptor 2 (10 μM) recorded during titrations with TBAF (left) and TBAH_2_PO_4_ (right), respectively, in CH_2_Cl_2_. The excitation wavelength (*λ*_ex_) was = 301 nm.

The carbazole and pyrrole subunits are formally conjugated in macrocycle 2. This stands in marked contrast to what is true for 1. Therefore, we interrogated the optical properties of 2 using UV/Vis and fluorescence spectroscopies. Receptor 2 at a concentration of 10 μM was found to exhibit absorption peaks at 301 nm and 367 nm and an emission peak at 421 nm in dichloromethane upon excitation at 301 nm (Fig. S10†). Upon treatment of receptor 2 with the test anions, the fluorescence emission was quenched in the case of most anions (Fig. 9). These fluorescence changes are ascribable to PET (photoinduced electron transfer) between the receptor fluorophore and the bound anion.^36^ The fluoride and bicarbonate anions gave rise to particularly large levels of fluorescence quenching. A shift in the emission peaks to longer wavelengths was also seen (Fig. 9). The absorption peaks of receptor 2 were also found to undergo bathochromic shifts in the presence of most test anions (Fig. S11†). Association constants for the anions in question were determined from quantitative fluorescence spectral titrations using a 10 μM solution of receptor 2 (Fig. 10 and S12–S18†). The resulting association constants (*K*_a_) were found to fall in the range of 6.8 × 10^4^ M^−1^ to 4.0 × 10^5^ M^−1^ with errors under 10% (Table 2).^34^ These *K*_a_ values are significantly larger than those determined by ^1^H NMR spectral titrations (*cf.*Table 1). This finding is attributed to weakened ion pairing between the anion under study and the tetrabutylammonium countercation at relatively lower concentrations.^37^ In the other words, at the higher concentrations associated with the ^1^H NMR spectroscopic titrations the present receptors bind anions in stronger competition with ion pairing of the starting salt form of the anion. This results in lower apparent association constants.

**Table 2:** Association constants (*K*_a_, M^−1^) of receptor 2 (10 μM) for anions as determined by fluorescence spectral titrations in CD_2_Cl_2_ at room temperature

Anions	*K*_a_ c (M^−1^)
F^−^a	(1.06 ± 0.05) × 10^5^
Cl^−^a	(3.11 ± 0.20) × 10^5^
Br^−^a	(3.31 ± 0.17) × 10^5^
I^−^a	(2.59 ± 0.24) × 10^5^
HCO_3_^−^b	(1.24 ± 0.13) × 10^5^
HSO_4_^−^a	(2.73 ± 0.18) × 10^5^
SO_4_^2−^a	(4.04 ± 0.42) × 10^5^
H_2_PO_4_^−^a	(6.81 ± 0.32) × 10^4^
HP_2_O_7_^3−^a	(2.11 ± 0.14) × 10^5^

a The anion was used in the form of the corresponding tetrabutylammonium (TBA^+^) salt.

b The anion was used in the form of the corresponding tetraethylammonium (TEA^+^) salt.

c The *K*_a_ values were calculated using BindFit v5.0 available from URL: “https://app.supramolecular.org/bindfit/”.

The presumed binding modes of receptor 2 for fluoride, chloride and hydrogen sulfate in solution were supported by molecular mechanics computations performed using the Merck Force Field 94 (MMFF94) model *in vacuo*.^38,39^ This modeling approach was chosen because MMFF94 is one of the most accurate force fields available^39,40^ and it has proved successful at correlating observed anion affinities in solution with the predicted gas phase structures for numerous other hydrogen bonding anion receptors.^41^ These computations provided the most stable calculated forms of the ion-free receptor 2 and its fluoride, chloride and hydrogen sulfate complexes, respectively, as well as their lowest energy values.^38,39^ The binding energy (Δ*E*) between receptor 2 and the anions, defined as Δ*E* = *E*_(receptor–anion complex)_ − *E*_(receptor)_ − *E*_(anion)_, was computed using the energies for the lowest energy forms of each species. The inherent energies of receptor 2, fluoride, chloride, and hydrogen sulfate were calculated to be 143 kcal mol^−1^, 0.0 kcal mol^−1^, 0.0 kcal mol^−1^ and −100.0 kcal mol^−1^, respectively. Receptor 2 was found to maintain its approximate 1,3-alternate conformation for the main receptor cavity in all simulations. Multiple conformers, arising from rotations involving *t*-butyl and ethyl ester groups, were considered.

**Fig. 11:**
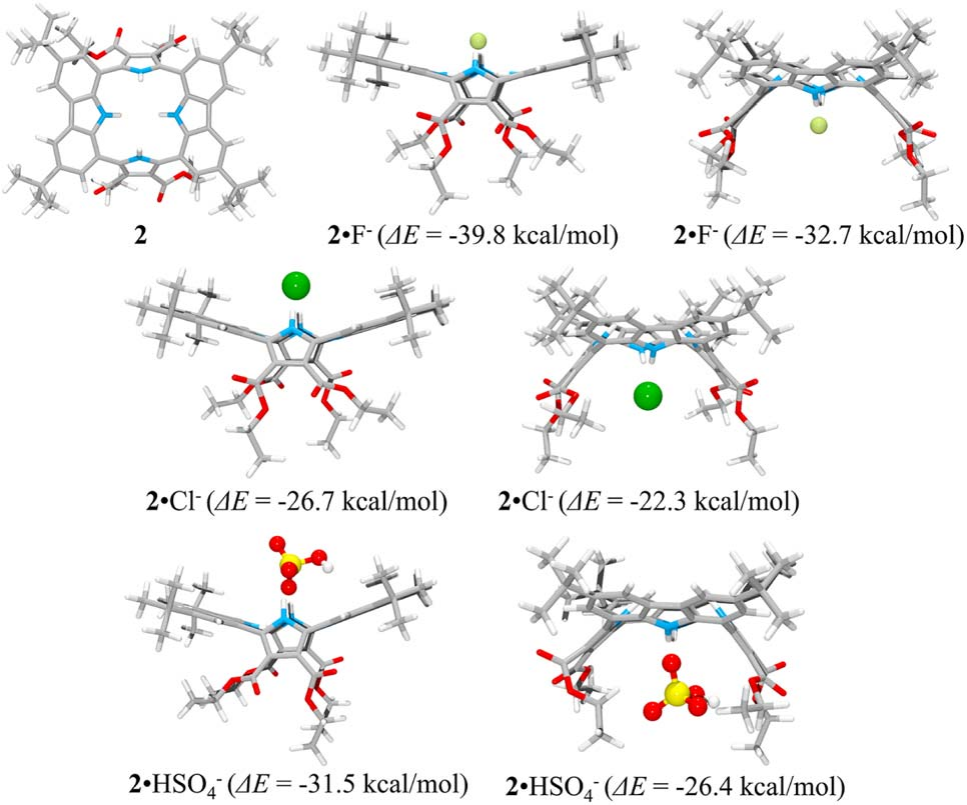
Lowest energy structures of receptor 2 and its two possible limiting complexes with fluoride, chloride, and hydrogen sulfate, respectively, as computed in the gas phase and the corresponding calculated binding energies (Δ*E*) for the interaction of receptor 2 with the anions in question.

Consistent with what was seen in the solid-state X-ray crystal structure of the ion-free form, receptor 2 is characterized by two distinct sides, each of which provides two potential NH binding sites, namely the two pyrrole NH hydrogen bond donors or the two carbazole NH donors, respectively. When an anion is added, the anion remains on the same side of the host during each simulation. In other words, the anion does not migrate through the centre of the receptor cavity; nor does it crawl around the edge of the receptor cavity. The binding energies of receptor 2 for fluoride and chloride were computed to be Δ*E* = −39.8 kcal mol^−1^ and −26.7 kcal mol^−1^, respectively, for binding *via* two pyrroles and Δ*E* = −32.7 kcal mol^−1^ and −22.3 kcal mol^−1^, respectively, for binding *via* two carbazoles, respectively (Fig. 11). The fluoride complex of receptor 2 was calculated to possess a flatter conformation than the corresponding chloride complex, leading us to suggest that an additional interaction between the fluoride anion and the carbazole NHs could be involved in anion binding as inferred from the solution phase studies described above. In the case of the hydrogen sulfate anion, receptor 2 displays a preference for binding *via* its two pyrrole subunits (Δ*E* = −31.5 kcal mol^−1^), rather than its two carbazole subunits (Δ*E* = −26.4 kcal mol^−1^) (Fig. 11). These calculation-based conclusions are in good agreement with those drawn from the solution phase studies and lead us to propose that the pyrrole donor side yields a more stable gas phase anion complex than does the carbazole face and that this holds for fluoride (by 7.1 kcal mol^−1^), chloride (by 4.4 kcal mol^−1^) and hydrogen sulfate (by 5.1 kcal mol^−1^), respectively.

**Fig. 12:**
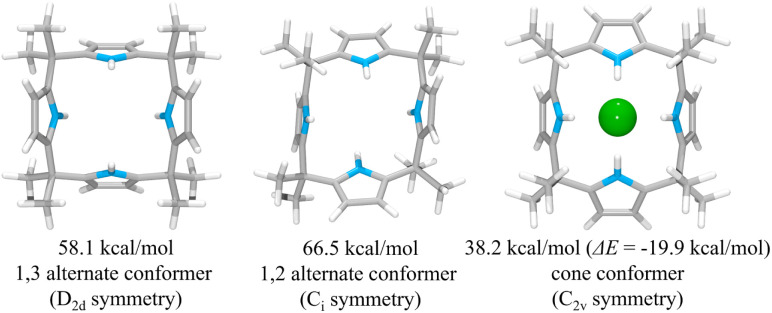
Two lowest energy structures (left and middle) of receptor 1 and the corresponding computed energies. The optimized structure of the complex 1·Cl^−^ (right) and its corresponding computed energy and anion binding energy.

To rationalize the higher anion affinity of receptor 2 compared to calix[4]pyrrole 1, we also calculated the binding energy (Δ*E*) of calix[4]pyrrole 1 for the chloride anion using the MMFF94 model *in vacuo* and compared it with that for receptor 2.^38,39^ For calix[4]pyrrole 1 in its ion-free form, only two stable conformers, *i.e.*, the 1,3-alternate conformer with *D*_2d_ symmetry and the 1,2-alternate conformer with *C*_i_ symmetry, were calculated as being appreciably stable in the gas phase (Fig. 12). The intrinsic energies of the 1,3-alternate conformer and the 1,2-alternate conformer were computed to be 58.1 kcal mol^−1^ and 66.5 kcal mol^−1^, respectively, meaning that in the gas phase the 1,3-alternate conformer is more stable than the 1,2-alternate conformer by 8.4 kcal mol^−1^. This result is consistent with the optimized geometry of calix[4]pyrrole 1 obtained by density functional theory calculations using the BLYP/3-21G and BLYP/6-31G* methods.^42^ In contrast, and in accord with previous studies, the optimized structure of the chloride complex of calix[4]pyrrole 1 was found to have an energy of 38.2 kcal mol^−1^ with the calix[4]pyrrole adopting the cone conformation with *C*_2v_ symmetry (Fig. 12).^42,43^ The binding energy of 1 for the chloride anion was calculated to be −19.9 kcal mol^−1^ which is smaller than that of 2 (Δ*E* = −26.7 kcal mol^−1^) by 6.8 kcal mol^−1^ (Fig. 12). These computation results provide support for the conclusion drawn from the experimental studies, namely that receptor 2 binds the chloride anion more effectively than calix[4]pyrrole 1.

This suggestion was further supported by strain energy analyses of receptors 1 and 2.^44^ According to a method developed by Hay, *et al.*, it is possible to relate the calculated molecular mechanics host strain energies to the degree of complementarity and the degree of preorganization.^45^ According to this protocol, the host–guest complex is first optimized. The guest is then removed to yield the ‘bound’ form of the host. A single point energy calculation yields the energy of this form. The ‘bound’ form of the host is optimized to yield the ‘binding’ form. The difference in energy of the ‘binding’ form and the ‘bound’ form, Δ*U*_comp_, is a measure of how well the ‘binding’ form of the host complements the guest. The lower the energy difference, the higher the degree of complementarity. If the energy difference is 0, then the host ‘binding’ form perfectly complements the guest. The other part of this two-part analysis accounts for the cost of conformational change associated with substrate binding. The host is subject to an energetic conformer search to find the global minimum, which by definition is the ‘preferred’ form. The difference in energy between the ‘preferred’ form and the ‘binding’ form, Δ*U*_conf_, is an energetic measure of preorganization. The lower the energy difference, the higher the degree of preorganization. By combining the two energy components (or simply taking the energy difference between the ‘bound’ form and the ‘preferred’ form), one obtains, Δ*U*_reorg_, the total host strain energy increase on going from the preferred form to the bound form. This value provides a measure of how well the host is structurally organized for binding the guest.

**Fig. 13:**
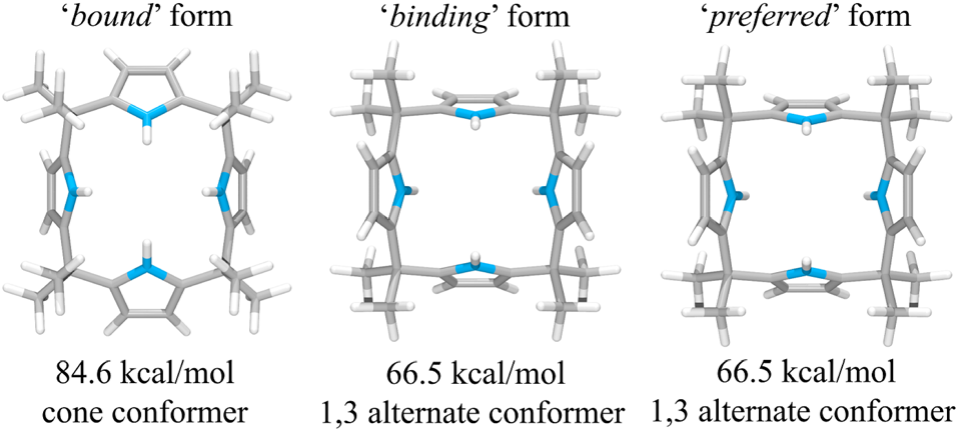
Three structural states of receptor 1 used to define its strain energies and the corresponding computed energy values.

**Fig. 14:**
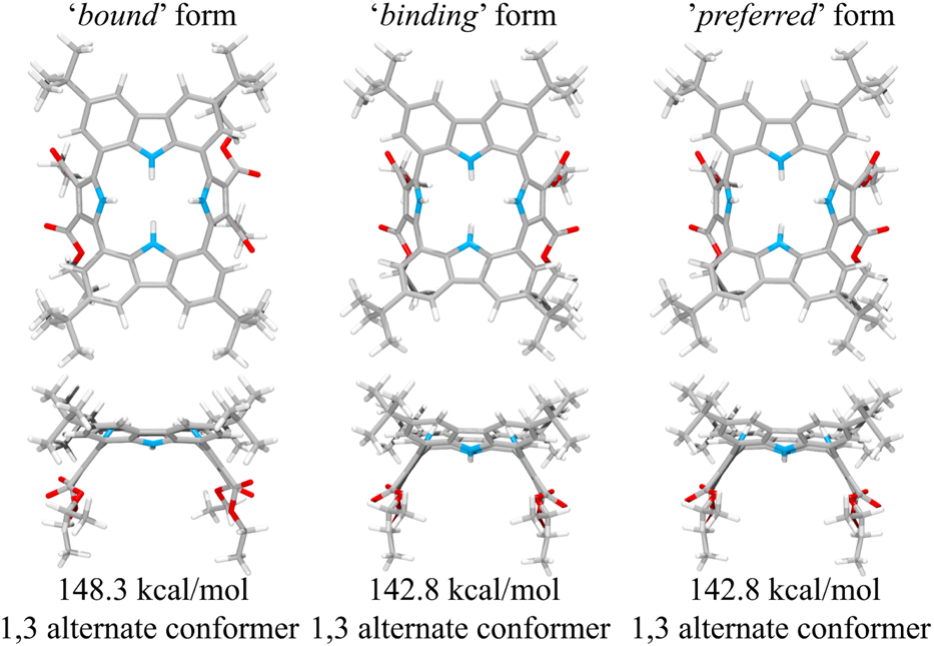
Three structural states of receptor 2 used to define its strain energies and the corresponding computed energy values.

For calix[4]pyrrole 1, the binding conformer is the one obtained when the bound form of the host is optimized (Fig. 13). In this case, optimization of the bound cone form yields the 1,3-alternate binding form. Since the binding conformer is identical to the preferred ion-free form, host 1 is formally preorganized according to this methodology.^45^ However, a strain energy analysis reveals that receptor 2 is significantly more complementary with Δ*U*_comp_ = 5.5 kcal mol^−1^ than calix[4]pyrrole 1 with Δ*U*_comp_ = 18.1 kcal mol^−1^ (Fig. 13 and 14). In other words, chloride complexation by calix[4]pyrrole 1 entails the development of 12.6 kcal mol^−1^ more host strain than complexation by receptor 2. The significantly large host strain associated with calix[4]pyrrole 1 binding the chloride anion relative to receptor 2 provides an explanation why the two NH hydrogen bond donors of host 2 give a stronger chloride complex than the four NH protons of calix[4]pyrrole 1. However, we appreciate that this analysis does not take into account electronic effects. It is well known that adding synthetically electron withdrawing groups to the calix[4]pyrrole framework can enhance the anion affinity. For instance, the octafluoro analogue of 1 displays a chloride anion affinity in acetonitrile-d_3_ (containing 0.5% D_2_O v/v) that is enhanced by roughly a factor of 2.^46^ We postulate that the electronic contribution of the four esters in 2 relative to 1 is likely less than that provided by eight fluorine atoms and that preorganization effects account for the bulk of the *ca.* 34-fold improvement in chloride affinity seen on moving from 1 to 2.

## Conclusions

A conjugated macrocyclic compound 2 having four central NH protons as found in calix[4]pyrrole has been synthesized by means of a 2 : 2 cyclization reaction involving the Suzuki coupling of 1,8-carbazolediboronic acid bis(pinacol) ester with 2,5-dibromopyrrole-3,4-diethyl ester. ^1^H NMR spectral studies and a single crystal X-ray structural analysis in conjunction with molecular mechanics calculations carried out *in vacuo* revealed that receptor 2 adopts a 1,3-alternate conformation in which the two alternating pyrrole and carbazole subunits face in opposite directions. This provides a rigid and highly preorganized anion binding motif with two faces and two potential sets of NH hydrogen bond donors. On the basis of ^1^H NMR spectral analyses, receptor 2 was found to bind most test anions using only the two diagonal opposing pyrrole NH protons but not the carbazole NH protons. Nevertheless, higher anion affinities for 2 relative to calix[4]pyrrole 1 were seen in the same solvent system (CH_2_Cl_2_ for most studies), even though the latter well-studied receptor typically allows for four pyrrole NH–anion interactions. *In vacuo* molecular dynamic computations involving the chloride anion complex provided support for the conclusion that anions energetically favor binding to the pyrrole donor side of receptor 2 over binding to the carbazole donor side. In contrast to most test anions considered in this study, the small basic fluoride anion binds relatively deep within the receptor cavity with the resulting complex being stabilized by hydrogen bonds to both the pyrrole and carbazole NH protons. When treated with an excess of TBAF, receptor 2 loses two protons from its pyrrole subunits giving rise to a stable dianionic macrocyclic salt [2–2H]^2−^·(TBA^+^)_2_. Molecular dynamics computations using the MMFF94 model revealed that the high affinity of receptor 2 for the chloride anion (and by inference other anions) relative to calix[4]pyrrole 1 can be rationalized largely in terms of a larger stabilization energy associated with anion binding and a correspondingly smaller host strain energy associated with complex formation.

## Data availability

The datasets supporting this article have been uploaded as part of the ESI.†

## Author contributions

J. L. S. and S. K. K. conceived and supervised the project. A. L. and J. H. O. synthesized the compounds. A. L. performed ^1^H NMR, UV/Vis, and fluorescence spectroscopic studies. J. H. Y., K. L. and V. M. L. carried out the X-ray diffraction analyses. B. P. H. carried out molecular mechanics computations. J. L. S. and S.·K. K. wrote the manuscript. All authors contributed to the editing of the manuscript.

## Conflicts of interest

There are no conflicts to declare.

## Supplementary Material

SC-014-D2SC06376J-s001

SC-014-D2SC06376J-s002

## References

[cit1] Sessler J. L. , Gale P. A. and Cho W.-S., Anion Receptor Chemistry, ed. J. F. Stoddart, Royal Society of Chemistry, Cambridge, 2006

[cit2] Lehn J. M. , Supramolecular Chemistry, VCH Press, Weinheim, Germany, 1995

[cit3] Desvergne J. P. and Czarnik A. W., Chemosensors of Ion and Molecular Recognition; NATO ASI Series C, Kluwer, Dordrecht, The Netherlands, 1997

[cit4] Bianchi A. , Bowman-James K. and García-España E., Supramolecular Chemistry of Anions, Willey, New York, 1997

[cit5] Schmidtchen F. P. (1988). Nachr. Chem., Tech. Lab..

[cit6] Bowman-James K. , Bianchi A. and García-España E., Anion Coordination Chemistry, Wiley-VCH, Weinheim, 2011

[cit7] Beer P. D., Gale P. A. (2001). Angew. Chem., Int. Ed..

[cit8] Martínez-Máñez R., Sancenán F. (2003). Chem. Rev..

[cit9] Gale P. A., Busschaert N., Haynes C. J. E., Karagiannidis L. E., Kirby I. L. (2014). Chem. Soc. Rev..

[cit10] Wenzel M., Hiscock J. R., Gale P. A. (2012). Anion receptor chemistry: highlights from 2010. Chem. Soc. Rev..

[cit11] Gale P. A. (2010). Anion receptor chemistry: highlights from 2008 and 2009. Chem. Soc. Rev..

[cit12] Caltagirone C., Gale P. A. (2009). Chem. Soc. Rev..

[cit13] Gale P. A., Caltagirone G. (2015). Chem. Soc. Rev..

[cit14] Kang S. O., Begum R. A., Bowman-James K. (2006). Angew. Chem., Int. Ed..

[cit15] Kang S. O., Llinares J. M., Day V. W., Bowman-James K. (2010). Chem. Soc. Rev..

[cit16] Chen L., Berry S. N., Wu X., Howe E. N. W., Gale P. A. (2020). Chem.

[cit17] Macreadie L. K., Gilchrist A. M., McNaughton D. A., Ryder W. G., Fares M., Gale P. A. (2022). Chem.

[cit18] Davis J. T., Gale P. A., Okunola O. A., Prados P., Iglesias-Sánchez J. C., Tomás T., Quesada R. (2009). Nat. Chem..

[cit19] Ko S.-K., Kim S. K., Share A., Lynch V. M., Park J., Namkung W., Rossom W. V., Busschaert N., Gale P. A., Sessler J. L., Shin I. (2014). Nat. Chem..

[cit20] Gale P. A., Sessler J. L., Král V., Lynch V. (1996). J. Am. Chem. Soc..

[cit21] Allen W. E., Gale P. A., Brown C. T., Lynch V. M., Sessler J. L. (1996). J. Am. Chem. Soc..

[cit22] Gale P. A., Sessler J. L., Král V. (1998). Chem. Commun..

[cit23] Lee C.-H., Miyaji H., Yoon D.-W., Sessler J. L. (2008). Chem. Commun..

[cit24] Saha I., Lee J. T., Lee C.-H. (2015). Eur. J. Org. Chem..

[cit25] Peng S., He Q., Vargas-Zúñiga G. I., Qin L., Hwang I., Kim S. K., Heo N. J., Lee C.-H., Dutta R., Sessler J. L. (2020). Chem. Soc. Rev..

[cit26] Sessler J. L., Gross D. E., Cho W.-S., Lynch V. M., Schmidtchen F. P., Bates G. W., Light M. E., Gale P. A. (2006). J. Am. Chem. Soc..

[cit27] Custelcean R., Delmau L. H., Moyer B. A., Sessler J. L., Cho W.-S., Gross D., Bates G. W., Brooks S. J., Light M. E., Gale P. A. (2005). Angew. Chem., Int. Ed..

[cit28] Kim S. K., Sessler J. L. (2014). Acc. Chem. Res..

[cit29] Zhai H., Xiong S., Peng S., Sheng W., Xu G., Sessler J. L., He Q. (2021). Org. Lett..

[cit30] Arnold L., Baumgarten M., Müllen K. (2012). Chem. Commun..

[cit31] Brewster J. T., Zafar H., McVeigh M., Wight C. D., Anguera G., Steinbrück A., Lynch V. M., Sessler J. L. (2018). J. Org. Chem..

[cit32] Arnold L., Baumgarten M., Müllen K. (2012). Chem. Commun..

[cit33] He Q., Kelliher M., Bähring S., Lynch V. M., Sessler J. L. (2017). J. Am. Chem. Soc..

[cit34] Association constants (*K*_a_) were evaluated using BindFit v5.0, available from https://app.supramolecular.org/bindfit/

[cit35] Anzenbacher Jr P., Try A. C., Miyaji H., Jursíková K., Lynch V. M., Marquez M., Sessler J. L. (2000). J. Am. Chem. Soc..

[cit36] Martínez-Máñez R., Sancenón F. (2003). Chem. Rev..

[cit37] Piątek P., Lynch V. M., Sessler J. L. (2004). J. Am. Chem. Soc..

[cit38] Conformational searches performed with PCModel, version 9.3, Serena Software, Bloomington, IN, 2012

[cit39] Halgren T. A. (1996). Merck molecular force field. I. Basis, form, scope, parameterization, and performance of MMFF94. J. Comput. Chem..

[cit40] Gundertofte K., Liljefors T., Norrby P. O. (1996). J. Comput. Chem..

[cit41] Hay B. P. (2010). Chem. Soc. Rev..

[cit42] Wu Y.-D., Wang D.-F., Sessler J. L. (2001). J. Org. Chem..

[cit43] Blas J. R., Márques M., Sessler J. L., Luque F. J., Orozco M. (2002). J. Am. Chem. Soc..

[cit44] Parks F. C., Sheetz E. G., Stutsman S. R., Lutolli A., Debnath S., Paghavachari K., Flood A. H. (2022). J. Am. Chem. Soc..

[cit45] Hay B. P., Zhang D., Rustad J. (1996). Inorg. Chem..

[cit46] Anzenbacher Jr P., Try A. C., Miyaji H., Jurisíková K., Lynch V. M., Marquez M., Sessler J. L. (2000). J. Am. Chem. Soc..

