# Supplementary material for: Cyclo[2]carbazole[2]pyrrole: a preorganized calix[4]pyrrole analogue
Source: Chem Sci. 2022 Dec 26;14(5):1218–26. doi: 10.1039/d2sc06376j (PMC9891360; doi:10.1039/d2sc06376j)
Supplement: SC-014-D2SC06376J-s001 [file SC-014-D2SC06376J-s001.pdf]

Electronic Supplementary Material (ESI) for Chemical Science.
This journal is © The Royal Society of Chemistry 2023

Supporting Information for:

Cyclo[2]carbazole[2]pyrrole: A Preorganized Calix[4]pyrrole Analogue†

Areum Lee,[a] Ju Ho Yang,[a] Ju Hyun Oh,[a] Benjamin P. Hay,[b] Kyounghoon Lee,[c] Vincent M. Lynch,[d] Jonathan L. Sessler,[*,d] and Sung Kuk Kim[*,a]

[a]Department of Chemistry and Research Institute of Natural Science, Gyeongsang National University, Jinju-si, Gyeongsangnam-do 52828, Korea, [b]Supramolecular Design Institute, Oak Ridge, Tennessee 37830 USA, and [c]Department of Chemistry Education and Research Institute of Natural Science, Gyeongsang National University, Jinju, 52828, Korea, and [d]Department of Chemistry, The University of Texas at Austin, 105 E. 24th, Street-Stop A5300, Austin, Texas 78712-1224, USA

1. General experimental and synthetic details S2 – S3

2. ^{1}H NMR spectral studies for anion binding S4 – S12

3. UV-vis and fluorescence spectral data S13 – S21

4. Single crystal X-ray diffraction data S22 – S27

5. NMR and HRMS spectra S28 – S31

6. References S32

7. Molecular Mechanics Computations data S33 – S100

1. General experimental and synthetic details

Solvents and reagents used for the synthetic work were purchased from Aldrich, TCI, or Alfa Aesar and used without further purification. Compounds **3** and **4** were prepared as reported previously.[1,2] NMR spectra were recorded on a Bruker Advance-300 MHz instrument. The NMR spectra were referenced to residual solvent peaks and the spectroscopic solvents were purchased from either Cambridge Isotope Laboratories or Aldrich. Fast atom bombardment (FAB) mass spectra (MS) were recorded on a JMS-700 (JEOL) spectrometer. TLC analyses were carried out using Sorbent Technologies silica gel (200 mm) sheets. Column chromatography was performed on Sorbent silica gel 60 (40–63 mm). The X-ray crystallographic analysis was carried out on a Rigaku Oxford Diffraction HyPix6000E Synergy diffractometer using a μ-focus Cu Kα radiation source (λ = 1.5418 Å) with collimating mirror monochromators. For computations, PCModel[3] was used to locate the lowest energy conformation for the free ligand and various ligand anion complexes. This was done by sampling structures taken from 50 ps molecular dynamics runs using the Merck Force Field 94 model[4] *in vacuo*. The energies for the lowest energy forms of each species were used to compute interaction energies:

$$\Delta E = E_{(complex)} - E_{(host)} - E_{(guest)}$$

Compound 2

3,4-Diethyl 2,5-dibromo-1H-pyrrole-3,4-dicarboxylate (**4**) (500 mg, 1.35 mmol), 3,6-di-tert-butyl-1,8-bis(4,4,5,5-tetramethyl-1,3,2-dioxaborolan-2-yl)-9H-carbazole (**3**) (720 mg, 1.35 mmol), $Pd(PPh_3)_4$ (156 mg, 0.135 mmol), K_2CO_3 (excess), and tetrabutylammonium fluoride (TBAF, 210 mg, 0.68 mmol) were dissolved in a mixture of dioxane and water (55ml, 50:5). The reaction mixture was heated to 100 °C and stirred overnight. After the reaction was deemed complete, the volatile solvents were removed *in vacuo*. To the resulting crude product, dichloromethane and water were added. The organic layer was separated off and washed with water three times. The resulting organic layer was dried over anhydrous $MgSO_4$ and evaporated *in vacuo* to give a brownish solid. Column chromatography over silica gel (eluent: ethyl acetate/hexanes = 1/4), followed by recrystallization from dichloromethane and methanol, gave compound **1** (68 mg, 5.16% yield) as a white solid.; ^{1}H NMR (300 MHz, chloroform-*d*) δ 10.15 (s, 2H), 9.51 (s, 2H), 8.08 (d, J = 1.8 Hz, 4H), 7.76 (d, J = 1.8 Hz, 4H), 4.27 (q, J = 7.2 Hz, 8H), 1.46 (s, 36H), 1.27 (t, J = 7.1 Hz, 12H). ^{13}C NMR (75 MHz, chloroform-*d*) δ 165.2, 142.9, 137.1, 132.5, 126.2, 124.5, 116.6, 115.2, 114.2, 60.6, 34.8, 31.9, 30.9, 14.2. HRMS (FAB) *m/z* 972.5037[M]$^+$ calcd for $C_{60}H_{68}N_4O_8$, found 972.5055.

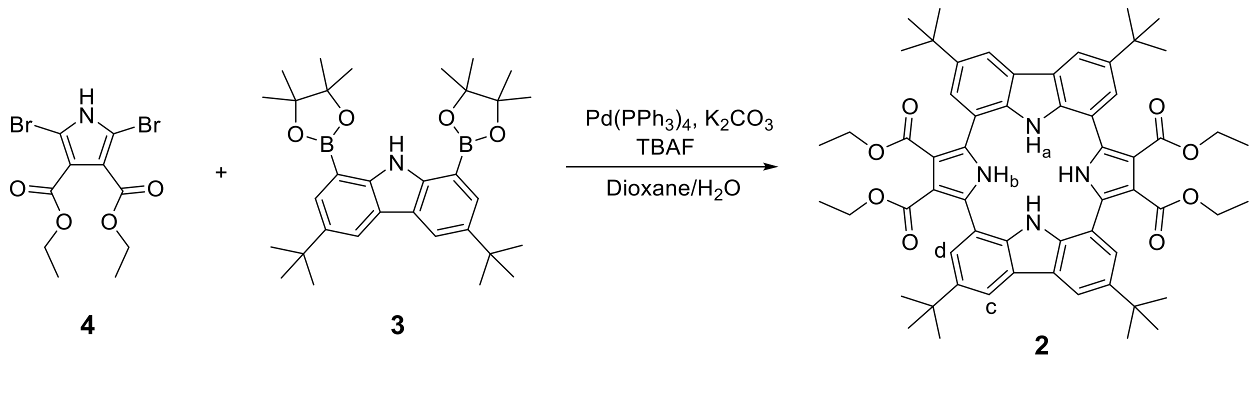

2. ^{1}H NMR spectral data for titrations with anions

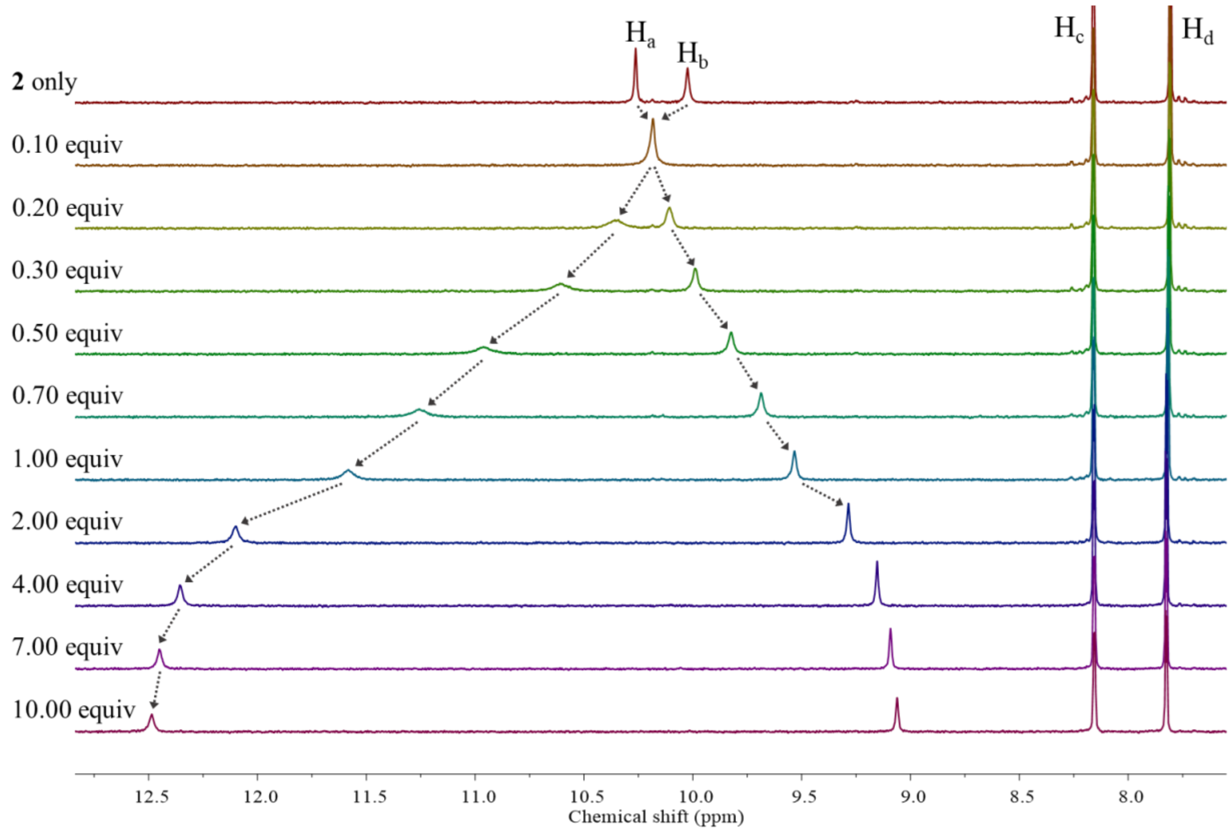

Figure S1. Partial ^{1}H NMR spectra recorded during the titration of receptor **2** (3 mM) with tetrabutylammonium bromide (TBABr) in CD_2Cl_2.

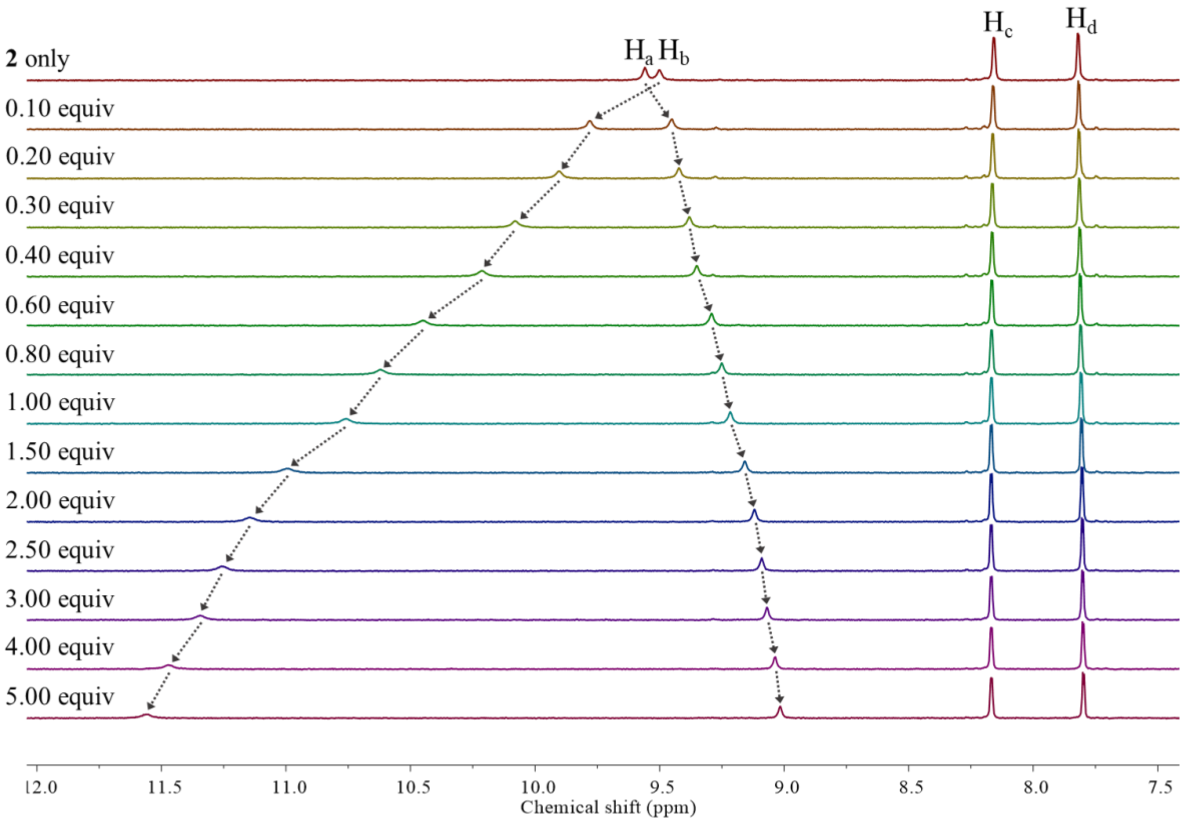

Figure S2. Partial ^{1}H NMR spectra recorded during the titration of receptor **2** (3 mM) with tetrabutylammonium hydrogen sulfate (TBAHSO$_4$) in CD_2Cl_2.

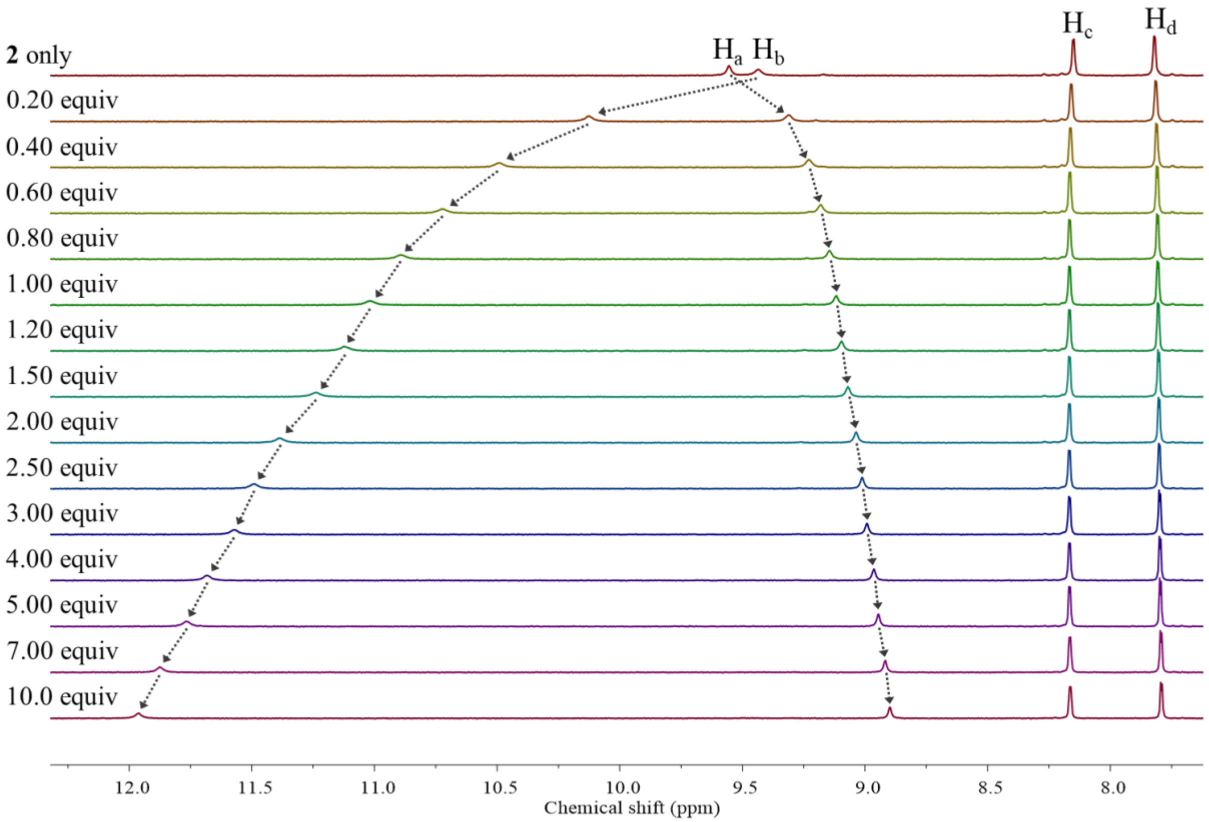

Figure S3. Partial ^{1}H NMR spectra recorded during the titration of receptor **2** (3 mM) with bis(tetrabutylammonium) sulfate ($(TBA)_2 \bullet SO_4$) in CD_2Cl_2

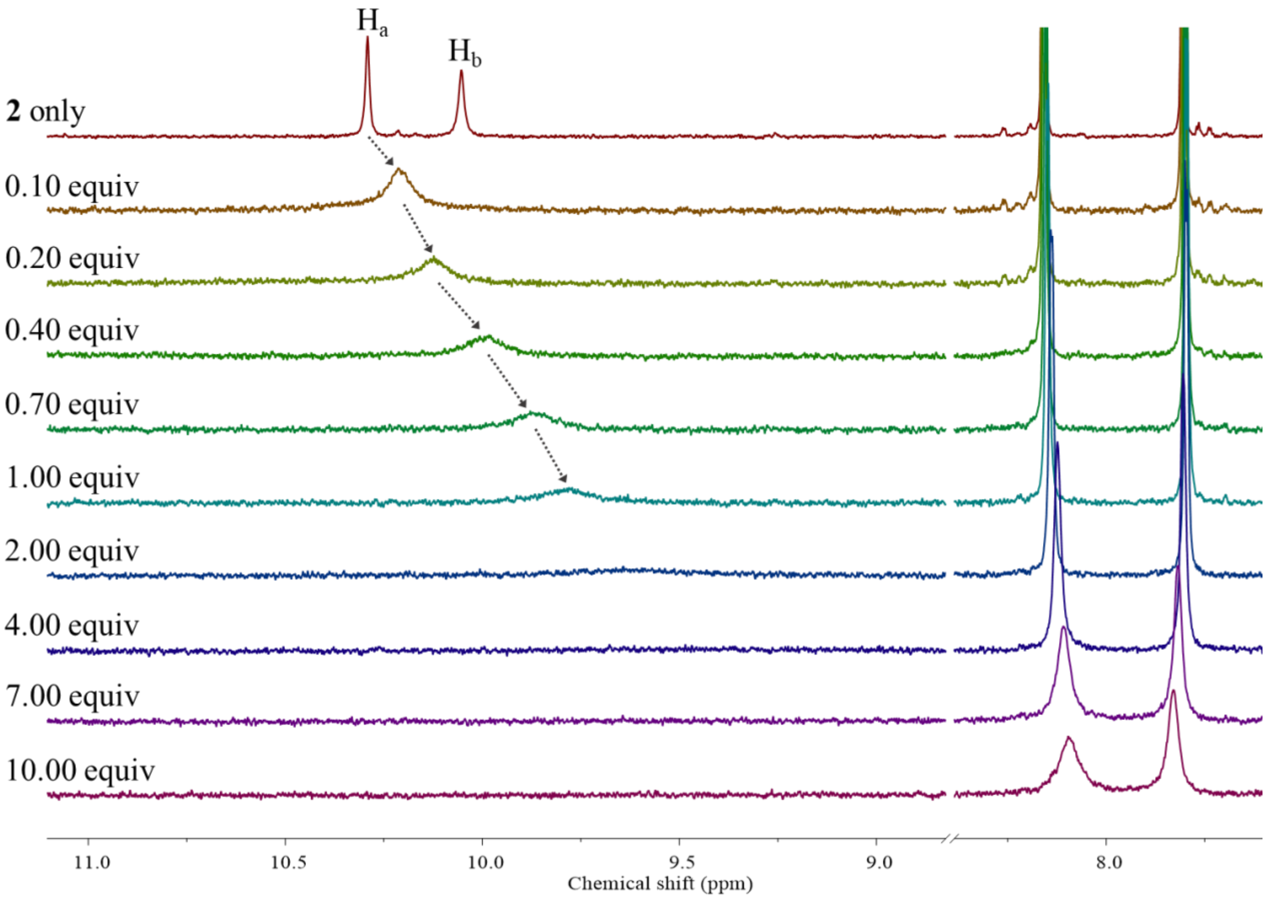

Figure S4. Partial ^{1}H NMR spectra recorded during the titration of receptor **2** (3 mM) with tetrabutylammonium dihydrogen phosphate ($TBAH_2PO_4$) in CD_2Cl_2

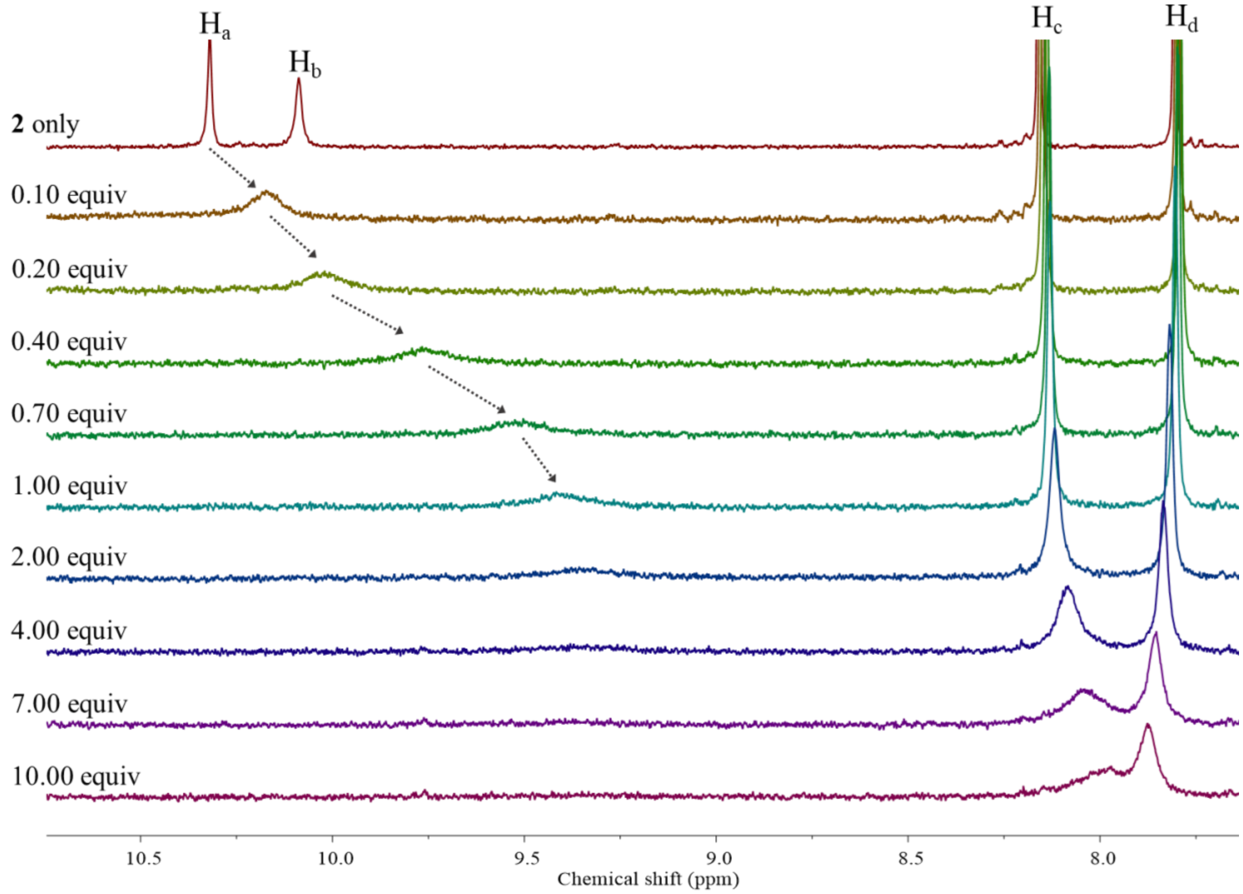

Figure S5. Partial ^{1}H NMR spectra recorded during the titration of receptor **2** (3 mM) with tris(tetrabutylammonium) hydrogen pyrophosphate ($(TBA)_3{\bullet}HP_2O_7$) in CD_2Cl_2.

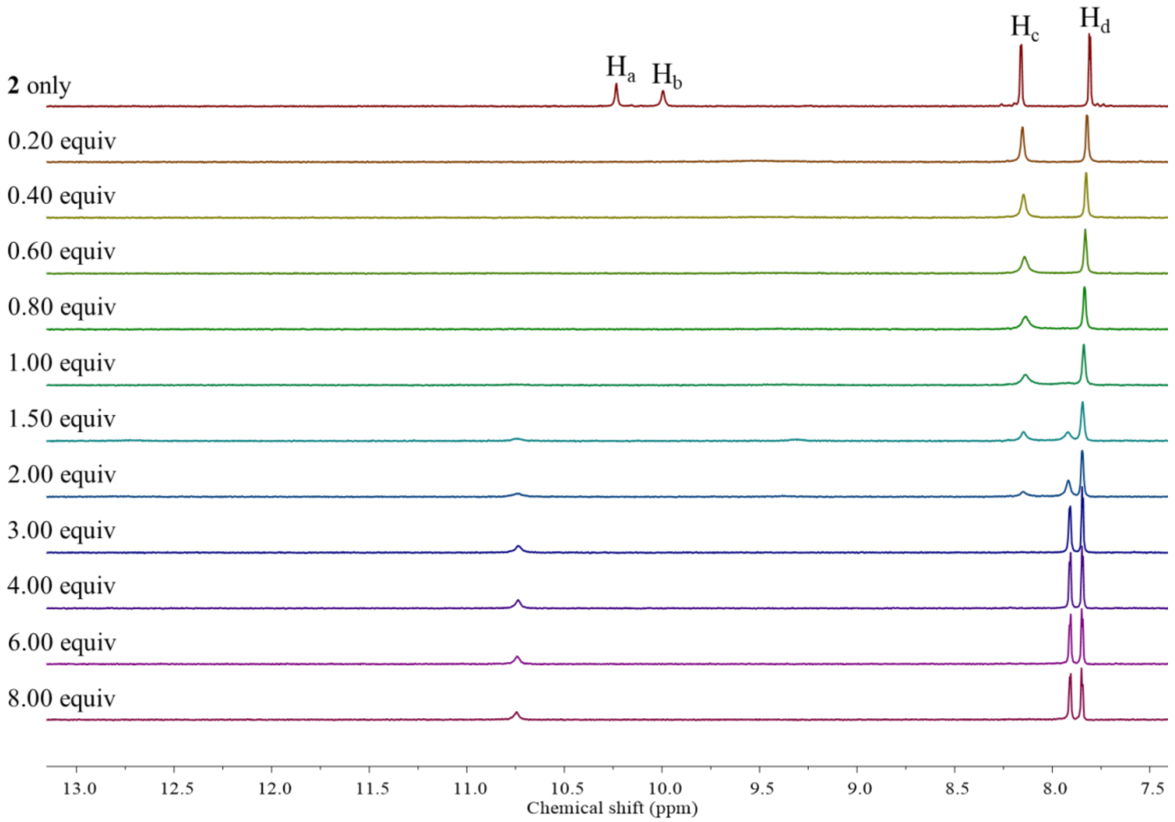

Figure S6. Partial ^{1}H NMR spectra recorded during the titration of receptor **2** (3 mM) with tetraethylammonium bicarbonate ($TEAHCO_3$) in CD_2Cl_2.

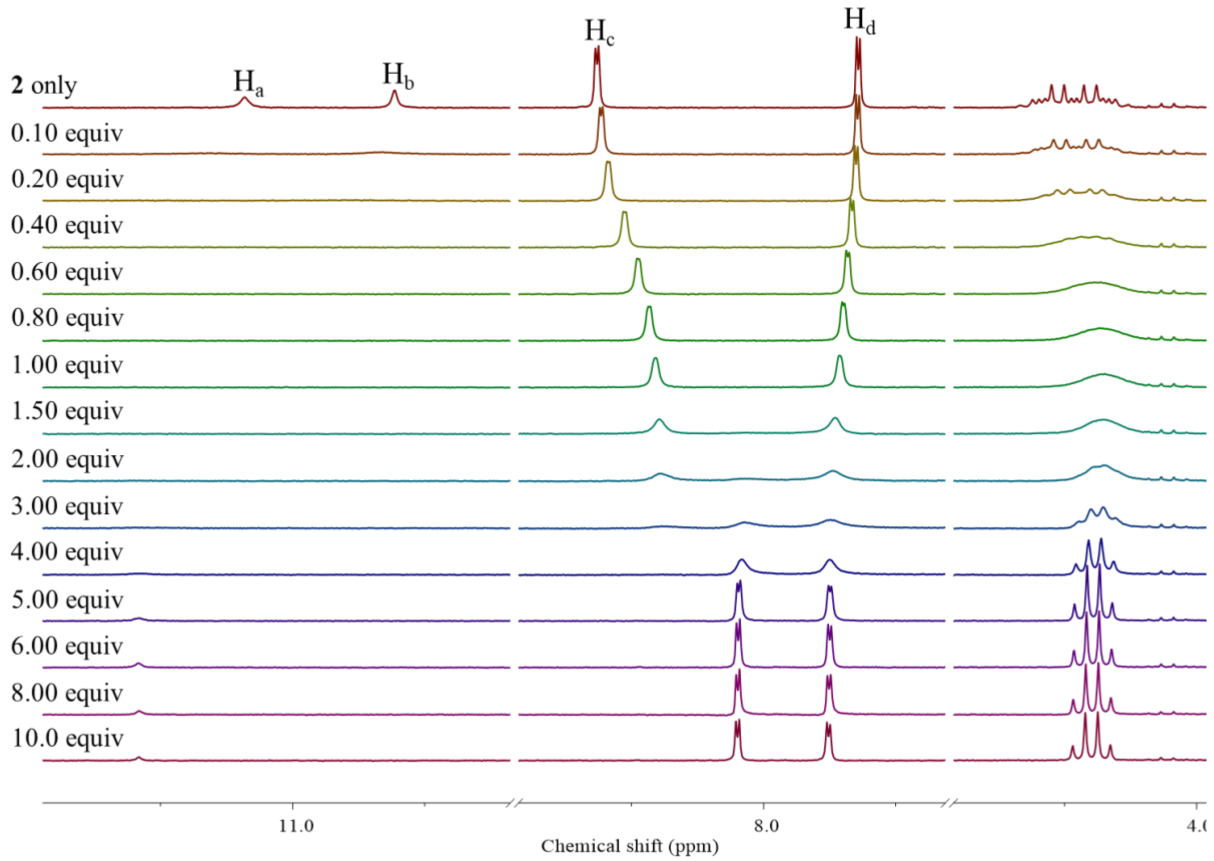

Figure S7. Partial ^{1}H NMR spectra recorded during the titration of receptor **2** (3 mM) with tetrabutylammonium fluoride (TBAF) in acetone-d_6.

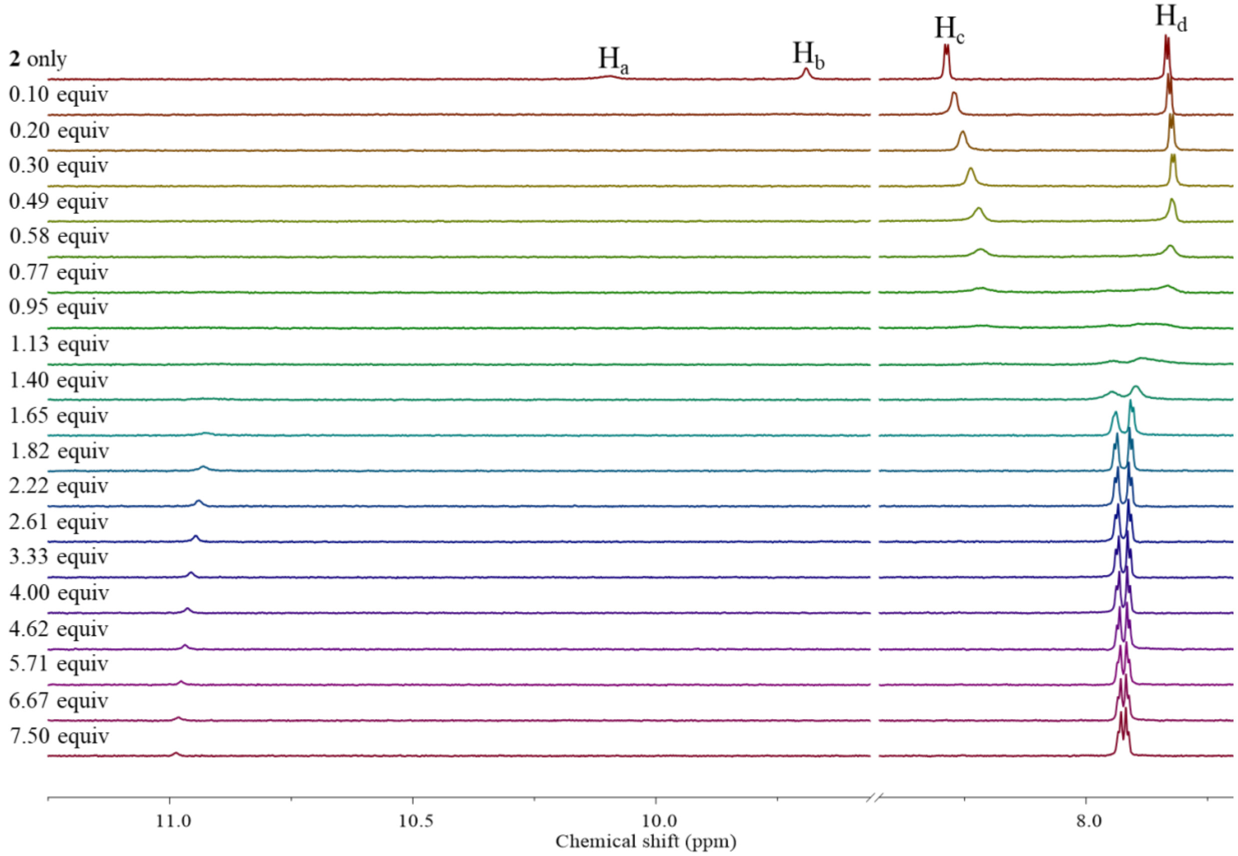

Figure S8. Partial ^{1}H NMR spectra recorded during the titration of receptor **2** (3 mM) with tetrabutylammonium fluoride (TBAF) in CD_3CN.

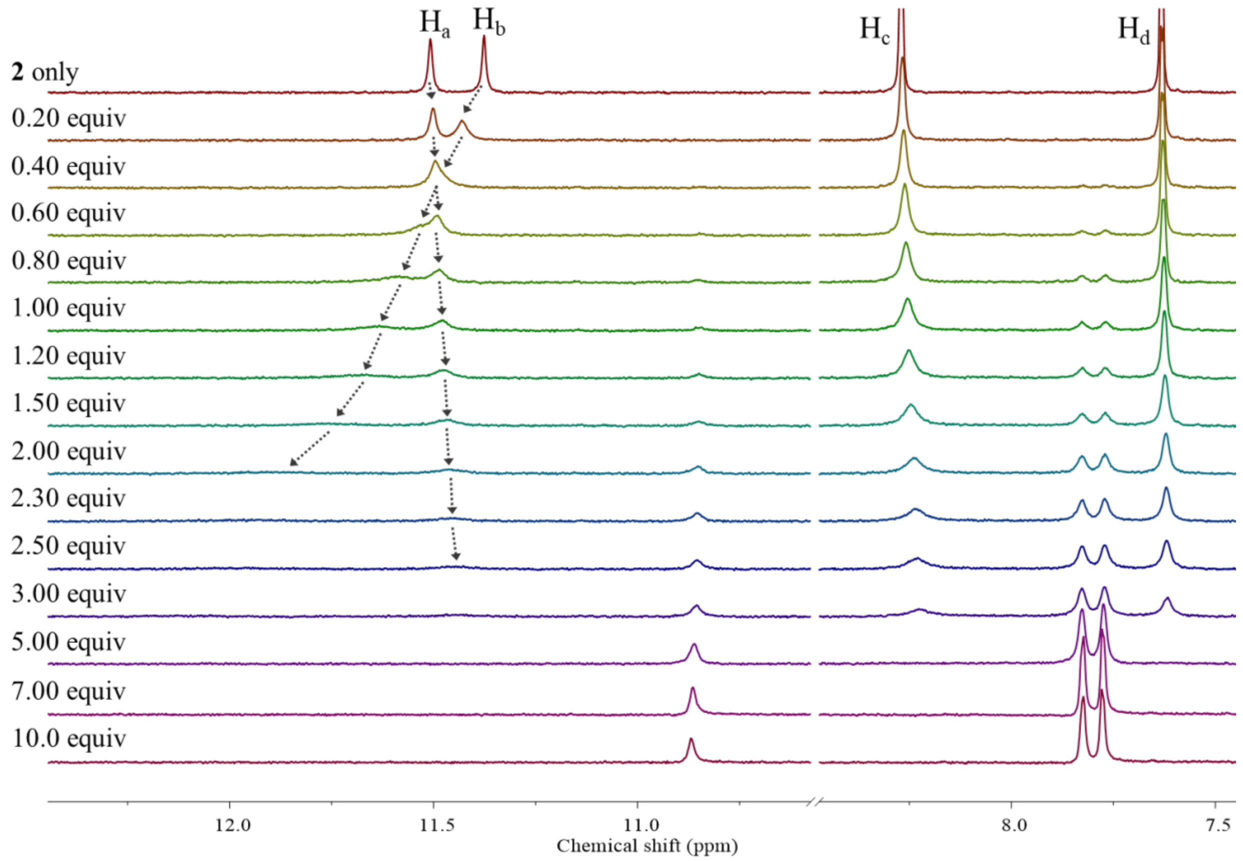

Figure S9. Partial ^{1}H NMR spectra recorded during the titration of receptor **2** (3 mM) with tetrabutylammonium fluoride (TBAF) in DMSO-d_6.

3. UV-vis / Fluorescence spectral data for titrations with anions

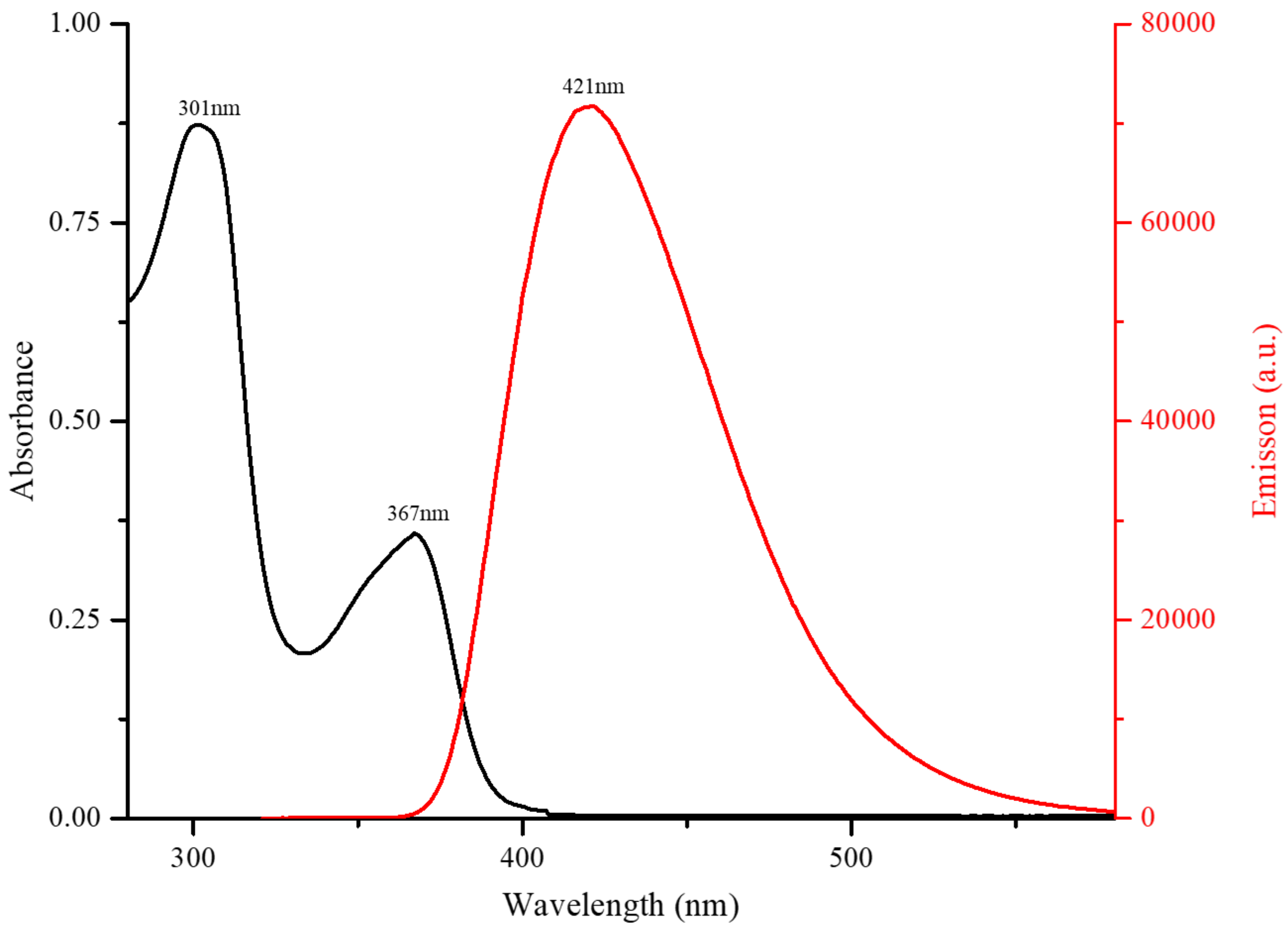

Figure S10. Absorption and emission spectra of receptor **2** (10 μM) in CH_2Cl_2. The excitation wavelength was 301 nm.

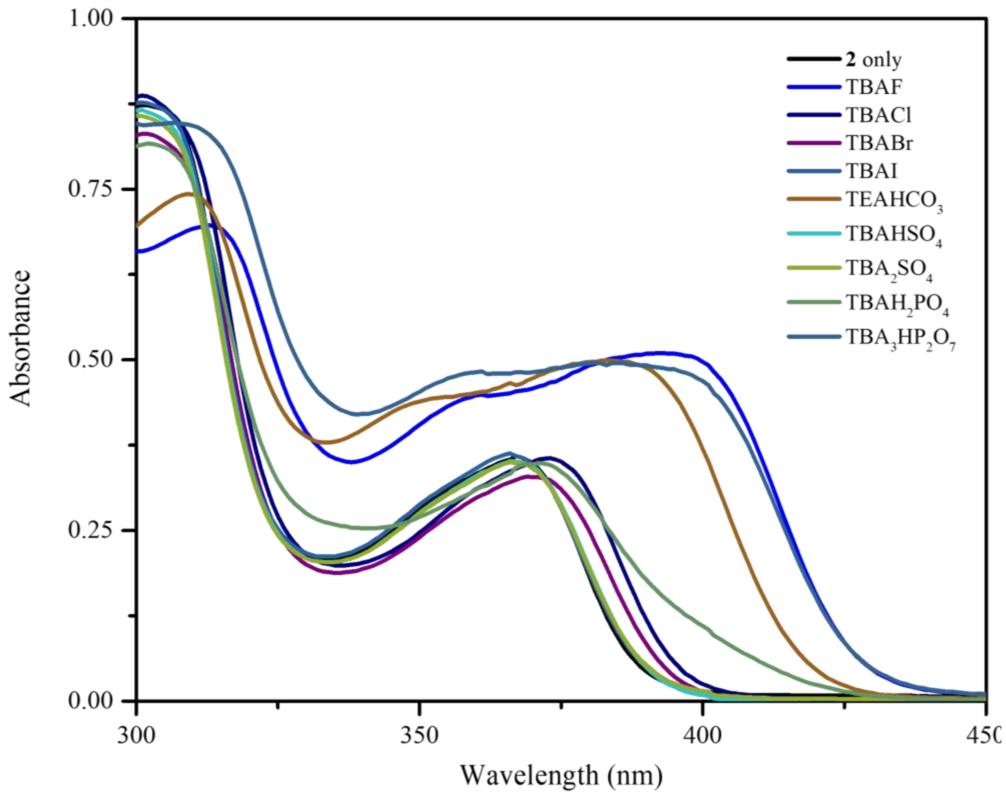

Figure S11. UV-vis spectra of receptor **2 (**10 μM) recorded in the presence of various anion salts in CH_2Cl_2.

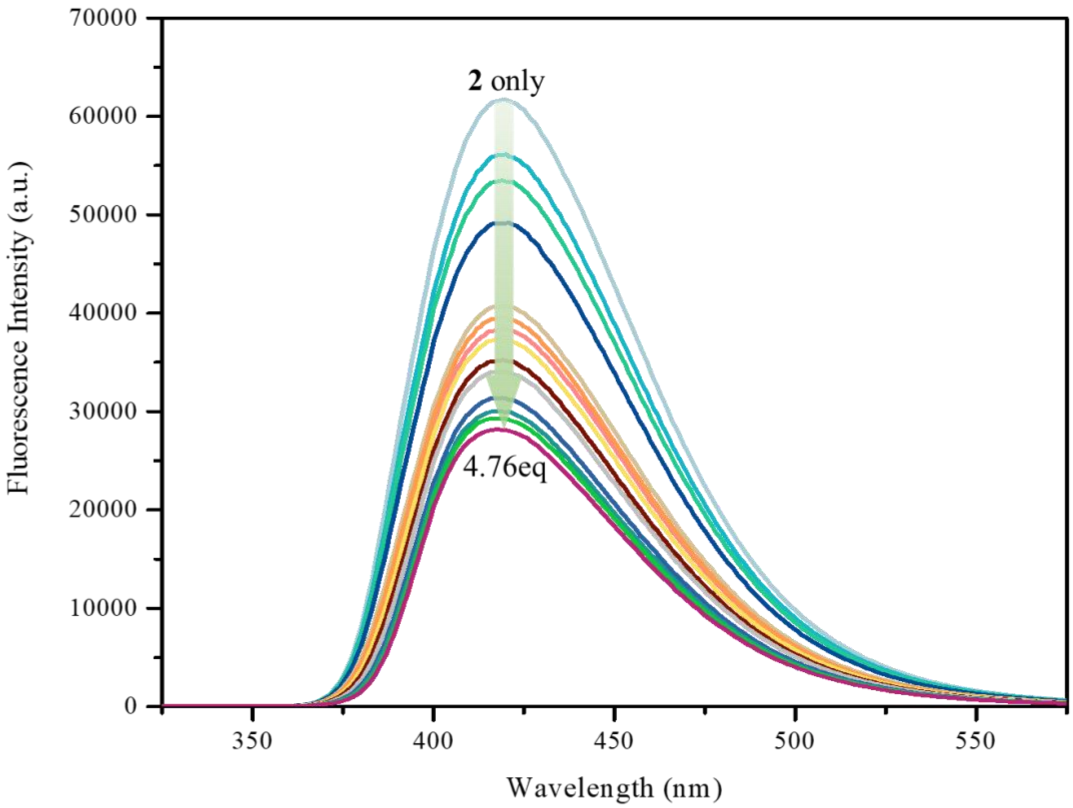

Figure S12. Fluorescence spectra of receptor **2 (**10 μM) recorded during the titration with tetrabutylammonium chloride (TBACl) in CH_2Cl_2. The excitation wavelength was 301 nm.

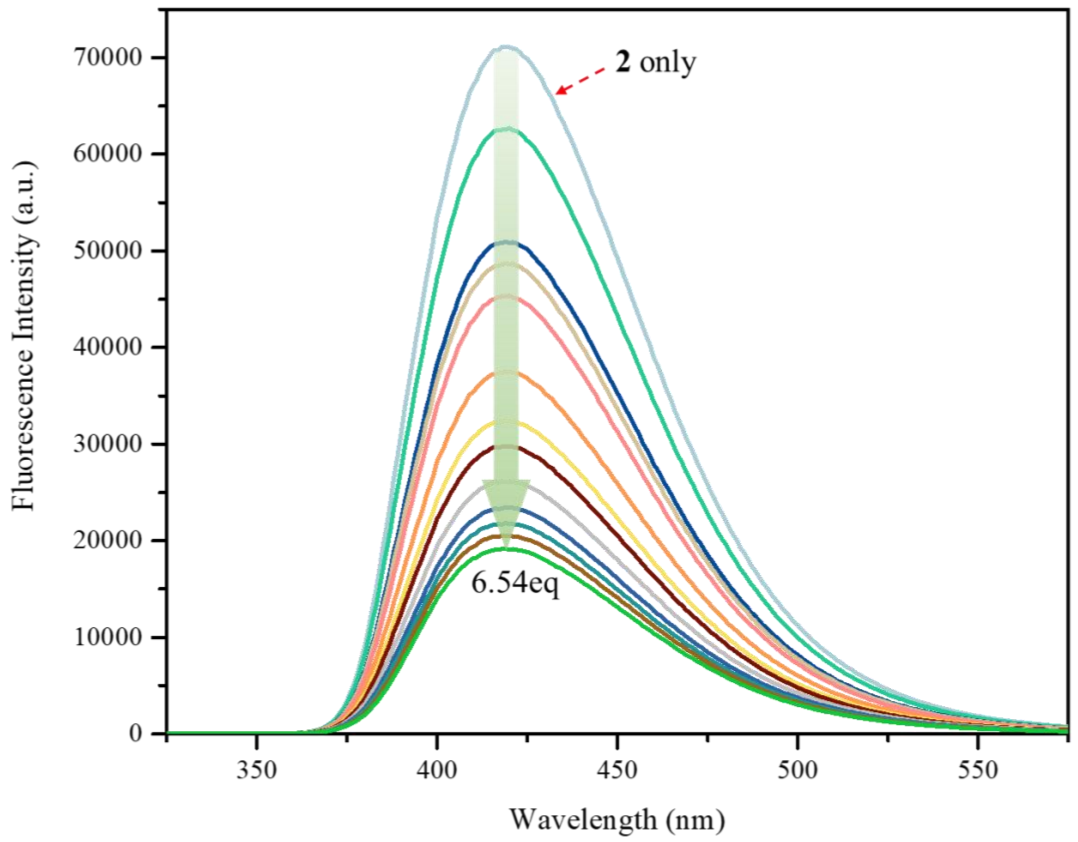

Figure S13. Fluorescence spectra of receptor **2** (10 μM) recorded during the titration with tetrabutylammonium bromide (TBABr) in CH_2Cl_2. The excitation wavelength was 301 nm.

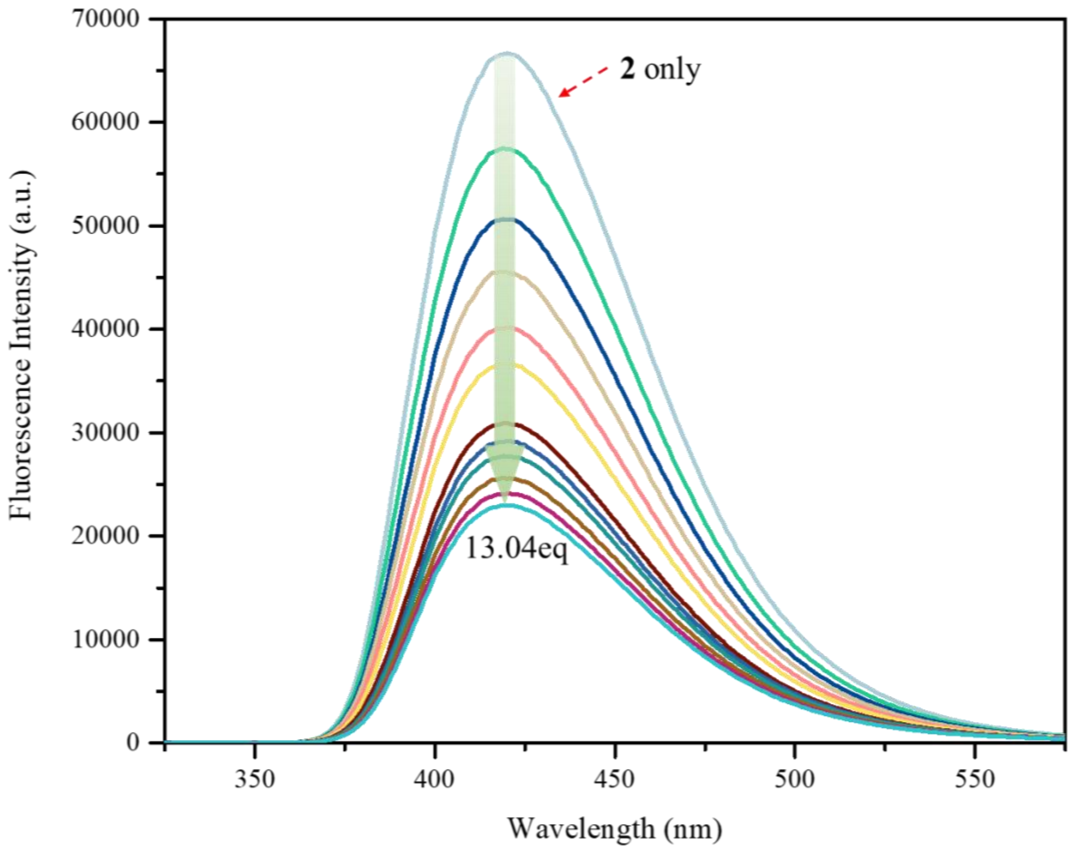

Figure S14. Fluorescence spectra of receptor **2** (10 μM) recorded during a titration with tetrabutylammonium iodide (TBAI) in CH_2Cl_2.

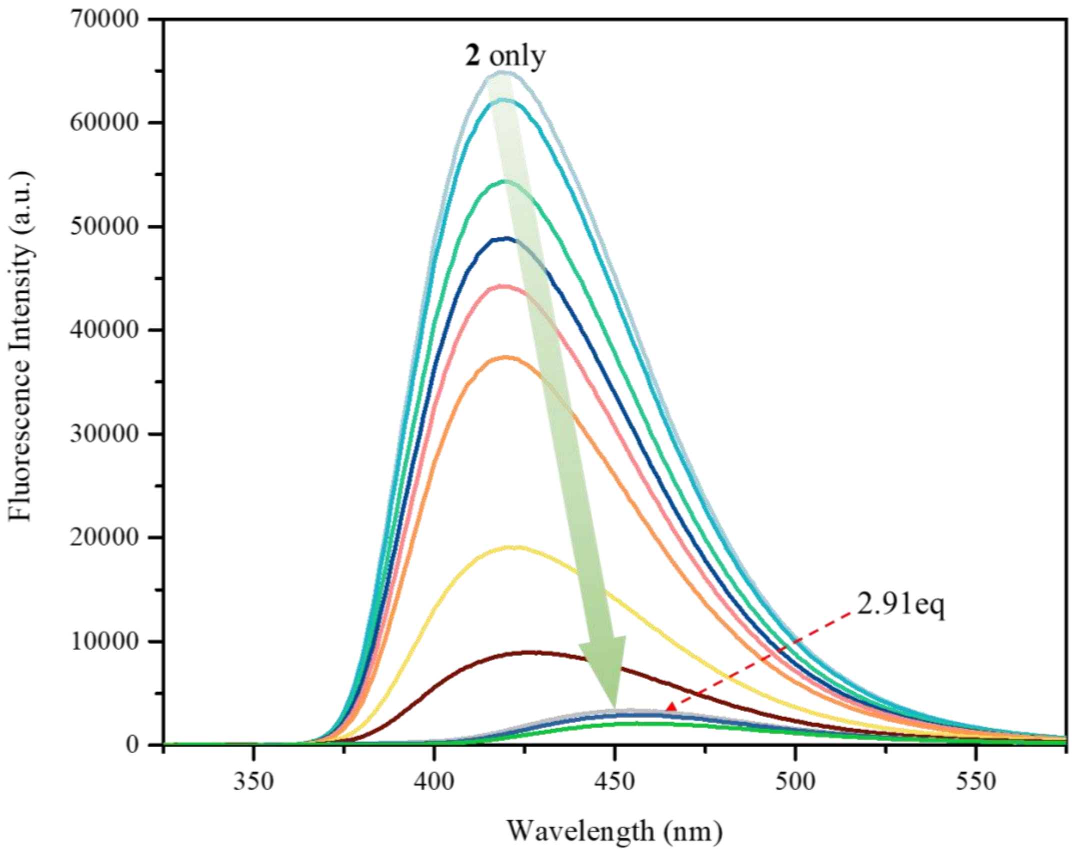

Figure S15. Fluorescence spectra of receptor **2** (10 μM) recorded during a titration with tetraethylammonium bicarbonate ($TEAHCO_3$) in CH_2Cl_2.

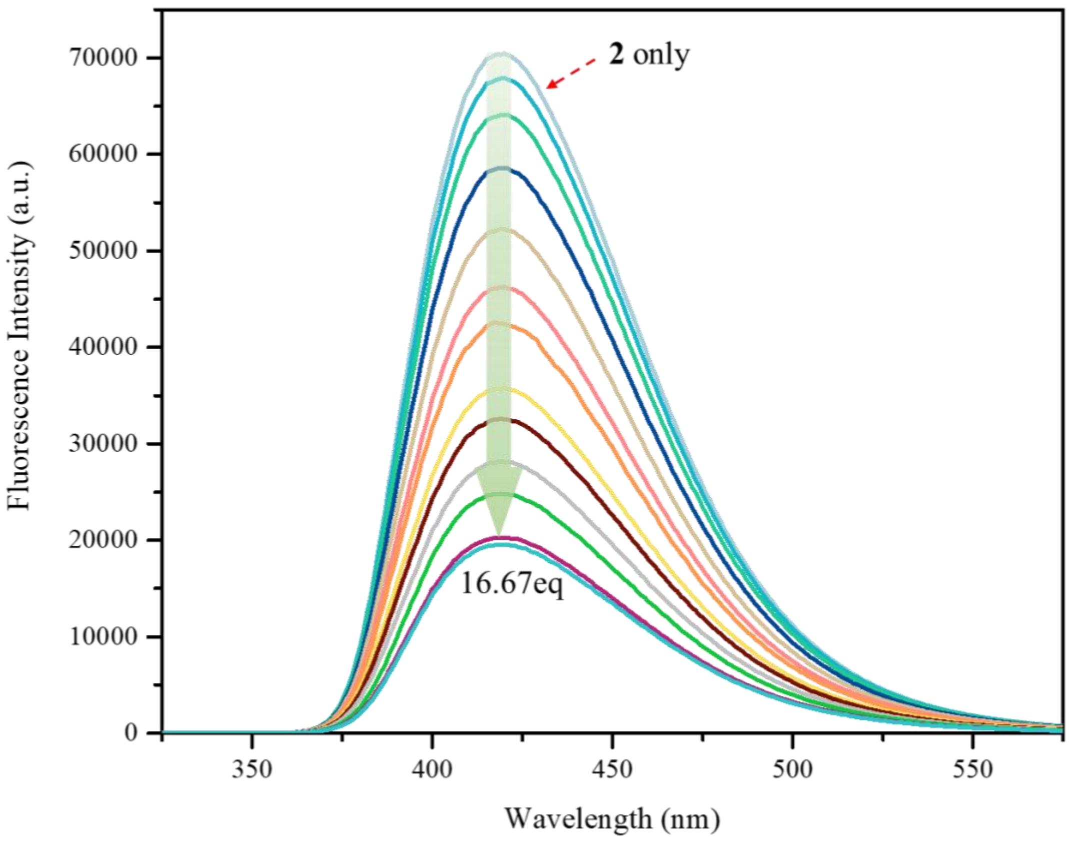

Figure S16. Fluorescence spectra of receptor **2** (10 μM) recorded during a titration with tetrabutylammonium hydrogen sulfate ($TBAHSO_4$) in CH_2Cl_2.

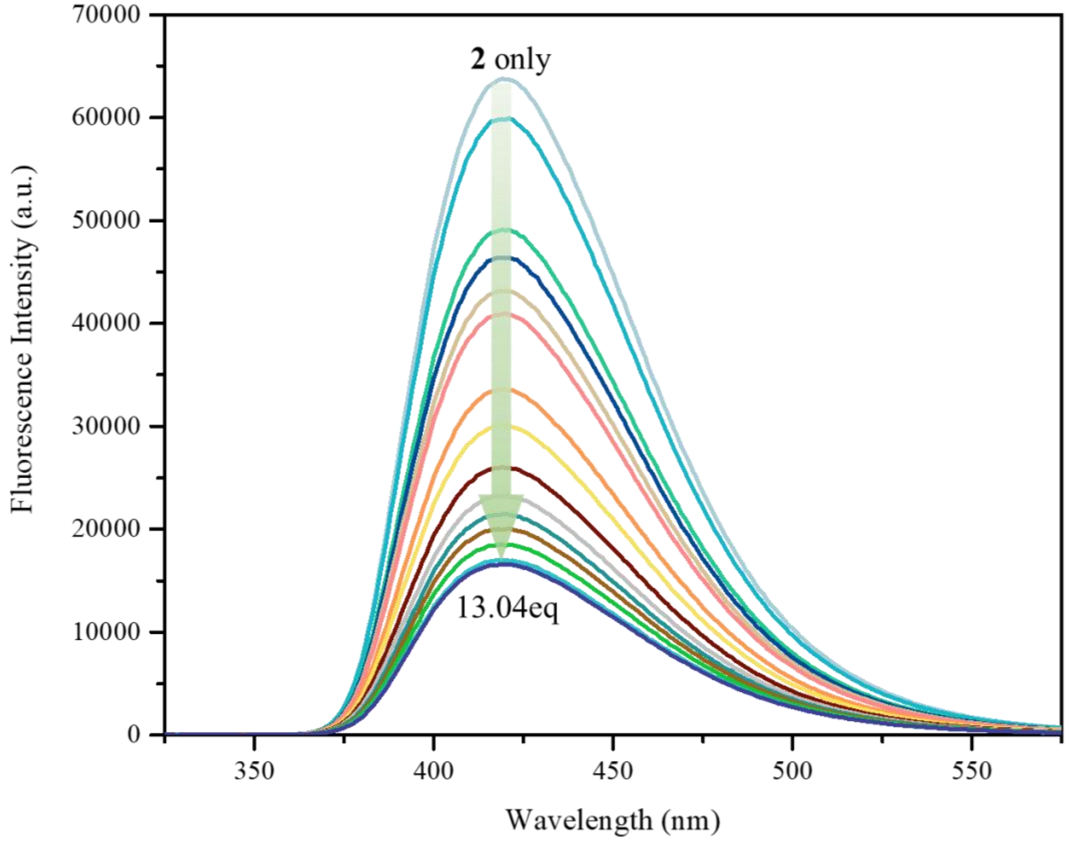

Figure S17. Fluorescence spectra of receptor **2** (10 μM) recorded during a titration with bis(tetrabutylammonium) sulfate ($(TBA)_2 \bullet SO_4$) in CH_2Cl_2.

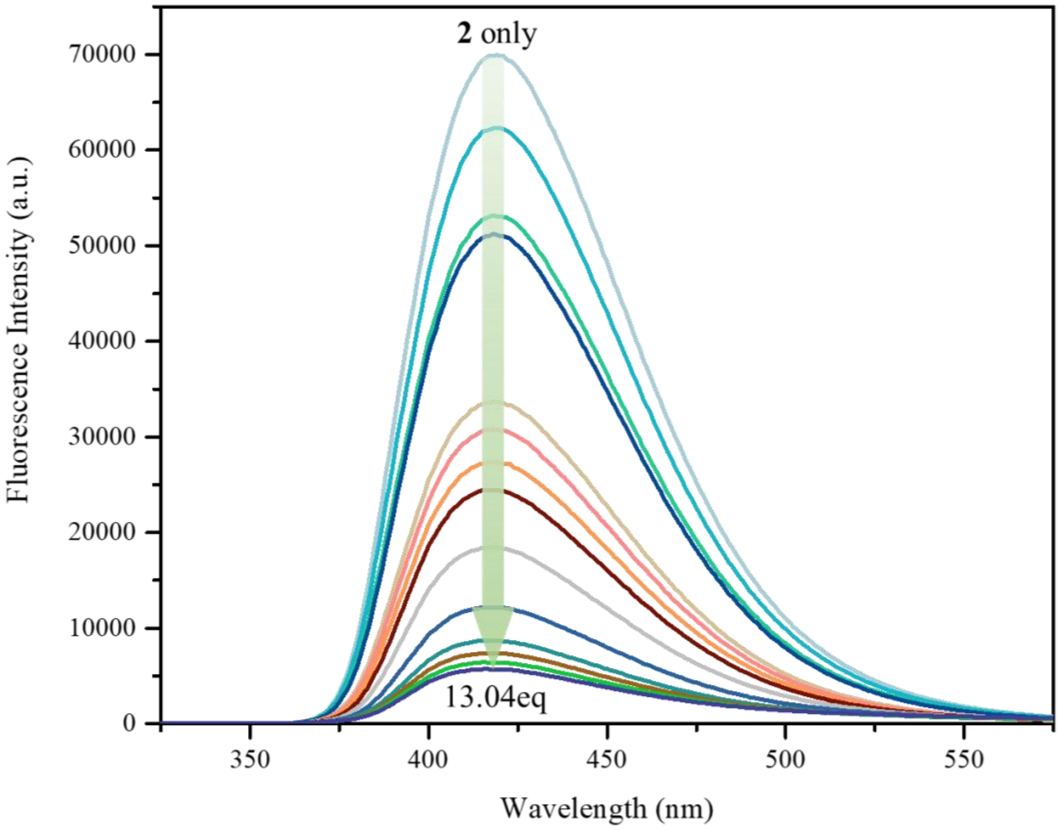

Figure S18. Fluorescence spectra of receptor **2** (10 μM) recorded during a titration with tris(tetraethylammonium) hydrogen pyrophosphate ($(TBA)_3 \bullet HP_2O_7$)) in CH_2Cl_2.

4. Single crystal X-ray diffraction data

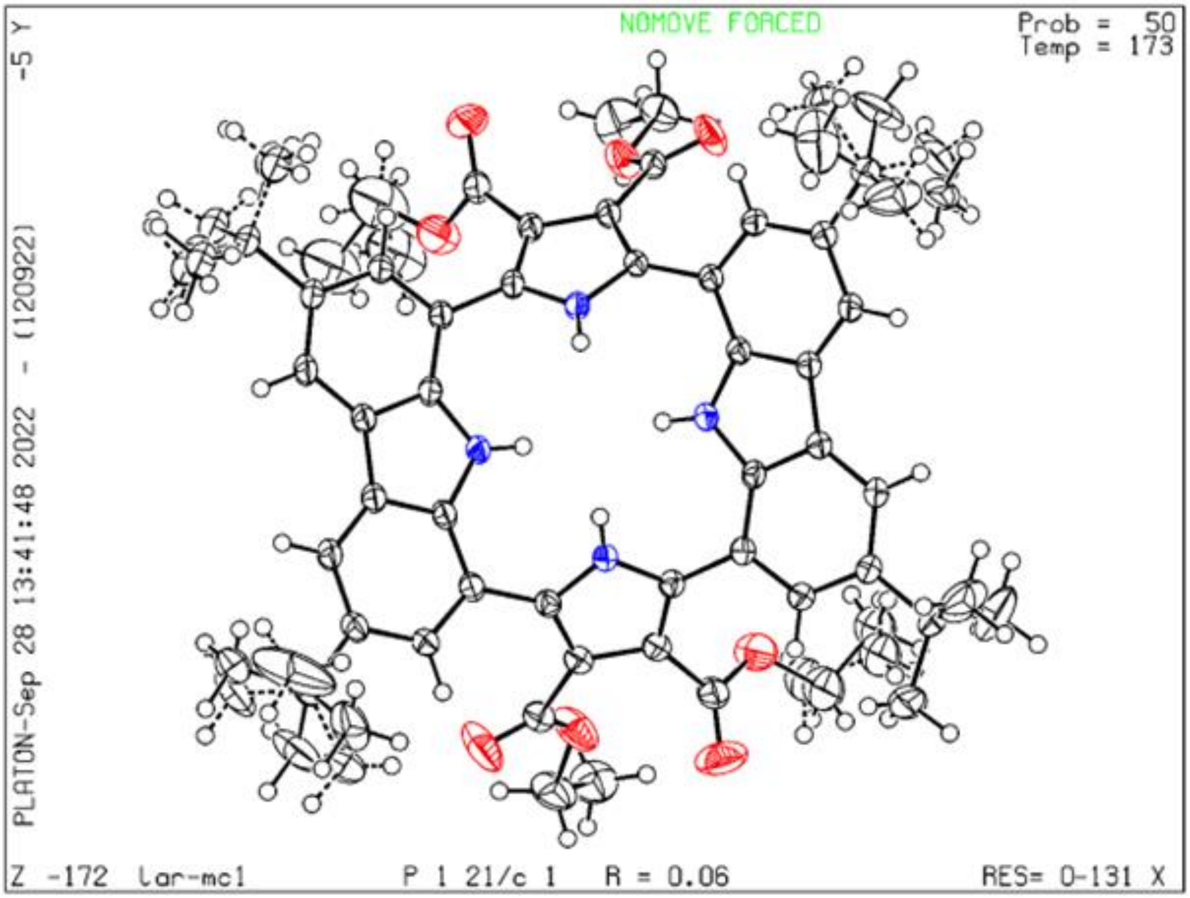

Figure S19. Single crystal X-ray diffraction structure of receptor **2**. Solvent molecules have been omitted for clarity. Displacement ellipsoids are scaled to the 50% probability level.

Table S1. Crystal data and structure refinement for **2**.

Empirical formula	C60 H68 N4 O8	
Formula weight	973.18	
Temperature	173.15 K	
Wavelength	0.71073 Å	
Crystal system	monoclinic	
Space group	P 1 21/c 1	
Unit cell dimensions	a = 15.9159(3) Å	α = 90°.
	b = 21.2730(4) Å	β = 107.1160(10) °.
	c = 18.6760(3) Å	γ = 90°.
Volume	6043.25(19) $Å^3$	
Z	4	
Density (calculated)	1.070 Mg/m^3	
Absorption coefficient	0.071 mm^{-1}	
F(000)	2080	
Crystal size	0.412 x 0.327 x 0.289 mm^3	
Theta range for data collection	2.22 to 26.34°.	
Index ranges	-21<=h<=21, -28<=k<=28, -24<=l<=21	
Reflections collected	14976	
Independent reflections	9703 [R(int) = 0.0423]	
Completeness to theta = 56.6°	99.4 %	
Absorption correction	Multi-scan	
Max. and min. transmission	0.7457 and 0.7114	
Refinement method	Full-matrix least-squares on F^2	
Data / restraints / parameters	14976 / 12 / 729	
Goodness-of-fit on F^2	1.042	
Final R indices [I>2sigma(I)]	R1 = 0.0631, wR2 = 0.1663	
R indices (all data)	R1 = 0.0894, wR2 = 0.1846	
Extinction coefficient	n/a	
Largest diff. peak and hole	0.583 and -0.604 e.$Å^{-3}$	
ccdc number	2219418	

X-ray experimental for [**2**-2H]$^{2-}$•2(TBA$^+$): Crystals grew as clusters of colorless prisms by slow evaporation from acetone and diethyl ether. The data crystal was cut from a larger crystal and had approximate dimensions; 0.22 x 0.12 x 0.061 mm. The data were collected on a Rigaku Oxford Diffraction HyPix6000E Synergy diffractometer using a μ-focus Cu Kα radiation source (λ = 1.5418 Å) with collimating mirror monochromators. A total of 2154 frames of data were collected using ω-scans with a scan range of 0.5° and a counting time of 8 seconds per frame for frames collected with a detector offset of +/- 47.8° and 31 seconds per frame with frames collected with a detector offset of +/- 104.5°. The data were collected at 100 K using an Oxford Cryostream low temperature device. Details of crystal data, data collection and structure refinement are listed in Table S2. Data collection, unit cell refinement and data reduction were performed using Rigaku Oxford Diffraction's CrysAlisPro V 1.171.41.123a.[5] The structure was solved by direct methods using SHELXT[2] and refined by full-matrix least-squares on F[6] with anisotropic displacement parameters for the non-H atoms using SHELXL-2018/3.[7] tructure analysis was aided by use of the programs PLATON[8] and OLEX2.[9] The hydrogen atoms on the carbon atoms were calculated in ideal positions with isotropic displacement parameters set to 1.2xUeq of the attached atom (1.5xUeq for methyl hydrogen atoms). The hydrogen atoms on the nitrogen atoms were observed in a ΔF map and refined with isotropic displacement parameters. The data crystal was twinned. The twin law and twin fractions were determined using CrysAlisPro.

The function, $\Sigma w(|F_O|^2 - |F_C|^2)^2$, was minimized, where $w = 1/[(\sigma(F_O))^2 + (0.0631*P)^2 + (1.6026*P)]$ and $P = (|F_O|^2 + 2|F_C|^2)/3$. $R_W(F^2)$ refined to 0.112, with R(F) equal to 0.0422 and a goodness of fit, S, = 1.04. Definitions used for calculating R(F), $R_W(F^2)$ and the goodness of fit, S, are given below.[10] The data were checked for secondary extinction effects but no correction was necessary. Neutral atom scattering factors and values used to calculate the linear absorption coefficient are from the International Tables for X-ray Crystallography (1992).[11] All figures were generated using SHELXTL/PC.[12] Tables of positional and thermal parameters, bond lengths and angles, torsion angles and figures may be obtained from the Cambridge Crystallographic Centre by referencing CCDC number 2219688.

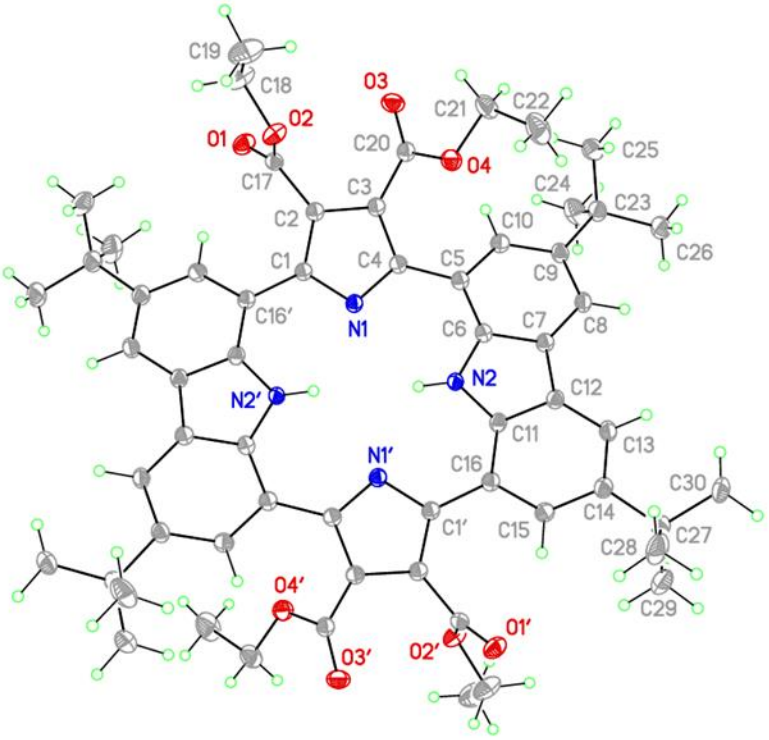

Figure S20. View of macrocycle 1 in [**2**-2H]$^{2-}$•2(TBA^{+}) showing the atom labeling scheme. Solvent molecules and two tetrabutylammonium cations have been omitted for clarity. Displacement ellipsoids are scaled to the 50% probability level. The macrocycle resides around a crystallographic inversion center at ½, ½, ½. Atoms related by 1-x, 1-y, 1-z have labels appended by α ‘.

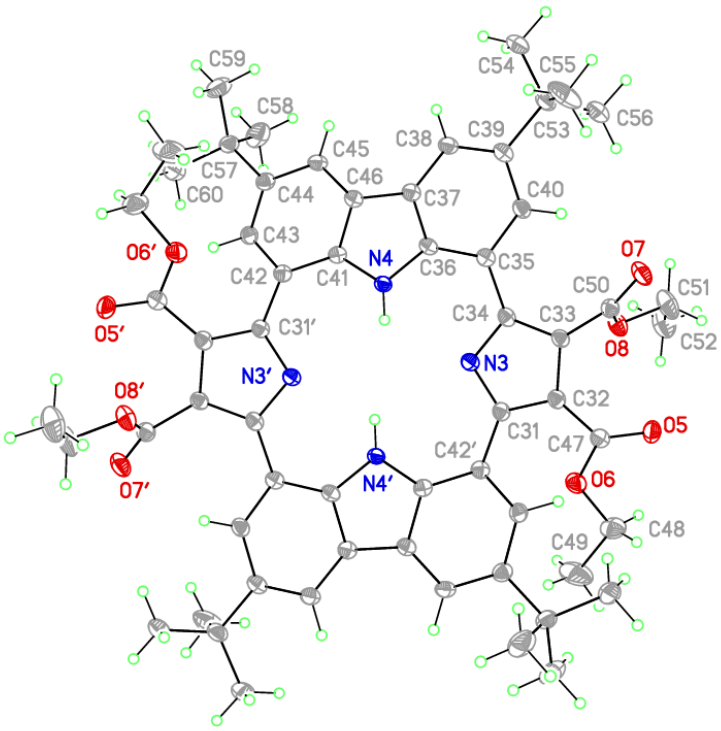

Figure S21. View of macrocycle 2 in [**2**-2H]$^{2-}$•2(TBA^{+}) showing the atom labeling scheme. Solvent molecules and two tetrabutylammonium cations have been omitted for clarity. Displacement ellipsoids are scaled to the 50% probability level. The macrocycle resides around a crystallographic inversion center at ½, 1, ½. Atoms related by 1-x, 2-y, 1-z have labels appended by α ‘.

Table S2. Crystal data and structure refinement for [**2**-2H]$^{2-}$•2(TBA^{+}).

Empirical formula	C96 H148 N6 O9	
Formula weight	1530.20	
Temperature	100.0(6) K	
Wavelength	1.54184 Å	
Crystal system	monoclinic	
Space group	P 1 21/n 1	
Unit cell dimensions	a = 17.4296(3) Å	a= 90°.
	b = 29.0372(5) Å	b= 91.2569(12)°.
	c = 18.0368(2) Å	g = 90°.
Volume	9126.4(2) Å^3	
Z	4	
Density (calculated)	1.114 Mg/m^3	
Absorption coefficient	0.548 mm^{-1}	
F(000)	3352	
Crystal size	0.22 x 0.12 x 0.061 mm^3	
Theta range for data collection	2.885 to 76.479°.	
Index ranges	-21<=h<=21, -35<=k<=35, -22<=l<=22	
Reflections collected	30978	
Independent reflections	30978 [R(int) = ?]	
Completeness to theta = 67.684°	99.3 %	
Absorption correction	Semi-empirical from equivalents	
Max. and min. transmission	1.00000 and 0.80527	
Refinement method	Full-matrix least-squares on F^2	
Data / restraints / parameters	30978 / 16 / 1055	
Goodness-of-fit on F^2	1.035	
Final R indices [I>2sigma(I)]	R1 = 0.0422, wR2 = 0.1080	
R indices (all data)	R1 = 0.0496, wR2 = 0.1120	
Extinction coefficient	n/a	
Largest diff. peak and hole	0.290 and -0.410 e.Å^{-3}	
ccdc number	2219688	

5. NMR and HRMS Spectra

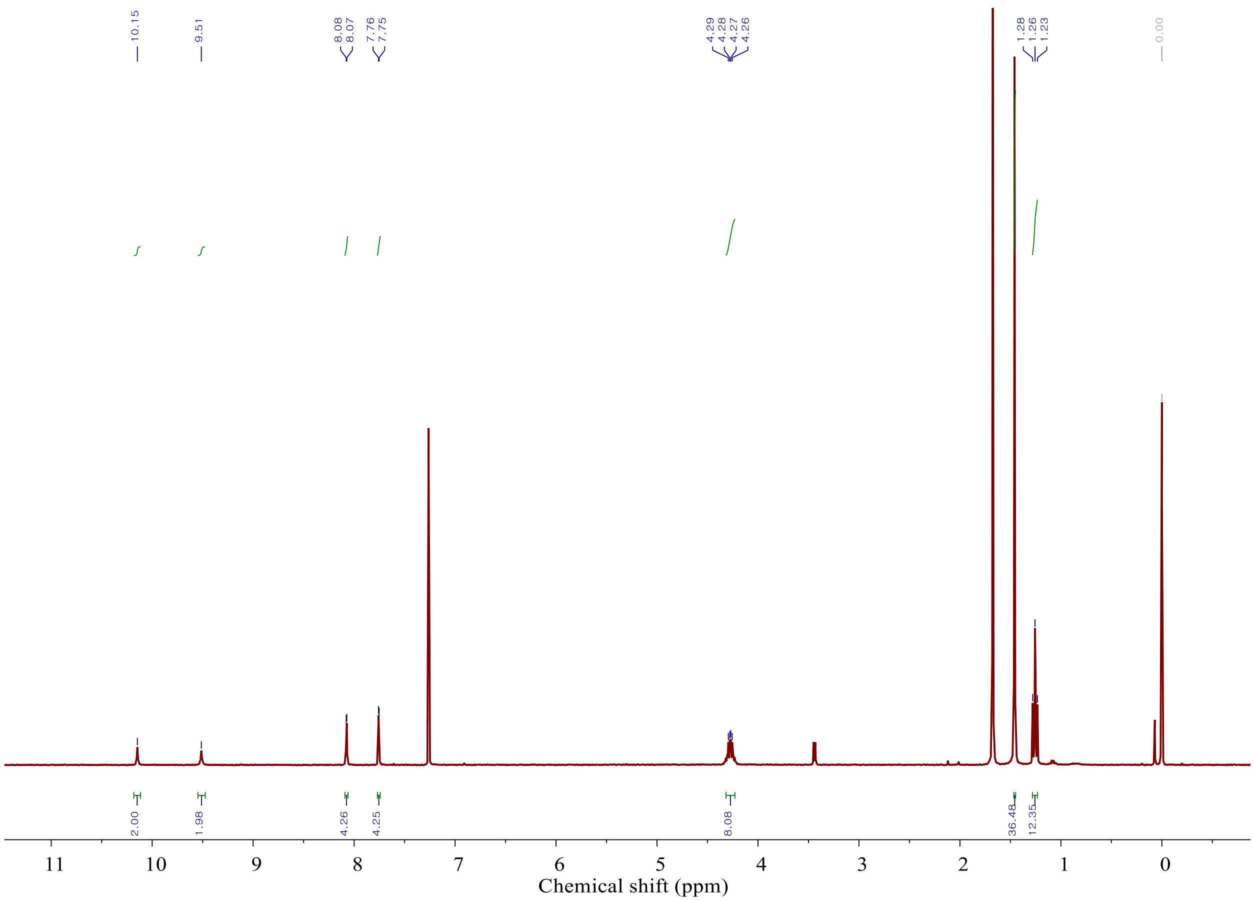

Figure S21. ^{1}H NMR spectrum of receptor **2** recorded in $CDCl_3$.

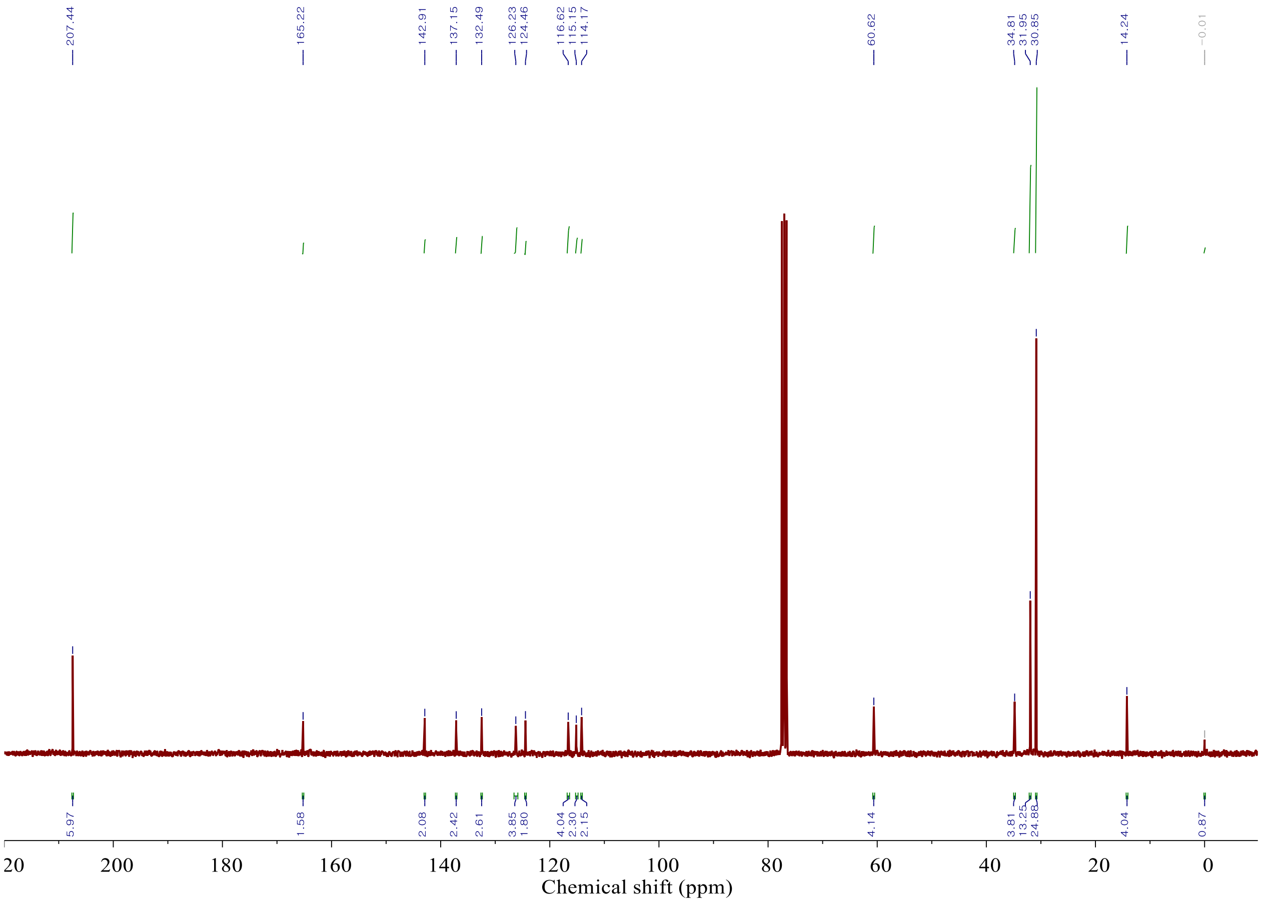

Figure S22. ^{13}C NMR spectrum of receptor **2** recorded in $CDCl_3$.

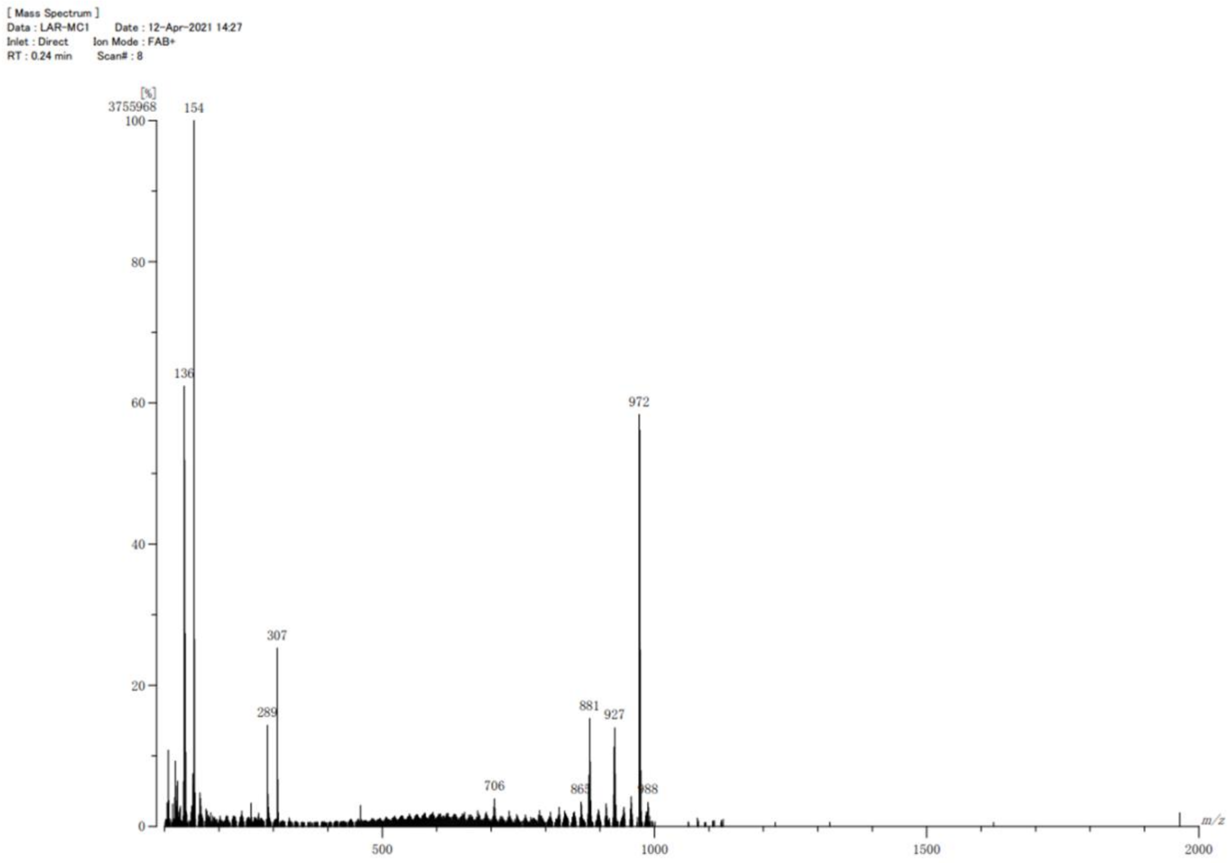

Figure S12. FAB mass spectrum of receptor **2**.

Data : LAR-MC1_HR Date : 12-Apr-2021 16:09
Instrument : MStation
Sample : -
Note : -
Inlet : Direct Ion Mode : FAB+
RT : 0.27 min Scan# : 9
Elements : C 100/1, H 100/1, N 10/1, O 10/1
Mass Tolerance : 1000ppm, 3mmu if m/z > 3
Unsaturation (U.S.) : -0.5 - 30.0

	Observed m/z	Int%	Err[ppm / mmu]	U.S.	Composition
1	972.5055	100.00	+1.8 / +1.8	29.0	C60 H68 N4 O8
2			+0.5 / +0.4	28.5	C62 H70 N O9

Figure S24. HR FAB mass spectral data for receptor **2**.

6. References

1) Brewster, J. T.; Zafar, H.; McVeigh, M.; Wight, C. D.; Anguera, G.; Steinbrück, A.; Lynch, V. M.; Sessler, J. L. *J. Org. Chem.* **2018**, *83*, 9568-9570.

2) Arnold, L.; Baumgarten, M.; Müllen, K. *Chem. Commun.* **2012**, *48*, 9640-9642.

3) Conformational searches performed with PCModel, Version 9.3; Serena Software: Bloomington, IN, 2012.

4) T. A. Halgren, *J. Comp. Chem.* **1996**, *17*, 490-519.

5) CrysAlisPro. Rigaku Oxford Diffraction, HyPix6000E System, CrysAlisPro Software System, 1.171.41.123a.

6) Sheldrick. G. M. SHELXT. A program for crystal structure solution. *Acta Cryst. A* **2015**, *71*, 3-8.

7) Sheldrick, G. M. SHELXL-2018/3. Program for the Refinement of Crystal Structures. *Acta Cryst. C* **2015**, *71*, 9-18.

8) Spek, A. L. PLATON, A Multipurpose Crystallographic Tool. Utrecht University, The Netherlands. *Acta Cryst. D* **2009**, *65*, 148-155.

9) Dolomanov, O. V.; Bourhis, L. J.; Gildea, R. J.; Howard, J. A. K.; Puschmann, H. OLEX2: A Complete Structure Solution, Refinement and Analysis Program. *J. Appl. Cryst.* **2009**, *42*, 339-341.

10) $R_W(F^2) = \{\Sigma w(|F_O|^2 - |F_C|^2)^2/\Sigma w(|F_O|)^4\}^{1/2}$ where w is the weight given each reflection.
$R(F) = \Sigma (|F_O| - |F_C|)/\Sigma|F_O|\}$ for reflections with $F_O > 4(\Sigma(F_O))$.
$S = [\Sigma w(|F_O|^2 - |F_C|^2)^2/(n - p)]^{1/2}$, where n is the number of reflections and p is the number of refined parameters.

11) International Tables for X-ray Crystallography (1992). Vol. C, Tables 4.2.6.8 and 6.1.1.4, A. J. C. Wilson, editor, Boston: Kluwer Academic Press.

12) Sheldrick, G. M. (1994). SHELXTL/PC (Version 5.03). Siemens Analytical X-ray Instruments, Inc., Madison, Wisconsin, USA.

```
HEADER    MMFF94 OPTIMIZED HOST
COMPND   LOWEST ENERGY FORM BY SAMPLING IN VACUO MD RUN
AUTHOR    GENERATED BY PCMODEL V 9.30
HETATM    1  N        -0.082   2.528   2.003
HETATM    2  O        -1.379  -4.000  -2.013
HETATM    3  N        -2.230  -0.037   1.639
HETATM    4  N        -0.082  -2.528   2.004
HETATM    5  O        -2.800  -4.828  -0.419
HETATM    6  N         2.026   0.038   1.456
HETATM    7  O         0.865   4.000  -2.110
HETATM    8  O        -2.985   3.538  -0.855
HETATM    9  O         2.417   4.828  -0.644
HETATM   10  C        -1.248   2.841   1.347
HETATM   11  C        -2.896  -1.173   2.033
HETATM   12  C         1.016   2.913   1.273
HETATM   13  C        -1.239  -2.913   1.371
HETATM   14  C        -2.894   1.100   2.033
HETATM   15  C         2.357   2.524   1.694
HETATM   16  C        -4.096  -0.753   2.597
HETATM   17  C        -2.539  -2.523   1.905
HETATM   18  C         2.723   1.174   1.791
HETATM   19  C         2.721  -1.098   1.792
HETATM   20  C        -2.537   2.447   1.903
HETATM   21  C        -3.475  -3.469   2.349
HETATM   22  C        -5.012  -1.737   3.036
HETATM   23  C        -4.095   0.682   2.598
HETATM   24  C        -3.469   3.397   2.352
HETATM   25  C        -4.718  -3.110   2.909
HETATM   26  C         3.966  -0.681   2.251
HETATM   27  C        -0.865  -3.622   0.234
HETATM   28  C         1.023  -2.841   1.250
HETATM   29  C         3.328   3.470   2.056
HETATM   30  C         2.355  -2.447   1.693
HETATM   31  C        -0.897   3.586   0.225
HETATM   32  C        -5.008   1.668   3.037
HETATM   33  C        -4.711   3.041   2.914
HETATM   34  C         4.608  -3.040   2.514
HETATM   35  C        -1.780  -4.224  -0.729
HETATM   36  C         4.614   3.111   2.507
HETATM   37  O        -1.544   5.252  -1.323
HETATM   38  C         3.968   0.754   2.250
HETATM   39  C         0.546   3.622   0.173
```

HETATM	40	C	3.322	-3.397	2.061
HETATM	41	C	4.914	-1.667	2.611
HETATM	42	C	0.577	-3.585	0.162
HETATM	43	C	4.918	1.737	2.608
HETATM	44	C	1.374	4.225	-0.866
HETATM	45	C	-5.690	4.140	3.371
HETATM	46	C	5.622	-4.139	2.885
HETATM	47	C	-1.813	4.219	-0.722
HETATM	48	C	-2.212	-4.607	-3.005
HETATM	49	C	1.609	4.608	-3.170
HETATM	50	C	0.885	4.330	-4.473
HETATM	51	C	-7.009	3.596	3.963
HETATM	52	C	-5.025	5.007	4.461
HETATM	53	C	-1.602	-4.329	-4.365
HETATM	54	C	-6.064	5.032	2.167
HETATM	55	C	5.631	4.210	2.869
HETATM	56	C	-5.700	-4.209	3.358
HETATM	57	C	1.408	-4.219	-0.860
HETATM	58	C	-6.068	-5.100	2.152
HETATM	59	C	-7.024	-3.664	3.941
HETATM	60	C	-5.043	-5.077	4.453
HETATM	61	C	7.000	3.665	3.336
HETATM	62	C	5.894	5.100	1.636
HETATM	63	C	5.071	5.079	4.016
HETATM	64	C	-3.932	4.142	-1.741
HETATM	65	C	-5.177	3.277	-1.756
HETATM	66	C	6.988	-3.595	3.361
HETATM	67	C	5.053	-5.006	4.029
HETATM	68	C	5.892	-5.032	1.654
HETATM	69	O	2.565	-3.538	-1.093
HETATM	70	C	3.432	-4.141	-2.057
HETATM	71	C	4.671	-3.276	-2.178
HETATM	72	O	1.089	-5.252	-1.436
HETATM	73	H	-0.037	2.050	2.894
HETATM	74	H	-1.372	-0.038	1.111
HETATM	75	H	-0.051	-2.049	2.895
HETATM	76	H	1.126	0.038	1.003
HETATM	77	H	-3.218	-4.523	2.246
HETATM	78	H	-5.947	-1.395	3.467
HETATM	79	H	-3.206	4.450	2.257
HETATM	80	H	3.063	4.524	1.974
HETATM	81	H	-5.943	1.325	3.469
HETATM	82	H	3.052	-4.449	1.988
HETATM	83	H	5.882	-1.325	2.961

HETATM	84	H	5.886	1.395	2.957
HETATM	85	H	-3.219	-4.178	-2.955
HETATM	86	H	-2.262	-5.689	-2.837
HETATM	87	H	1.673	5.690	-3.007
HETATM	88	H	2.616	4.179	-3.206
HETATM	89	H	0.795	3.252	-4.641
HETATM	90	H	-0.132	4.734	-4.441
HETATM	91	H	1.417	4.777	-5.317
HETATM	92	H	-6.830	2.978	4.850
HETATM	93	H	-7.672	4.414	4.270
HETATM	94	H	-7.561	2.993	3.232
HETATM	95	H	-4.716	4.396	5.317
HETATM	96	H	-4.135	5.529	4.091
HETATM	97	H	-5.715	5.775	4.830
HETATM	98	H	-0.586	-4.732	-4.422
HETATM	99	H	-2.205	-4.776	-5.161
HETATM	100	H	-1.527	-3.251	-4.541
HETATM	101	H	-6.520	4.441	1.365
HETATM	102	H	-6.783	5.807	2.459
HETATM	103	H	-5.194	5.546	1.743
HETATM	104	H	-6.507	-4.508	1.342
HETATM	105	H	-5.197	-5.624	1.742
HETATM	106	H	-6.797	-5.869	2.437
HETATM	107	H	-7.569	-3.059	3.207
HETATM	108	H	-7.689	-4.482	4.242
HETATM	109	H	-6.850	-3.048	4.831
HETATM	110	H	-4.150	-5.597	4.090
HETATM	111	H	-4.741	-4.467	5.312
HETATM	112	H	-5.735	-5.846	4.816
HETATM	113	H	6.903	3.049	4.238
HETATM	114	H	7.480	3.059	2.559
HETATM	115	H	7.689	4.483	3.579
HETATM	116	H	6.645	5.869	1.856
HETATM	117	H	6.262	4.507	0.791
HETATM	118	H	4.991	5.624	1.300
HETATM	119	H	4.845	4.468	4.898
HETATM	120	H	5.792	5.847	4.318
HETATM	121	H	4.150	5.600	3.731
HETATM	122	H	-3.509	4.202	-2.750
HETATM	123	H	-4.184	5.147	-1.385
HETATM	124	H	-4.937	2.263	-2.095
HETATM	125	H	-5.939	3.700	-2.416
HETATM	126	H	-5.593	3.183	-0.747
HETATM	127	H	6.885	-2.977	4.261

```
HETATM  128  H              7.475  -2.992   2.586
HETATM  129  H              7.674  -4.413   3.610
HETATM  130  H              4.819  -4.395   4.908
HETATM  131  H              5.772  -5.774   4.338
HETATM  132  H              4.135  -5.528   3.737
HETATM  133  H              6.632  -5.807   1.883
HETATM  134  H              6.277  -4.440   0.815
HETATM  135  H              4.988  -5.545   1.307
HETATM  136  H              3.714  -5.146  -1.724
HETATM  137  H              2.924  -4.200  -3.026
HETATM  138  H              5.374  -3.700  -2.900
HETATM  139  H              4.403  -2.263  -2.496
HETATM  140  H              5.172  -3.182  -1.209
CONECT    1   10   12   73
CONECT    2   35   48
CONECT    3   11   14   74
CONECT    4   13   28   75
CONECT    5   35
CONECT    6   18   19   76
CONECT    7   44   49
CONECT    8   47   64
CONECT    9   44
CONECT   10    1   20   31
CONECT   11    3   16   17
CONECT   12    1   15   39
CONECT   13    4   17   27
CONECT   14    3   20   23
CONECT   15   12   18   29
CONECT   16   11   22   23
CONECT   17   11   13   21
CONECT   18    6   15   38
CONECT   19    6   26   30
CONECT   20   10   14   24
CONECT   21   17   25   77
CONECT   22   16   25   78
CONECT   23   14   16   32
CONECT   24   20   33   79
CONECT   25   21   22   56
CONECT   26   19   38   41
CONECT   27   13   35   42
CONECT   28    4   30   42
CONECT   29   15   36   80
CONECT   30   19   28   40
CONECT   31   10   39   47
```

```
CONECT   32   23   33   81
CONECT   33   24   32   45
CONECT   34   40   41   46
CONECT   35    2    5   27
CONECT   36   29   43   55
CONECT   37   47
CONECT   38   18   26   43
CONECT   39   12   31   44
CONECT   40   30   34   82
CONECT   41   26   34   83
CONECT   42   27   28   57
CONECT   43   36   38   84
CONECT   44    7    9   39
CONECT   45   33   51   52   54
CONECT   46   34   66   67   68
CONECT   47    8   31   37
CONECT   48    2   53   85   86
CONECT   49    7   50   87   88
CONECT   50   49   89   90   91
CONECT   51   45   92   93   94
CONECT   52   45   95   96   97
CONECT   53   48   98   99  100
CONECT   54   45  101  102  103
CONECT   55   36   61   62   63
CONECT   56   25   58   59   60
CONECT   57   42   69   72
CONECT   58   56  104  105  106
CONECT   59   56  107  108  109
CONECT   60   56  110  111  112
CONECT   61   55  113  114  115
CONECT   62   55  116  117  118
CONECT   63   55  119  120  121
CONECT   64    8   65  122  123
CONECT   65   64  124  125  126
CONECT   66   46  127  128  129
CONECT   67   46  130  131  132
CONECT   68   46  133  134  135
CONECT   69   57   70
CONECT   70   69   71  136  137
CONECT   71   70  138  139  140
CONECT   72   57
CONECT   73    1
CONECT   74    3
CONECT   75    4
```

CONECT 76 6
CONECT 77 21
CONECT 78 22
CONECT 79 24
CONECT 80 29
CONECT 81 32
CONECT 82 40
CONECT 83 41
CONECT 84 43
CONECT 85 48
CONECT 86 48
CONECT 87 49
CONECT 88 49
CONECT 89 50
CONECT 90 50
CONECT 91 50
CONECT 92 51
CONECT 93 51
CONECT 94 51
CONECT 95 52
CONECT 96 52
CONECT 97 52
CONECT 98 53
CONECT 99 53
CONECT 100 53
CONECT 101 54
CONECT 102 54
CONECT 103 54
CONECT 104 58
CONECT 105 58
CONECT 106 58
CONECT 107 59
CONECT 108 59
CONECT 109 59
CONECT 110 60
CONECT 111 60
CONECT 112 60
CONECT 113 61
CONECT 114 61
CONECT 115 61
CONECT 116 62
CONECT 117 62
CONECT 118 62
CONECT 119 63

```
CONECT  120   63
CONECT  121   63
CONECT  122   64
CONECT  123   64
CONECT  124   65
CONECT  125   65
CONECT  126   65
CONECT  127   66
CONECT  128   66
CONECT  129   66
CONECT  130   67
CONECT  131   67
CONECT  132   67
CONECT  133   68
CONECT  134   68
CONECT  135   68
CONECT  136   70
CONECT  137   70
CONECT  138   71
CONECT  139   71
CONECT  140   71
END
```

```
HEADER     MMFF94 OPTIMIZED HOST
COMPND   USING COORDINATES FROM XRAY STRUCTURE
AUTHOR    GENERATED BY PCMODEL V 9.30
HETATM    1  N      0.078   2.505   0.800
HETATM    2  O     -1.158  -4.032  -3.278
HETATM    3  N     -2.096  -0.023   0.333
HETATM    4  N      0.000  -2.546   0.776
HETATM    5  O     -2.671  -4.800  -1.740
HETATM    6  N      2.167  -0.023   0.333
HETATM    7  O      1.216   3.981  -3.268
HETATM    8  O     -2.647   3.515  -2.236
HETATM    9  O      2.741   4.742  -1.737
HETATM   10  C     -1.052   2.836   0.093
HETATM   11  C     -2.791  -1.148   0.705
HETATM   12  C      1.213   2.870   0.118
HETATM   13  C     -1.139  -2.915   0.102
HETATM   14  C     -2.753   1.124   0.708
HETATM   15  C      2.528   2.456   0.596
HETATM   16  C     -4.002  -0.709   1.230
HETATM   17  C     -2.451  -2.503   0.588
HETATM   18  C      2.868   1.100   0.702
HETATM   19  C      2.830  -1.172   0.687
HETATM   20  C     -2.371   2.467   0.594
HETATM   21  C     -3.417  -3.434   1.000
HETATM   22  C     -4.948  -1.678   1.639
HETATM   23  C     -3.977   0.725   1.235
HETATM   24  C     -3.297   3.436   1.019
HETATM   25  C     -4.672  -3.056   1.520
HETATM   26  C      4.062  -0.777   1.198
HETATM   27  C     -0.735  -3.638  -1.014
HETATM   28  C      1.127  -2.885   0.069
HETATM   29  C      3.497   3.385   1.005
HETATM   30  C      2.448  -2.514   0.563
HETATM   31  C     -0.640   3.570  -1.015
HETATM   32  C     -4.881   1.726   1.651
HETATM   33  C     -4.557   3.100   1.542
HETATM   34  C      4.660  -3.147   1.462
HETATM   35  C     -1.622  -4.230  -2.011
HETATM   36  C      4.757   3.003   1.508
HETATM   37  O     -1.206   5.267  -2.556
HETATM   38  C      4.085   0.658   1.209
HETATM   39  C      0.805   3.585  -1.002
```

HETATM	40	C	3.386	-3.481	0.959
HETATM	41	C	4.981	-1.779	1.585
HETATM	42	C	0.710	-3.633	-1.028
HETATM	43	C	5.035	1.624	1.613
HETATM	44	C	1.688	4.175	-2.003
HETATM	45	C	-5.572	4.161	2.008
HETATM	46	C	5.645	-4.264	1.856
HETATM	47	C	-1.500	4.214	-2.004
HETATM	48	C	-1.958	-4.633	-4.300
HETATM	49	C	2.015	4.580	-4.292
HETATM	50	C	1.329	4.343	-5.623
HETATM	51	C	-5.836	3.991	3.519
HETATM	52	C	-5.104	5.617	1.785
HETATM	53	C	-1.273	-4.400	-5.632
HETATM	54	C	-6.900	3.990	1.239
HETATM	55	C	5.776	4.083	1.920
HETATM	56	C	-5.686	-4.138	1.936
HETATM	57	C	1.564	-4.298	-2.010
HETATM	58	C	-7.020	-3.573	2.473
HETATM	59	C	-5.080	-5.016	3.052
HETATM	60	C	-6.026	-5.026	0.719
HETATM	61	C	7.114	3.513	2.442
HETATM	62	C	6.106	4.976	0.704
HETATM	63	C	5.181	4.955	3.046
HETATM	64	C	-3.552	4.085	-3.189
HETATM	65	C	-4.489	5.058	-2.498
HETATM	66	C	5.939	-5.151	0.627
HETATM	67	C	7.004	-3.744	2.378
HETATM	68	C	5.027	-5.131	2.975
HETATM	69	O	2.737	-3.636	-2.221
HETATM	70	C	3.640	-4.235	-3.158
HETATM	71	C	3.307	-3.782	-4.567
HETATM	72	O	1.244	-5.336	-2.575
HETATM	73	H	0.076	2.028	1.693
HETATM	74	H	-1.221	-0.036	-0.168
HETATM	75	H	0.005	-2.062	1.666
HETATM	76	H	1.285	-0.005	-0.156
HETATM	77	H	-3.173	-4.492	0.907
HETATM	78	H	-5.890	-1.321	2.040
HETATM	79	H	-2.998	4.479	0.933
HETATM	80	H	3.253	4.443	0.918
HETATM	81	H	-5.837	1.414	2.063
HETATM	82	H	3.104	-4.529	0.868
HETATM	83	H	5.940	-1.455	1.975

HETATM	84	H	5.982	1.264	2.000
HETATM	85	H	-2.952	-4.171	-4.308
HETATM	86	H	-2.052	-5.710	-4.116
HETATM	87	H	2.108	5.657	-4.112
HETATM	88	H	3.010	4.119	-4.300
HETATM	89	H	0.324	4.778	-5.621
HETATM	90	H	1.903	4.784	-6.443
HETATM	91	H	1.211	3.270	-5.810
HETATM	92	H	-4.907	4.082	4.095
HETATM	93	H	-6.532	4.753	3.887
HETATM	94	H	-6.274	3.015	3.756
HETATM	95	H	-4.188	5.837	2.346
HETATM	96	H	-4.913	5.821	0.725
HETATM	97	H	-5.864	6.332	2.120
HETATM	98	H	-1.150	-3.330	-5.821
HETATM	99	H	-0.270	-4.841	-5.630
HETATM	100	H	-1.850	-4.841	-6.450
HETATM	101	H	-7.373	3.021	1.435
HETATM	102	H	-7.625	4.761	1.525
HETATM	103	H	-6.743	4.065	0.157
HETATM	104	H	-6.866	-2.959	3.369
HETATM	105	H	-7.530	-2.960	1.721
HETATM	106	H	-7.708	-4.380	2.751
HETATM	107	H	-4.798	-4.409	3.921
HETATM	108	H	-5.796	-5.773	3.392
HETATM	109	H	-4.183	-5.550	2.720
HETATM	110	H	-6.428	-4.427	-0.107
HETATM	111	H	-5.150	-5.563	0.338
HETATM	112	H	-6.776	-5.782	0.979
HETATM	113	H	7.618	2.905	1.683
HETATM	114	H	7.805	4.319	2.719
HETATM	115	H	6.968	2.894	3.335
HETATM	116	H	6.500	4.380	-0.128
HETATM	117	H	5.227	5.515	0.334
HETATM	118	H	6.858	5.730	0.960
HETATM	119	H	4.907	4.345	3.915
HETATM	120	H	5.900	5.710	3.383
HETATM	121	H	4.282	5.492	2.725
HETATM	122	H	-4.129	3.259	-3.617
HETATM	123	H	-3.015	4.564	-4.016
HETATM	124	H	-5.031	4.557	-1.690
HETATM	125	H	-5.214	5.468	-3.207
HETATM	126	H	-3.934	5.885	-2.045
HETATM	127	H	6.348	-4.558	-0.200

```
HETATM 128  H            5.040  -5.656   0.254
HETATM 129  H            6.668  -5.933   0.869
HETATM 130  H            7.522  -3.142   1.623
HETATM 131  H            7.670  -4.575   2.640
HETATM 132  H            6.883  -3.133   3.280
HETATM 133  H            4.110  -5.634   2.651
HETATM 134  H            4.777  -4.523   3.852
HETATM 135  H            5.724  -5.913   3.300
HETATM 136  H            3.635  -5.328  -3.075
HETATM 137  H            4.647  -3.898  -2.891
HETATM 138  H            2.303  -4.107  -4.858
HETATM 139  H            4.026  -4.187  -5.285
HETATM 140  H            3.320  -2.689  -4.633
CONECT    1   10   12   73
CONECT    2   35   48
CONECT    3   11   14   74
CONECT    4   13   28   75
CONECT    5   35
CONECT    6   18   19   76
CONECT    7   44   49
CONECT    8   47   64
CONECT    9   44
CONECT   10    1   20   31
CONECT   11    3   16   17
CONECT   12    1   15   39
CONECT   13    4   17   27
CONECT   14    3   20   23
CONECT   15   12   18   29
CONECT   16   11   22   23
CONECT   17   11   13   21
CONECT   18    6   15   38
CONECT   19    6   26   30
CONECT   20   10   14   24
CONECT   21   17   25   77
CONECT   22   16   25   78
CONECT   23   14   16   32
CONECT   24   20   33   79
CONECT   25   21   22   56
CONECT   26   19   38   41
CONECT   27   13   35   42
CONECT   28    4   30   42
CONECT   29   15   36   80
CONECT   30   19   28   40
CONECT   31   10   39   47
```

```
CONECT   32   23   33   81
CONECT   33   24   32   45
CONECT   34   40   41   46
CONECT   35    2    5   27
CONECT   36   29   43   55
CONECT   37   47
CONECT   38   18   26   43
CONECT   39   12   31   44
CONECT   40   30   34   82
CONECT   41   26   34   83
CONECT   42   27   28   57
CONECT   43   36   38   84
CONECT   44    7    9   39
CONECT   45   33   51   52   54
CONECT   46   34   66   67   68
CONECT   47    8   31   37
CONECT   48    2   53   85   86
CONECT   49    7   50   87   88
CONECT   50   49   89   90   91
CONECT   51   45   92   93   94
CONECT   52   45   95   96   97
CONECT   53   48   98   99  100
CONECT   54   45  101  102  103
CONECT   55   36   61   62   63
CONECT   56   25   58   59   60
CONECT   57   42   69   72
CONECT   58   56  104  105  106
CONECT   59   56  107  108  109
CONECT   60   56  110  111  112
CONECT   61   55  113  114  115
CONECT   62   55  116  117  118
CONECT   63   55  119  120  121
CONECT   64    8   65  122  123
CONECT   65   64  124  125  126
CONECT   66   46  127  128  129
CONECT   67   46  130  131  132
CONECT   68   46  133  134  135
CONECT   69   57   70
CONECT   70   69   71  136  137
CONECT   71   70  138  139  140
CONECT   72   57
CONECT   73    1
CONECT   74    3
CONECT   75    4
```

CONECT	76	6
CONECT	77	21
CONECT	78	22
CONECT	79	24
CONECT	80	29
CONECT	81	32
CONECT	82	40
CONECT	83	41
CONECT	84	43
CONECT	85	48
CONECT	86	48
CONECT	87	49
CONECT	88	49
CONECT	89	50
CONECT	90	50
CONECT	91	50
CONECT	92	51
CONECT	93	51
CONECT	94	51
CONECT	95	52
CONECT	96	52
CONECT	97	52
CONECT	98	53
CONECT	99	53
CONECT	100	53
CONECT	101	54
CONECT	102	54
CONECT	103	54
CONECT	104	58
CONECT	105	58
CONECT	106	58
CONECT	107	59
CONECT	108	59
CONECT	109	59
CONECT	110	60
CONECT	111	60
CONECT	112	60
CONECT	113	61
CONECT	114	61
CONECT	115	61
CONECT	116	62
CONECT	117	62
CONECT	118	62
CONECT	119	63

```
CONECT  120   63
CONECT  121   63
CONECT  122   64
CONECT  123   64
CONECT  124   65
CONECT  125   65
CONECT  126   65
CONECT  127   66
CONECT  128   66
CONECT  129   66
CONECT  130   67
CONECT  131   67
CONECT  132   67
CONECT  133   68
CONECT  134   68
CONECT  135   68
CONECT  136   70
CONECT  137   70
CONECT  138   71
CONECT  139   71
CONECT  140   71
END
```

HEADER MMFF94 OPTIMIZED HOST:FLOURIDE COMPLEX

COMPND LOWEST ENERGY PYRROLE-BOUND FORM BY SAMPLING IN VACUO MD RUN

AUTHOR GENERATED BY PCMODEL V 9.30

```
HETATM   1  N      -0.233   0.595  -3.011
HETATM   2  O      -1.851  -3.288   3.637
HETATM   3  N      -2.530   0.263  -0.697
HETATM   4  N      -0.575   0.567   1.850
HETATM   5  O      -3.264  -1.684   4.457
HETATM   6  N       1.725   0.283  -0.467
HETATM   7  O       1.081  -3.227  -4.840
HETATM   8  O      -2.771  -2.411  -4.503
HETATM   9  O       2.478  -1.600  -5.642
HETATM  10  C      -1.337  -0.049  -3.541
HETATM  11  C      -3.361   0.400   0.390
HETATM  12  C       0.945   0.014  -3.455
HETATM  13  C      -1.747  -0.031   2.288
HETATM  14  C      -3.200   0.394  -1.889
HETATM  15  C       2.265   0.395  -2.926
HETATM  16  C      -4.625   0.669  -0.126
HETATM  17  C      -3.071   0.342   1.763
HETATM  18  C       2.555   0.440  -1.552
HETATM  19  C       2.394   0.407   0.727
HETATM  20  C      -2.718   0.329  -3.205
HETATM  21  C      -4.153   0.609   2.623
HETATM  22  C      -5.683   0.905   0.780
HETATM  23  C      -4.523   0.664  -1.562
HETATM  24  C      -3.666   0.584  -4.217
HETATM  25  C      -5.461   0.887   2.170
HETATM  26  C       3.715   0.694   0.402
HETATM  27  C      -1.373  -1.053   3.157
HETATM  28  C       0.536  -0.072   2.373
HETATM  29  C       3.345   0.681  -3.783
HETATM  30  C       1.913   0.323   2.042
HETATM  31  C      -0.856  -1.050  -4.380
HETATM  32  C      -5.442   0.893  -2.612
HETATM  33  C      -5.024   0.865  -3.958
HETATM  34  C       4.214   0.874   2.800
HETATM  35  C      -2.266  -1.997   3.818
HETATM  36  C       4.650   0.967  -3.327
HETATM  37  O      -1.316  -2.398  -6.268
HETATM  38  C       3.816   0.716  -1.033
HETATM  39  C       0.582  -1.002  -4.336
```

HETATM	40	C	2.858	0.577	3.056
HETATM	41	C	4.631	0.921	1.455
HETATM	42	C	0.064	-1.087	3.200
HETATM	43	C	4.872	0.973	-1.937
HETATM	44	C	1.483	-1.931	-5.006
HETATM	45	C	-5.988	1.135	-5.126
HETATM	46	C	5.175	1.141	3.971
HETATM	47	C	-1.647	-2.000	-5.156
HETATM	48	C	-2.664	-4.262	4.294
HETATM	49	C	1.904	-4.186	-5.507
HETATM	50	C	1.306	-5.560	-5.276
HETATM	51	C	-6.026	-0.090	-6.066
HETATM	52	C	-7.443	1.415	-4.685
HETATM	53	C	-2.053	-5.627	4.048
HETATM	54	C	-5.507	2.369	-5.920
HETATM	55	C	5.768	1.259	-4.343
HETATM	56	C	-6.582	1.156	3.189
HETATM	57	C	0.865	-2.036	3.966
HETATM	58	C	-6.751	-0.068	4.115
HETATM	59	C	-7.960	1.431	2.543
HETATM	60	C	-6.224	2.393	4.041
HETATM	61	C	5.398	2.500	-5.181
HETATM	62	C	7.143	1.540	-3.694
HETATM	63	C	5.949	0.047	-5.283
HETATM	64	C	-3.582	-3.337	-5.227
HETATM	65	C	-4.760	-3.713	-4.349
HETATM	66	C	5.225	-0.094	4.897
HETATM	67	C	6.626	1.440	3.533
HETATM	68	C	4.681	2.361	4.779
HETATM	69	O	1.993	-2.429	3.310
HETATM	70	C	2.813	-3.355	4.023
HETATM	71	C	3.994	-3.711	3.141
HETATM	72	O	0.538	-2.451	5.074
HETATM	73	H	-0.281	1.347	-2.313
HETATM	74	H	-1.523	0.326	-0.628
HETATM	75	H	-0.534	1.327	1.160
HETATM	76	H	0.717	0.337	-0.536
HETATM	78	H	-3.956	0.596	3.694
HETATM	79	H	-6.659	1.120	0.361
HETATM	80	H	-3.315	0.561	-5.247
HETATM	81	H	3.148	0.678	-4.854
HETATM	82	H	-6.467	1.111	-2.337
HETATM	83	H	2.508	0.539	4.086
HETATM	84	H	5.653	1.152	1.182

HETATM	85	H	5.846	1.193	-1.516
HETATM	86	H	-3.680	-4.232	3.885
HETATM	87	H	-2.692	-4.060	5.371
HETATM	88	H	1.930	-3.972	-6.582
HETATM	89	H	2.920	-4.150	-5.098
HETATM	90	H	1.255	-5.783	-4.205
HETATM	91	H	0.281	-5.604	-5.659
HETATM	92	H	1.902	-6.333	-5.770
HETATM	93	H	-6.346	-0.990	-5.527
HETATM	94	H	-6.728	0.069	-6.893
HETATM	95	H	-5.049	-0.304	-6.512
HETATM	96	H	-7.868	0.566	-4.137
HETATM	97	H	-7.507	2.302	-4.045
HETATM	98	H	-8.090	1.596	-5.551
HETATM	99	H	-1.027	-5.665	4.431
HETATM	100	H	-2.641	-6.411	4.533
HETATM	101	H	-2.000	-5.838	2.975
HETATM	102	H	-4.520	2.214	-6.369
HETATM	103	H	-6.197	2.606	-6.738
HETATM	104	H	-5.439	3.252	-5.274
HETATM	105	H	-6.968	-0.974	3.537
HETATM	106	H	-5.854	-0.264	4.712
HETATM	107	H	-7.576	0.082	4.822
HETATM	108	H	-8.300	0.579	1.943
HETATM	109	H	-8.725	1.614	3.307
HETATM	110	H	-7.934	2.316	1.897
HETATM	111	H	-5.311	2.243	4.627
HETATM	112	H	-6.068	3.276	3.409
HETATM	113	H	-7.025	2.629	4.751
HETATM	114	H	4.486	2.349	-5.769
HETATM	115	H	5.233	3.375	-4.540
HETATM	116	H	6.196	2.753	-5.889
HETATM	117	H	7.906	1.739	-4.456
HETATM	118	H	7.108	2.418	-3.038
HETATM	119	H	7.493	0.685	-3.103
HETATM	120	H	5.054	-0.152	-5.881
HETATM	121	H	6.773	0.212	-5.987
HETATM	122	H	6.175	-0.863	-4.714
HETATM	123	H	-3.000	-4.233	-5.469
HETATM	124	H	-3.942	-2.869	-6.150
HETATM	125	H	-4.414	-4.177	-3.419
HETATM	126	H	-5.424	-4.411	-4.866
HETATM	127	H	-5.331	-2.823	-4.065
HETATM	128	H	5.554	-0.984	4.349

```
HETATM  129  H              4.250  -0.322   5.341
HETATM  130  H              5.925   0.063   5.727
HETATM  131  H              7.060   0.600   2.976
HETATM  132  H              7.272   1.618   4.400
HETATM  133  H              6.682   2.335   2.903
HETATM  134  H              5.369   2.596   5.600
HETATM  135  H              3.696   2.192   5.226
HETATM  136  H              4.605   3.250   4.142
HETATM  137  H              3.168  -2.894   4.951
HETATM  138  H              2.240  -4.260   4.256
HETATM  139  H              4.666  -4.408   3.650
HETATM  140  H              3.653  -4.167   2.207
HETATM  141  H              4.557  -2.812   2.867
HETATM   77  F             -0.411   2.056  -0.572
CONECT    1   10   12   73
CONECT    2   35   48
CONECT    3   11   14   74
CONECT    4   13   28   75
CONECT    5   35
CONECT    6   18   19   76
CONECT    7   44   49
CONECT    8   47   64
CONECT    9   44
CONECT   10    1   20   31
CONECT   11    3   16   17
CONECT   12    1   15   39
CONECT   13    4   17   27
CONECT   14    3   20   23
CONECT   15   12   18   29
CONECT   16   11   22   23
CONECT   17   11   13   21
CONECT   18    6   15   38
CONECT   19    6   26   30
CONECT   20   10   14   24
CONECT   21   17   25   78
CONECT   22   16   25   79
CONECT   23   14   16   32
CONECT   24   20   33   80
CONECT   25   21   22   56
CONECT   26   19   38   41
CONECT   27   13   35   42
CONECT   28    4   30   42
CONECT   29   15   36   81
CONECT   30   19   28   40
```

```
CONECT   31   10   39   47
CONECT   32   23   33   82
CONECT   33   24   32   45
CONECT   34   40   41   46
CONECT   35    2    5   27
CONECT   36   29   43   55
CONECT   37   47
CONECT   38   18   26   43
CONECT   39   12   31   44
CONECT   40   30   34   83
CONECT   41   26   34   84
CONECT   42   27   28   57
CONECT   43   36   38   85
CONECT   44    7    9   39
CONECT   45   33   51   52   54
CONECT   46   34   66   67   68
CONECT   47    8   31   37
CONECT   48    2   53   86   87
CONECT   49    7   50   88   89
CONECT   50   49   90   91   92
CONECT   51   45   93   94   95
CONECT   52   45   96   97   98
CONECT   53   48   99  100  101
CONECT   54   45  102  103  104
CONECT   55   36   61   62   63
CONECT   56   25   58   59   60
CONECT   57   42   69   72
CONECT   58   56  105  106  107
CONECT   59   56  108  109  110
CONECT   60   56  111  112  113
CONECT   61   55  114  115  116
CONECT   62   55  117  118  119
CONECT   63   55  120  121  122
CONECT   64    8   65  123  124
CONECT   65   64  125  126  127
CONECT   66   46  128  129  130
CONECT   67   46  131  132  133
CONECT   68   46  134  135  136
CONECT   69   57   70
CONECT   70   69   71  137  138
CONECT   71   70  139  140  141
CONECT   72   57
CONECT   73    1
CONECT   74    3
```

CONECT 75 4
CONECT 76 6
CONECT 78 21
CONECT 79 22
CONECT 80 24
CONECT 81 29
CONECT 82 32
CONECT 83 40
CONECT 84 41
CONECT 85 43
CONECT 86 48
CONECT 87 48
CONECT 88 49
CONECT 89 49
CONECT 90 50
CONECT 91 50
CONECT 92 50
CONECT 93 51
CONECT 94 51
CONECT 95 51
CONECT 96 52
CONECT 97 52
CONECT 98 52
CONECT 99 53
CONECT 100 53
CONECT 101 53
CONECT 102 54
CONECT 103 54
CONECT 104 54
CONECT 105 58
CONECT 106 58
CONECT 107 58
CONECT 108 59
CONECT 109 59
CONECT 110 59
CONECT 111 60
CONECT 112 60
CONECT 113 60
CONECT 114 61
CONECT 115 61
CONECT 116 61
CONECT 117 62
CONECT 118 62
CONECT 119 62

```
CONECT  120   63
CONECT  121   63
CONECT  122   63
CONECT  123   64
CONECT  124   64
CONECT  125   65
CONECT  126   65
CONECT  127   65
CONECT  128   66
CONECT  129   66
CONECT  130   66
CONECT  131   67
CONECT  132   67
CONECT  133   67
CONECT  134   68
CONECT  135   68
CONECT  136   68
CONECT  137   70
CONECT  138   70
CONECT  139   71
CONECT  140   71
CONECT  141   71
END
```

HEADER MMFF94 OPTIMIZED HOST:FLOURIDE COMPLEX

COMPND LOWEST ENERGY CARBZOLE-BOUND FORM BY SAMPLING IN VACUO MD RUN

AUTHOR GENERATED BY PCMODEL V 9.30

```
HETATM    1  N        2.846   1.134   1.035
HETATM    2  O       -4.100  -2.397  -0.868
HETATM    3  N        0.702   0.741  -1.400
HETATM    4  N       -1.862   1.170   0.522
HETATM    5  O       -4.468  -0.724  -2.385
HETATM    6  N        0.276   0.709   2.949
HETATM    7  O        5.038  -2.484   2.367
HETATM    8  O        4.849  -1.484  -1.547
HETATM    9  O        5.425  -0.841   3.911
HETATM   10  C        3.511   0.610  -0.048
HETATM   11  C       -0.356   1.200  -2.161
HETATM   12  C        3.312   0.628   2.226
HETATM   13  C       -2.335   0.690  -0.677
HETATM   14  C        1.910   1.174  -1.915
HETATM   15  C        2.729   1.071   3.494
HETATM   16  C        0.202   1.749  -3.313
HETATM   17  C       -1.745   1.145  -1.938
HETATM   18  C        1.340   1.143   3.718
HETATM   19  C       -0.926   1.151   3.472
HETATM   20  C        3.215   1.084  -1.400
HETATM   21  C       -2.561   1.642  -2.973
HETATM   22  C       -0.660   2.221  -4.330
HETATM   23  C        1.627   1.730  -3.159
HETATM   24  C        4.251   1.552  -2.234
HETATM   25  C       -2.057   2.165  -4.179
HETATM   26  C       -0.636   1.683   4.724
HETATM   27  C       -3.309  -0.254  -0.364
HETATM   28  C       -2.534   0.638   1.596
HETATM   29  C        3.551   1.541   4.537
HETATM   30  C       -2.232   1.087   2.956
HETATM   31  C        4.410  -0.325   0.456
HETATM   32  C        2.700   2.176  -3.965
HETATM   33  C        4.032   2.085  -3.519
HETATM   34  C       -3.036   2.064   5.090
HETATM   35  C       -4.010  -1.109  -1.313
HETATM   36  C        3.054   2.051   5.752
HETATM   37  O        6.433  -1.497   0.100
HETATM   38  C        0.790   1.679   4.879
HETATM   39  C        4.274  -0.323   1.897
```

HETATM	40	C	-3.262	1.556	3.797
HETATM	41	C	-1.704	2.131	5.538
HETATM	42	C	-3.445	-0.277	1.078
HETATM	43	C	1.658	2.124	5.903
HETATM	44	C	4.963	-1.202	2.833
HETATM	45	C	5.224	2.556	-4.369
HETATM	46	C	-4.222	2.538	5.947
HETATM	47	C	5.330	-1.153	-0.316
HETATM	48	C	-4.781	-3.283	-1.756
HETATM	49	C	5.706	-3.394	3.241
HETATM	50	C	5.735	-4.754	2.572
HETATM	51	C	4.825	3.095	-5.761
HETATM	52	C	5.969	3.692	-3.635
HETATM	53	C	-4.826	-4.654	-1.110
HETATM	54	C	6.196	1.378	-4.600
HETATM	55	C	4.031	2.519	6.844
HETATM	56	C	-3.028	2.663	-5.264
HETATM	57	C	-4.376	-1.105	1.836
HETATM	58	C	-2.325	3.173	-6.543
HETATM	59	C	-3.871	3.831	-4.709
HETATM	60	C	-3.968	1.515	-5.691
HETATM	61	C	4.956	1.351	7.253
HETATM	62	C	4.890	3.684	6.308
HETATM	63	C	3.335	3.018	8.131
HETATM	64	C	5.712	-2.307	-2.331
HETATM	65	C	5.010	-2.613	-3.639
HETATM	66	C	-4.950	3.696	5.232
HETATM	67	C	-5.209	1.370	6.161
HETATM	68	C	-3.815	3.050	7.348
HETATM	69	O	-3.900	-1.461	3.063
HETATM	70	C	-4.774	-2.286	3.833
HETATM	71	C	-4.076	-2.621	5.136
HETATM	72	O	-5.484	-1.428	1.416
HETATM	73	H	2.084	1.791	0.962
HETATM	74	H	0.608	0.139	-0.571
HETATM	75	H	-1.091	1.816	0.605
HETATM	76	H	0.363	0.119	2.111
HETATM	78	H	-3.639	1.622	-2.814
HETATM	79	H	-0.200	2.634	-5.220
HETATM	80	H	5.267	1.500	-1.845
HETATM	81	H	4.628	1.508	4.377
HETATM	82	H	2.453	2.598	-4.932
HETATM	83	H	-4.278	1.524	3.408
HETATM	84	H	-1.450	2.534	6.511

HETATM	85	H	1.204	2.528	6.800
HETATM	86	H	-4.242	-3.341	-2.708
HETATM	87	H	-5.802	-2.926	-1.931
HETATM	88	H	6.731	-3.052	3.422
HETATM	89	H	5.165	-3.460	4.191
HETATM	90	H	6.240	-5.489	3.204
HETATM	91	H	4.718	-5.104	2.365
HETATM	92	H	6.254	-4.700	1.609
HETATM	93	H	4.167	3.969	-5.682
HETATM	94	H	5.707	3.408	-6.332
HETATM	95	H	4.309	2.332	-6.357
HETATM	96	H	5.297	4.533	-3.427
HETATM	97	H	6.389	3.363	-2.679
HETATM	98	H	6.803	4.071	-4.237
HETATM	99	H	-3.813	-5.020	-0.911
HETATM	100	H	-5.344	-4.609	-0.146
HETATM	101	H	-5.341	-5.373	-1.754
HETATM	102	H	5.691	0.544	-5.103
HETATM	103	H	7.042	1.681	-5.228
HETATM	104	H	6.615	0.993	-3.665
HETATM	105	H	-1.672	4.028	-6.332
HETATM	106	H	-1.720	2.388	-7.011
HETATM	107	H	-3.056	3.505	-7.290
HETATM	108	H	-3.230	4.654	-4.370
HETATM	109	H	-4.548	4.229	-5.474
HETATM	110	H	-4.491	3.528	-3.859
HETATM	111	H	-3.398	0.653	-6.056
HETATM	112	H	-4.600	1.166	-4.866
HETATM	113	H	-4.642	1.834	-6.495
HETATM	114	H	5.582	1.006	6.423
HETATM	115	H	5.633	1.648	8.061
HETATM	116	H	4.373	0.491	7.604
HETATM	117	H	5.507	3.385	5.453
HETATM	118	H	4.261	4.521	5.982
HETATM	119	H	5.572	4.060	7.079
HETATM	120	H	2.719	2.233	8.586
HETATM	121	H	4.071	3.327	8.883
HETATM	122	H	2.694	3.884	7.934
HETATM	123	H	5.924	-3.241	-1.797
HETATM	124	H	6.649	-1.775	-2.530
HETATM	125	H	4.065	-3.136	-3.456
HETATM	126	H	5.638	-3.235	-4.283
HETATM	127	H	4.763	-1.688	-4.170
HETATM	128	H	-5.375	3.387	4.270

```
HETATM  129  H             -4.267   4.531   5.037
HETATM  130  H             -5.779   4.077   5.840
HETATM  131  H             -5.633   1.005   5.219
HETATM  132  H             -6.051   1.675   6.793
HETATM  133  H             -4.716   0.522   6.650
HETATM  134  H             -3.145   3.916   7.283
HETATM  135  H             -3.310   2.271   7.931
HETATM  136  H             -4.693   3.366   7.924
HETATM  137  H             -4.997  -3.208   3.285
HETATM  138  H             -5.704  -1.744   4.040
HETATM  139  H             -3.138  -3.153   4.945
HETATM  140  H             -4.712  -3.245   5.771
HETATM  141  H             -3.817  -1.708   5.682
HETATM   77  F              0.477  -0.990   0.761
CONECT    1   10   12   73
CONECT    2   35   48
CONECT    3   11   14   74
CONECT    4   13   28   75
CONECT    5   35
CONECT    6   18   19   76
CONECT    7   44   49
CONECT    8   47   64
CONECT    9   44
CONECT   10    1   20   31
CONECT   11    3   16   17
CONECT   12    1   15   39
CONECT   13    4   17   27
CONECT   14    3   20   23
CONECT   15   12   18   29
CONECT   16   11   22   23
CONECT   17   11   13   21
CONECT   18    6   15   38
CONECT   19    6   26   30
CONECT   20   10   14   24
CONECT   21   17   25   78
CONECT   22   16   25   79
CONECT   23   14   16   32
CONECT   24   20   33   80
CONECT   25   21   22   56
CONECT   26   19   38   41
CONECT   27   13   35   42
CONECT   28    4   30   42
CONECT   29   15   36   81
CONECT   30   19   28   40
```

```
CONECT   31   10   39   47
CONECT   32   23   33   82
CONECT   33   24   32   45
CONECT   34   40   41   46
CONECT   35    2    5   27
CONECT   36   29   43   55
CONECT   37   47
CONECT   38   18   26   43
CONECT   39   12   31   44
CONECT   40   30   34   83
CONECT   41   26   34   84
CONECT   42   27   28   57
CONECT   43   36   38   85
CONECT   44    7    9   39
CONECT   45   33   51   52   54
CONECT   46   34   66   67   68
CONECT   47    8   31   37
CONECT   48    2   53   86   87
CONECT   49    7   50   88   89
CONECT   50   49   90   91   92
CONECT   51   45   93   94   95
CONECT   52   45   96   97   98
CONECT   53   48   99  100  101
CONECT   54   45  102  103  104
CONECT   55   36   61   62   63
CONECT   56   25   58   59   60
CONECT   57   42   69   72
CONECT   58   56  105  106  107
CONECT   59   56  108  109  110
CONECT   60   56  111  112  113
CONECT   61   55  114  115  116
CONECT   62   55  117  118  119
CONECT   63   55  120  121  122
CONECT   64    8   65  123  124
CONECT   65   64  125  126  127
CONECT   66   46  128  129  130
CONECT   67   46  131  132  133
CONECT   68   46  134  135  136
CONECT   69   57   70
CONECT   70   69   71  137  138
CONECT   71   70  139  140  141
CONECT   72   57
CONECT   73    1
CONECT   74    3
```

CONECT 75 4
CONECT 76 6
CONECT 78 21
CONECT 79 22
CONECT 80 24
CONECT 81 29
CONECT 82 32
CONECT 83 40
CONECT 84 41
CONECT 85 43
CONECT 86 48
CONECT 87 48
CONECT 88 49
CONECT 89 49
CONECT 90 50
CONECT 91 50
CONECT 92 50
CONECT 93 51
CONECT 94 51
CONECT 95 51
CONECT 96 52
CONECT 97 52
CONECT 98 52
CONECT 99 53
CONECT 100 53
CONECT 101 53
CONECT 102 54
CONECT 103 54
CONECT 104 54
CONECT 105 58
CONECT 106 58
CONECT 107 58
CONECT 108 59
CONECT 109 59
CONECT 110 59
CONECT 111 60
CONECT 112 60
CONECT 113 60
CONECT 114 61
CONECT 115 61
CONECT 116 61
CONECT 117 62
CONECT 118 62
CONECT 119 62

```
CONECT  120   63
CONECT  121   63
CONECT  122   63
CONECT  123   64
CONECT  124   64
CONECT  125   65
CONECT  126   65
CONECT  127   65
CONECT  128   66
CONECT  129   66
CONECT  130   66
CONECT  131   67
CONECT  132   67
CONECT  133   67
CONECT  134   68
CONECT  135   68
CONECT  136   68
CONECT  137   70
CONECT  138   70
CONECT  139   71
CONECT  140   71
CONECT  141   71
END
```

```
HEADER    MMFF94 OPTIMIZED HOST:CHLORIDE COMPLEX

COMPND   LOWEST ENERGY PYRROLE-BOUND FORM BY SAMPLING IN VACUO MD RUN

AUTHOR    GENERATED BY PCMODEL V 9.30

HETATM    1  N        0.082   2.064  -2.428
HETATM    2  O       -1.629  -1.920   4.071
HETATM    3  N       -2.265   1.632  -0.124
HETATM    4  N       -0.356   2.041   2.482
HETATM    5  O       -3.106  -0.356   4.856
HETATM    6  N        1.993   1.646   0.174
HETATM    7  O        1.382  -1.874  -4.053
HETATM    8  O       -2.507  -0.937  -3.846
HETATM    9  O        2.848  -0.293  -4.823
HETATM   10  C       -1.017   1.388  -2.924
HETATM   11  C       -3.094   1.865   0.946
HETATM   12  C        1.254   1.431  -2.800
HETATM   13  C       -1.524   1.396   2.849
HETATM   14  C       -2.891   1.869  -1.322
HETATM   15  C        2.558   1.839  -2.271
HETATM   16  C       -4.328   2.224   0.414
HETATM   17  C       -2.831   1.800   2.323
HETATM   18  C        2.820   1.894  -0.894
HETATM   19  C        2.617   1.877   1.374
HETATM   20  C       -2.389   1.808  -2.629
HETATM   21  C       -3.905   2.116   3.172
HETATM   22  C       -5.382   2.522   1.306
HETATM   23  C       -4.200   2.227  -1.018
HETATM   24  C       -3.293   2.131  -3.658
HETATM   25  C       -5.187   2.472   2.700
HETATM   26  C        3.923   2.247   1.073
HETATM   27  C       -1.163   0.332   3.670
HETATM   28  C        0.747   1.368   2.972
HETATM   29  C        3.630   2.171  -3.118
HETATM   30  C        2.116   1.800   2.680
HETATM   31  C       -0.537   0.341  -3.706
HETATM   32  C       -5.078   2.531  -2.084
HETATM   33  C       -4.636   2.488  -3.422
HETATM   34  C        4.358   2.489   3.480
HETATM   35  C       -2.070  -0.641   4.265
HETATM   36  C        4.909   2.531  -2.643
HETATM   37  O       -0.968  -1.104  -5.529
HETATM   38  C        4.051   2.257  -0.358
HETATM   39  C        0.900   0.372  -3.632
```

HETATM	40	C	3.018	2.120	3.713
HETATM	41	C	4.800	2.547	2.142
HETATM	42	C	0.274	0.311	3.745
HETATM	43	C	5.104	2.571	-1.248
HETATM	44	C	1.814	-0.590	-4.235
HETATM	45	C	-5.558	2.827	-4.606
HETATM	46	C	5.278	2.824	4.667
HETATM	47	C	-1.332	-0.622	-4.461
HETATM	48	C	-2.457	-2.922	4.666
HETATM	49	C	2.217	-2.864	-4.656
HETATM	50	C	1.584	-4.221	-4.420
HETATM	51	C	-7.003	3.187	-4.190
HETATM	52	C	-4.990	4.039	-5.376
HETATM	53	C	-1.814	-4.272	4.418
HETATM	54	C	-5.649	1.615	-5.559
HETATM	55	C	6.027	2.874	-3.643
HETATM	56	C	-6.307	2.798	3.704
HETATM	57	C	1.076	-0.653	4.491
HETATM	58	C	-6.557	1.579	4.619
HETATM	59	C	-7.657	3.151	3.039
HETATM	60	C	-5.894	4.008	4.569
HETATM	61	C	7.374	3.230	-2.975
HETATM	62	C	6.285	1.665	-4.569
HETATM	63	C	5.606	4.089	-4.498
HETATM	64	C	-3.336	-1.852	-4.564
HETATM	65	C	-4.584	-2.100	-3.739
HETATM	66	C	6.720	3.197	4.254
HETATM	67	C	4.700	4.025	5.447
HETATM	68	C	5.377	1.605	5.609
HETATM	69	O	2.253	-0.955	3.874
HETATM	70	C	3.089	-1.870	4.583
HETATM	71	C	4.338	-2.100	3.756
HETATM	72	O	0.716	-1.147	5.555
HETATM	73	H	0.032	2.888	-1.820
HETATM	74	H	-1.278	1.453	-0.037
HETATM	75	H	-0.313	2.871	1.881
HETATM	76	H	1.007	1.461	0.086
HETATM	78	H	-3.723	2.082	4.246
HETATM	79	H	-6.335	2.807	0.875
HETATM	80	H	-2.919	2.103	-4.680
HETATM	81	H	3.448	2.145	-4.191
HETATM	82	H	-6.091	2.816	-1.826
HETATM	83	H	2.643	2.080	4.734
HETATM	84	H	5.810	2.841	1.887

HETATM	85	H	6.054	2.858	-0.814
HETATM	86	H	-3.453	-2.897	4.210
HETATM	87	H	-2.540	-2.746	5.744
HETATM	88	H	2.300	-2.678	-5.733
HETATM	89	H	3.213	-2.836	-4.200
HETATM	90	H	1.478	-4.416	-3.347
HETATM	91	H	0.578	-4.257	-4.851
HETATM	92	H	2.188	-5.017	-4.865
HETATM	93	H	-7.029	4.071	-3.542
HETATM	94	H	-7.622	3.413	-5.067
HETATM	95	H	-7.488	2.359	-3.659
HETATM	96	H	-4.884	4.909	-4.718
HETATM	97	H	-4.005	3.831	-5.808
HETATM	98	H	-5.649	4.325	-6.204
HETATM	99	H	-0.808	-4.305	4.848
HETATM	100	H	-2.413	-5.076	4.856
HETATM	101	H	-1.707	-4.456	3.344
HETATM	102	H	-6.031	0.731	-5.037
HETATM	103	H	-6.323	1.823	-6.400
HETATM	104	H	-4.676	1.349	-5.988
HETATM	105	H	-6.817	0.691	4.031
HETATM	106	H	-5.680	1.327	5.225
HETATM	107	H	-7.382	1.771	5.316
HETATM	108	H	-8.033	2.324	2.425
HETATM	109	H	-8.424	3.367	3.793
HETATM	110	H	-7.574	4.039	2.402
HETATM	111	H	-5.000	3.804	5.167
HETATM	112	H	-5.681	4.884	3.945
HETATM	113	H	-6.692	4.283	5.269
HETATM	114	H	7.285	4.112	-2.330
HETATM	115	H	7.756	2.400	-2.369
HETATM	116	H	8.140	3.459	-3.727
HETATM	117	H	7.109	1.869	-5.264
HETATM	118	H	6.551	0.774	-3.989
HETATM	119	H	5.410	1.413	-5.178
HETATM	120	H	5.386	4.958	-3.866
HETATM	121	H	6.400	4.376	-5.195
HETATM	122	H	4.712	3.884	-5.098
HETATM	123	H	-2.802	-2.796	-4.720
HETATM	124	H	-3.612	-1.419	-5.532
HETATM	125	H	-4.323	-2.524	-2.764
HETATM	126	H	-5.262	-2.786	-4.253
HETATM	127	H	-5.111	-1.160	-3.544
HETATM	128	H	6.740	4.087	3.614

```
HETATM  129  H               7.210   2.378   3.715
HETATM  130  H               7.337   3.420   5.133
HETATM  131  H               4.589   4.900   4.797
HETATM  132  H               5.358   4.308   6.278
HETATM  133  H               3.718   3.806   5.877
HETATM  134  H               6.049   1.809   6.451
HETATM  135  H               5.765   0.727   5.079
HETATM  136  H               4.407   1.328   6.036
HETATM  137  H               3.362  -1.444   5.555
HETATM  138  H               2.562  -2.819   4.730
HETATM  139  H               5.021  -2.788   4.264
HETATM  140  H               4.080  -2.519   2.777
HETATM  141  H               4.859  -1.156   3.570
HETATM   77 Cl              -0.145   4.285   0.037
CONECT    1   10   12   73
CONECT    2   35   48
CONECT    3   11   14   74
CONECT    4   13   28   75
CONECT    5   35
CONECT    6   18   19   76
CONECT    7   44   49
CONECT    8   47   64
CONECT    9   44
CONECT   10    1   20   31
CONECT   11    3   16   17
CONECT   12    1   15   39
CONECT   13    4   17   27
CONECT   14    3   20   23
CONECT   15   12   18   29
CONECT   16   11   22   23
CONECT   17   11   13   21
CONECT   18    6   15   38
CONECT   19    6   26   30
CONECT   20   10   14   24
CONECT   21   17   25   78
CONECT   22   16   25   79
CONECT   23   14   16   32
CONECT   24   20   33   80
CONECT   25   21   22   56
CONECT   26   19   38   41
CONECT   27   13   35   42
CONECT   28    4   30   42
CONECT   29   15   36   81
CONECT   30   19   28   40
```

```
CONECT   31   10   39   47
CONECT   32   23   33   82
CONECT   33   24   32   45
CONECT   34   40   41   46
CONECT   35    2    5   27
CONECT   36   29   43   55
CONECT   37   47
CONECT   38   18   26   43
CONECT   39   12   31   44
CONECT   40   30   34   83
CONECT   41   26   34   84
CONECT   42   27   28   57
CONECT   43   36   38   85
CONECT   44    7    9   39
CONECT   45   33   51   52   54
CONECT   46   34   66   67   68
CONECT   47    8   31   37
CONECT   48    2   53   86   87
CONECT   49    7   50   88   89
CONECT   50   49   90   91   92
CONECT   51   45   93   94   95
CONECT   52   45   96   97   98
CONECT   53   48   99  100  101
CONECT   54   45  102  103  104
CONECT   55   36   61   62   63
CONECT   56   25   58   59   60
CONECT   57   42   69   72
CONECT   58   56  105  106  107
CONECT   59   56  108  109  110
CONECT   60   56  111  112  113
CONECT   61   55  114  115  116
CONECT   62   55  117  118  119
CONECT   63   55  120  121  122
CONECT   64    8   65  123  124
CONECT   65   64  125  126  127
CONECT   66   46  128  129  130
CONECT   67   46  131  132  133
CONECT   68   46  134  135  136
CONECT   69   57   70
CONECT   70   69   71  137  138
CONECT   71   70  139  140  141
CONECT   72   57
CONECT   73    1
CONECT   74    3
```

```
CONECT   75    4
CONECT   76    6
CONECT   78   21
CONECT   79   22
CONECT   80   24
CONECT   81   29
CONECT   82   32
CONECT   83   40
CONECT   84   41
CONECT   85   43
CONECT   86   48
CONECT   87   48
CONECT   88   49
CONECT   89   49
CONECT   90   50
CONECT   91   50
CONECT   92   50
CONECT   93   51
CONECT   94   51
CONECT   95   51
CONECT   96   52
CONECT   97   52
CONECT   98   52
CONECT   99   53
CONECT  100   53
CONECT  101   53
CONECT  102   54
CONECT  103   54
CONECT  104   54
CONECT  105   58
CONECT  106   58
CONECT  107   58
CONECT  108   59
CONECT  109   59
CONECT  110   59
CONECT  111   60
CONECT  112   60
CONECT  113   60
CONECT  114   61
CONECT  115   61
CONECT  116   61
CONECT  117   62
CONECT  118   62
CONECT  119   62
```

```
CONECT  120   63
CONECT  121   63
CONECT  122   63
CONECT  123   64
CONECT  124   64
CONECT  125   65
CONECT  126   65
CONECT  127   65
CONECT  128   66
CONECT  129   66
CONECT  130   66
CONECT  131   67
CONECT  132   67
CONECT  133   67
CONECT  134   68
CONECT  135   68
CONECT  136   68
CONECT  137   70
CONECT  138   70
CONECT  139   71
CONECT  140   71
CONECT  141   71
END
```

HEADER MMFF94 OPTIMIZED HOST:CHLORIDE COMPLEX

COMPND LOWEST ENERGY CARBAZOLE-BOUND FORM BY SAMPLING IN VACUO MD RUN

AUTHOR GENERATED BY PCMODEL V 9.30

HETATM	1	Cl	0.028	-2.350	0.000
HETATM	2	O	-4.604	-3.232	-0.477
HETATM	3	N	-0.324	0.076	-2.165
HETATM	4	N	-2.379	0.538	0.326
HETATM	5	O	-5.391	-1.625	-1.903
HETATM	6	N	0.323	0.075	2.165
HETATM	7	O	4.649	-3.168	0.469
HETATM	8	O	3.537	-2.138	-3.296
HETATM	9	O	5.413	-1.555	1.900
HETATM	10	C	2.709	-0.004	-1.524
HETATM	11	C	-1.540	0.526	-2.641
HETATM	12	C	3.069	0.022	0.723
HETATM	13	C	-3.070	-0.019	-0.723
HETATM	14	C	0.713	0.541	-2.949
HETATM	15	C	2.823	0.477	2.090
HETATM	16	C	-1.289	1.089	-3.889
HETATM	17	C	-2.830	0.443	-2.089
HETATM	18	C	1.532	0.541	2.642
HETATM	19	C	-0.721	0.523	2.951
HETATM	20	C	2.103	0.471	-2.766
HETATM	21	C	-3.886	0.915	-2.888
HETATM	22	C	-2.383	1.545	-4.661
HETATM	23	C	0.131	1.097	-4.084
HETATM	24	C	2.905	0.951	-3.819
HETATM	25	C	-3.700	1.452	-4.178
HETATM	26	C	-0.147	1.085	4.086
HETATM	27	C	-3.860	-1.031	-0.187
HETATM	28	C	-2.709	-0.045	1.524
HETATM	29	C	3.873	0.962	2.891
HETATM	30	C	-2.110	0.435	2.767
HETATM	31	C	3.657	-0.983	-1.246
HETATM	32	C	0.974	1.562	-5.121
HETATM	33	C	2.375	1.483	-5.010
HETATM	34	C	-2.395	1.437	5.014
HETATM	35	C	-4.692	-1.955	-0.949
HETATM	36	C	3.680	1.493	4.181
HETATM	37	O	5.466	-2.263	-2.076
HETATM	38	C	1.273	1.098	3.892
HETATM	39	C	3.874	-0.978	0.185

HETATM	40	C	-2.918	0.900	3.822
HETATM	41	C	-0.995	1.535	5.125
HETATM	42	C	-3.643	-1.037	1.244
HETATM	43	C	2.361	1.566	4.665
HETATM	44	C	4.719	-1.891	0.944
HETATM	45	C	3.321	1.964	-6.124
HETATM	46	C	-3.348	1.903	6.128
HETATM	47	C	4.315	-1.856	-2.213
HETATM	48	C	-5.377	-4.188	-1.200
HETATM	49	C	5.435	-4.116	1.191
HETATM	50	C	5.258	-5.472	0.535
HETATM	51	C	4.217	3.102	-5.589
HETATM	52	C	4.212	0.792	-6.591
HETATM	53	C	-5.181	-5.543	-0.548
HETATM	54	C	2.591	2.506	-7.373
HETATM	55	C	4.890	1.978	5.000
HETATM	56	C	-4.918	1.921	-4.994
HETATM	57	C	-4.289	-1.921	2.208
HETATM	58	C	-4.558	2.457	-6.400
HETATM	59	C	-5.638	3.061	-4.242
HETATM	60	C	-5.897	0.744	-5.194
HETATM	61	C	5.886	0.814	5.196
HETATM	62	C	5.595	3.129	4.250
HETATM	63	C	4.523	2.504	6.405
HETATM	64	C	4.131	-3.013	-4.253
HETATM	65	C	3.125	-3.244	-5.365
HETATM	66	C	-4.260	3.029	5.596
HETATM	67	C	-4.223	0.717	6.593
HETATM	68	C	-2.626	2.452	7.380
HETATM	69	O	-3.507	-2.195	3.291
HETATM	70	C	-4.088	-3.081	4.246
HETATM	71	C	-3.080	-3.300	5.357
HETATM	72	O	-5.434	-2.344	2.071
HETATM	73	N	2.371	0.572	-0.325
HETATM	74	H	-0.210	-0.556	-1.368
HETATM	75	H	-1.702	1.280	0.225
HETATM	76	H	0.217	-0.557	1.367
HETATM	77	H	-4.892	0.858	-2.474
HETATM	78	H	-2.163	1.969	-5.634
HETATM	79	H	3.985	0.904	-3.688
HETATM	80	H	4.880	0.920	2.476
HETATM	81	H	0.496	1.981	-6.000
HETATM	82	H	-3.997	0.840	3.690
HETATM	83	H	-0.523	1.959	6.004

HETATM	84	H	2.136	1.985	5.639
HETATM	85	H	-5.039	-4.227	-2.242
HETATM	86	H	-6.437	-3.913	-1.166
HETATM	87	H	6.491	-3.826	1.157
HETATM	88	H	5.098	-4.162	2.232
HETATM	89	H	5.844	-6.235	1.053
HETATM	90	H	4.204	-5.769	0.542
HETATM	91	H	5.571	-5.436	-0.514
HETATM	92	H	4.860	2.772	-4.766
HETATM	93	H	4.877	3.488	-6.375
HETATM	94	H	3.613	3.939	-5.219
HETATM	95	H	4.849	0.407	-5.787
HETATM	96	H	3.603	-0.044	-6.956
HETATM	97	H	4.877	1.100	-7.406
HETATM	98	H	-4.123	-5.825	-0.557
HETATM	99	H	-5.494	-5.514	0.500
HETATM	100	H	-5.757	-6.314	-1.068
HETATM	101	H	1.967	3.375	-7.132
HETATM	102	H	3.306	2.828	-8.140
HETATM	103	H	1.951	1.742	-7.830
HETATM	104	H	-3.898	3.330	-6.341
HETATM	105	H	-4.060	1.691	-7.006
HETATM	106	H	-5.456	2.768	-6.945
HETATM	107	H	-4.961	3.903	-4.058
HETATM	108	H	-6.490	3.438	-4.819
HETATM	109	H	-6.029	2.735	-3.271
HETATM	110	H	-5.405	-0.098	-5.695
HETATM	111	H	-6.300	0.373	-4.246
HETATM	112	H	-6.754	1.044	-5.809
HETATM	113	H	6.294	0.450	4.247
HETATM	114	H	6.739	1.124	5.812
HETATM	115	H	5.406	-0.036	5.695
HETATM	116	H	5.990	2.811	3.278
HETATM	117	H	4.906	3.962	4.068
HETATM	118	H	6.442	3.517	4.828
HETATM	119	H	4.036	1.731	7.010
HETATM	120	H	5.417	2.827	6.952
HETATM	121	H	3.851	3.369	6.350
HETATM	122	H	4.385	-3.968	-3.779
HETATM	123	H	5.036	-2.552	-4.665
HETATM	124	H	2.208	-3.691	-4.967
HETATM	125	H	3.537	-3.905	-6.132
HETATM	126	H	2.840	-2.295	-5.830
HETATM	127	H	-4.900	2.692	4.772

```
HETATM 128 H -3.668 3.875 5.229
HETATM 129 H -4.925 3.404 6.383
HETATM 130 H -4.854 0.325 5.788
HETATM 131 H -4.892 1.015 7.408
HETATM 132 H -3.602 -0.111 6.956
HETATM 133 H -2.014 3.329 7.140
HETATM 134 H -1.975 1.695 7.834
HETATM 135 H -3.345 2.761 8.147
HETATM 136 H -4.329 -4.038 3.770
HETATM 137 H -5.000 -2.634 4.659
HETATM 138 H -2.156 -3.733 4.958
HETATM 139 H -3.482 -3.969 6.123
HETATM 140 H -2.808 -2.349 5.824
HETATM 141 H 1.684 1.304 -0.222
CONECT 2 35 48
CONECT 3 11 14 74
CONECT 4 13 28 75
CONECT 5 35
CONECT 6 18 19 76
CONECT 7 44 49
CONECT 8 47 64
CONECT 9 44
CONECT 10 20 31 73
CONECT 11 3 16 17
CONECT 12 15 39 73
CONECT 13 4 17 27
CONECT 14 3 20 23
CONECT 15 12 18 29
CONECT 16 11 22 23
CONECT 17 11 13 21
CONECT 18 6 15 38
CONECT 19 6 26 30
CONECT 20 10 14 24
CONECT 21 17 25 77
CONECT 22 16 25 78
CONECT 23 14 16 32
CONECT 24 20 33 79
CONECT 25 21 22 56
CONECT 26 19 38 41
CONECT 27 13 35 42
CONECT 28 4 30 42
CONECT 29 15 36 80
CONECT 30 19 28 40
CONECT 31 10 39 47
```

```
CONECT   32   23   33   81
CONECT   33   24   32   45
CONECT   34   40   41   46
CONECT   35    2    5   27
CONECT   36   29   43   55
CONECT   37   47
CONECT   38   18   26   43
CONECT   39   12   31   44
CONECT   40   30   34   82
CONECT   41   26   34   83
CONECT   42   27   28   57
CONECT   43   36   38   84
CONECT   44    7    9   39
CONECT   45   33   51   52   54
CONECT   46   34   66   67   68
CONECT   47    8   31   37
CONECT   48    2   53   85   86
CONECT   49    7   50   87   88
CONECT   50   49   89   90   91
CONECT   51   45   92   93   94
CONECT   52   45   95   96   97
CONECT   53   48   98   99  100
CONECT   54   45  101  102  103
CONECT   55   36   61   62   63
CONECT   56   25   58   59   60
CONECT   57   42   69   72
CONECT   58   56  104  105  106
CONECT   59   56  107  108  109
CONECT   60   56  110  111  112
CONECT   61   55  113  114  115
CONECT   62   55  116  117  118
CONECT   63   55  119  120  121
CONECT   64    8   65  122  123
CONECT   65   64  124  125  126
CONECT   66   46  127  128  129
CONECT   67   46  130  131  132
CONECT   68   46  133  134  135
CONECT   69   57   70
CONECT   70   69   71  136  137
CONECT   71   70  138  139  140
CONECT   72   57
CONECT   73   10   12  141
CONECT   74    3
CONECT   75    4
```

```
CONECT   76    6
CONECT   77   21
CONECT   78   22
CONECT   79   24
CONECT   80   29
CONECT   81   32
CONECT   82   40
CONECT   83   41
CONECT   84   43
CONECT   85   48
CONECT   86   48
CONECT   87   49
CONECT   88   49
CONECT   89   50
CONECT   90   50
CONECT   91   50
CONECT   92   51
CONECT   93   51
CONECT   94   51
CONECT   95   52
CONECT   96   52
CONECT   97   52
CONECT   98   53
CONECT   99   53
CONECT  100   53
CONECT  101   54
CONECT  102   54
CONECT  103   54
CONECT  104   58
CONECT  105   58
CONECT  106   58
CONECT  107   59
CONECT  108   59
CONECT  109   59
CONECT  110   60
CONECT  111   60
CONECT  112   60
CONECT  113   61
CONECT  114   61
CONECT  115   61
CONECT  116   62
CONECT  117   62
CONECT  118   62
CONECT  119   63
```

```
CONECT  120   63
CONECT  121   63
CONECT  122   64
CONECT  123   64
CONECT  124   65
CONECT  125   65
CONECT  126   65
CONECT  127   66
CONECT  128   66
CONECT  129   66
CONECT  130   67
CONECT  131   67
CONECT  132   67
CONECT  133   68
CONECT  134   68
CONECT  135   68
CONECT  136   70
CONECT  137   70
CONECT  138   71
CONECT  139   71
CONECT  140   71
CONECT  141   73
END
```

HEADER MMFF94 OPTIMIZED HOST:HYDROGEN SULFATE COMPLEX

COMPND LOWEST ENERGY PYRROLE-BOUND FORM BY SAMPLING IN VACUO MD RUN

AUTHOR GENERATED BY PCMODEL V 9.30

HETATM	1	S	0.187	4.572	0.365
HETATM	2	O	0.093	3.402	-0.500
HETATM	3	O	-0.535	5.751	-0.070
HETATM	4	O	1.732	4.982	0.237
HETATM	5	O	0.096	4.235	1.779
HETATM	6	H	2.196	4.396	0.865
HETATM	7	O	1.687	-2.243	-4.083
HETATM	8	O	-2.220	-1.400	-3.914
HETATM	9	O	3.165	-0.630	-4.755
HETATM	10	C	-0.803	0.965	-2.988
HETATM	11	C	-2.853	1.431	0.886
HETATM	12	C	1.464	1.065	-2.822
HETATM	13	C	-1.295	0.854	2.743
HETATM	14	C	-2.663	1.460	-1.380
HETATM	15	C	2.736	1.543	-2.275
HETATM	16	C	-4.096	1.774	0.368
HETATM	17	C	-2.600	1.295	2.256
HETATM	18	C	2.973	1.639	-0.893
HETATM	19	C	2.767	1.608	1.376
HETATM	20	C	-2.180	1.366	-2.693
HETATM	21	C	-3.673	1.544	3.125
HETATM	22	C	-5.154	2.016	1.274
HETATM	23	C	-3.976	1.792	-1.065
HETATM	24	C	-3.102	1.648	-3.717
HETATM	25	C	-4.957	1.908	2.668
HETATM	26	C	4.040	2.080	1.076
HETATM	27	C	-0.896	-0.317	3.382
HETATM	28	C	0.956	0.948	2.969
HETATM	29	C	3.792	1.938	-3.115
HETATM	30	C	2.291	1.468	2.689
HETATM	31	C	-0.284	-0.075	-3.753
HETATM	32	C	-4.871	2.061	-2.126
HETATM	33	C	-4.446	1.997	-3.469
HETATM	34	C	4.472	2.316	3.484
HETATM	35	C	-1.758	-1.414	3.811
HETATM	36	C	5.033	2.400	-2.633
HETATM	37	O	-0.653	-1.538	-5.575
HETATM	38	C	4.171	2.097	-0.354
HETATM	39	C	1.154	-0.010	-3.649

HETATM	40	C	3.170	1.830	3.721
HETATM	41	C	4.894	2.437	2.145
HETATM	42	C	0.537	-0.255	3.525
HETATM	43	C	5.207	2.476	-1.237
HETATM	44	C	2.108	-0.950	-4.225
HETATM	45	C	-5.388	2.300	-4.648
HETATM	46	C	5.376	2.690	4.672
HETATM	47	C	-1.044	-1.060	-4.514
HETATM	48	C	-3.742	-2.617	3.409
HETATM	49	C	2.562	-3.212	-4.663
HETATM	50	C	1.936	-4.581	-4.479
HETATM	51	C	-4.839	3.494	-5.458
HETATM	52	C	-5.485	1.062	-5.567
HETATM	53	C	-4.885	-2.660	2.413
HETATM	54	C	-6.828	2.662	-4.219
HETATM	55	C	6.135	2.809	-3.626
HETATM	56	C	-6.083	2.173	3.682
HETATM	57	C	1.413	-1.290	4.058
HETATM	58	C	-6.335	0.900	4.521
HETATM	59	C	-7.430	2.565	3.033
HETATM	60	C	-5.677	3.329	4.621
HETATM	61	C	7.444	3.276	-2.949
HETATM	62	C	6.495	1.608	-4.528
HETATM	63	C	5.635	3.975	-4.506
HETATM	64	C	-3.014	-2.341	-4.636
HETATM	65	C	-4.260	-2.626	-3.820
HETATM	66	C	5.595	1.451	5.568
HETATM	67	C	6.772	3.205	4.257
HETATM	68	C	4.707	3.806	5.503
HETATM	69	O	1.048	-2.532	3.624
HETATM	70	C	1.854	-3.594	4.140
HETATM	71	C	1.297	-4.904	3.617
HETATM	72	O	2.374	-1.072	4.786
HETATM	73	N	0.271	1.672	-2.491
HETATM	74	O	-2.849	-1.576	3.010
HETATM	75	N	-2.012	1.275	-0.186
HETATM	76	N	-0.176	1.647	2.613
HETATM	77	O	-1.516	-2.117	4.787
HETATM	78	N	2.156	1.343	0.173
HETATM	79	H	-3.487	1.451	4.194
HETATM	80	H	-6.114	2.294	0.855
HETATM	81	H	-2.742	1.600	-4.743
HETATM	82	H	3.625	1.882	-4.190
HETATM	83	H	-5.888	2.329	-1.863

HETATM	84	H	2.814	1.719	4.744
HETATM	85	H	5.881	2.807	1.892
HETATM	86	H	6.131	2.834	-0.800
HETATM	87	H	-4.132	-2.406	4.411
HETATM	88	H	-3.216	-3.578	3.411
HETATM	89	H	2.691	-3.007	-5.732
HETATM	90	H	3.535	-3.179	-4.160
HETATM	91	H	0.952	-4.621	-4.957
HETATM	92	H	2.571	-5.361	-4.909
HETATM	93	H	1.783	-4.795	-3.416
HETATM	94	H	-3.860	3.280	-5.900
HETATM	95	H	-5.513	3.754	-6.283
HETATM	96	H	-4.728	4.382	-4.825
HETATM	97	H	-4.518	0.790	-6.002
HETATM	98	H	-5.856	0.190	-5.016
HETATM	99	H	-6.172	1.244	-6.402
HETATM	100	H	-5.402	-1.695	2.377
HETATM	101	H	-4.508	-2.855	1.403
HETATM	102	H	-5.605	-3.438	2.678
HETATM	103	H	-6.849	3.561	-3.592
HETATM	104	H	-7.461	2.865	-5.092
HETATM	105	H	-7.302	1.845	-3.663
HETATM	106	H	-6.606	0.054	3.881
HETATM	107	H	-5.455	0.605	5.102
HETATM	108	H	-7.154	1.054	5.234
HETATM	109	H	-7.806	1.773	2.375
HETATM	110	H	-8.200	2.742	3.795
HETATM	111	H	-7.342	3.486	2.445
HETATM	112	H	-4.787	3.090	5.213
HETATM	113	H	-5.458	4.241	4.052
HETATM	114	H	-6.479	3.563	5.331
HETATM	115	H	7.880	2.487	-2.325
HETATM	116	H	8.200	3.550	-3.696
HETATM	117	H	7.280	4.159	-2.320
HETATM	118	H	6.820	0.749	-3.930
HETATM	119	H	5.648	1.280	-5.141
HETATM	120	H	7.308	1.861	-5.218
HETATM	121	H	5.343	4.835	-3.892
HETATM	122	H	6.415	4.309	-5.200
HETATM	123	H	4.767	3.694	-5.112
HETATM	124	H	-2.451	-3.268	-4.787
HETATM	125	H	-3.297	-1.918	-5.607
HETATM	126	H	-3.992	-3.042	-2.842
HETATM	127	H	-4.913	-3.334	-4.337

```
HETATM  128  H              -4.817  -1.703  -3.629
HETATM  129  H               6.040   0.627   4.998
HETATM  130  H               4.660   1.082   6.003
HETATM  131  H               6.268   1.681   6.402
HETATM  132  H               7.329   2.450   3.689
HETATM  133  H               7.378   3.458   5.136
HETATM  134  H               6.702   4.112   3.644
HETATM  135  H               3.753   3.486   5.937
HETATM  136  H               4.508   4.691   4.887
HETATM  137  H               5.348   4.117   6.336
HETATM  138  H               1.820  -3.590   5.235
HETATM  139  H               2.889  -3.470   3.800
HETATM  140  H               0.255  -5.031   3.929
HETATM  141  H               1.882  -5.751   3.986
HETATM  142  H               1.306  -4.917   2.522
HETATM  143  H               0.190   2.496  -1.887
HETATM  144  H              -1.021   1.114  -0.107
HETATM  145  H              -0.177   2.603   2.250
HETATM  146  H               1.200   1.043   0.080
CONECT    1    2    3    4    5
CONECT    2    1
CONECT    3    1
CONECT    4    1    6
CONECT    5    1
CONECT    6    4
CONECT    7   44   49
CONECT    8   47   64
CONECT    9   44
CONECT   10   20   31   73
CONECT   11   16   17   75
CONECT   12   15   39   73
CONECT   13   17   27   76
CONECT   14   20   23   75
CONECT   15   12   18   29
CONECT   16   11   22   23
CONECT   17   11   13   21
CONECT   18   15   38   78
CONECT   19   26   30   78
CONECT   20   10   14   24
CONECT   21   17   25   79
CONECT   22   16   25   80
CONECT   23   14   16   32
CONECT   24   20   33   81
CONECT   25   21   22   56
```

```
CONECT   26   19   38   41
CONECT   27   13   35   42
CONECT   28   30   42   76
CONECT   29   15   36   82
CONECT   30   19   28   40
CONECT   31   10   39   47
CONECT   32   23   33   83
CONECT   33   24   32   45
CONECT   34   40   41   46
CONECT   35   27   74   77
CONECT   36   29   43   55
CONECT   37   47
CONECT   38   18   26   43
CONECT   39   12   31   44
CONECT   40   30   34   84
CONECT   41   26   34   85
CONECT   42   27   28   57
CONECT   43   36   38   86
CONECT   44    7    9   39
CONECT   45   33   51   52   54
CONECT   46   34   66   67   68
CONECT   47    8   31   37
CONECT   48   53   74   87   88
CONECT   49    7   50   89   90
CONECT   50   49   91   92   93
CONECT   51   45   94   95   96
CONECT   52   45   97   98   99
CONECT   53   48  100  101  102
CONECT   54   45  103  104  105
CONECT   55   36   61   62   63
CONECT   56   25   58   59   60
CONECT   57   42   69   72
CONECT   58   56  106  107  108
CONECT   59   56  109  110  111
CONECT   60   56  112  113  114
CONECT   61   55  115  116  117
CONECT   62   55  118  119  120
CONECT   63   55  121  122  123
CONECT   64    8   65  124  125
CONECT   65   64  126  127  128
CONECT   66   46  129  130  131
CONECT   67   46  132  133  134
CONECT   68   46  135  136  137
CONECT   69   57   70
```

```
CONECT   70   69   71  138  139
CONECT   71   70  140  141  142
CONECT   72   57
CONECT   73   10   12  143
CONECT   74   35   48
CONECT   75   11   14  144
CONECT   76   13   28  145
CONECT   77   35
CONECT   78   18   19  146
CONECT   79   21
CONECT   80   22
CONECT   81   24
CONECT   82   29
CONECT   83   32
CONECT   84   40
CONECT   85   41
CONECT   86   43
CONECT   87   48
CONECT   88   48
CONECT   89   49
CONECT   90   49
CONECT   91   50
CONECT   92   50
CONECT   93   50
CONECT   94   51
CONECT   95   51
CONECT   96   51
CONECT   97   52
CONECT   98   52
CONECT   99   52
CONECT  100   53
CONECT  101   53
CONECT  102   53
CONECT  103   54
CONECT  104   54
CONECT  105   54
CONECT  106   58
CONECT  107   58
CONECT  108   58
CONECT  109   59
CONECT  110   59
CONECT  111   59
CONECT  112   60
CONECT  113   60
```

```
CONECT  114   60
CONECT  115   61
CONECT  116   61
CONECT  117   61
CONECT  118   62
CONECT  119   62
CONECT  120   62
CONECT  121   63
CONECT  122   63
CONECT  123   63
CONECT  124   64
CONECT  125   64
CONECT  126   65
CONECT  127   65
CONECT  128   65
CONECT  129   66
CONECT  130   66
CONECT  131   66
CONECT  132   67
CONECT  133   67
CONECT  134   67
CONECT  135   68
CONECT  136   68
CONECT  137   68
CONECT  138   70
CONECT  139   70
CONECT  140   71
CONECT  141   71
CONECT  142   71
CONECT  143   73
CONECT  144   75
CONECT  145   76
CONECT  146   78
END
```

```
HEADER    MMFF94 OPTIMIZED HOST:HYDROGEN SULFATE COMPLEX
COMPND   LOWEST ENERGY CARBAZOLE-BOUND FORM BY SAMPLING IN VACUO MD RUN
AUTHOR    GENERATED BY PCMODEL V 9.30
HETATM    1  O       1.040  -4.129  -1.714
HETATM    2  S       0.414  -4.036  -0.244
HETATM    3  O       1.508  -4.571   0.548
HETATM    4  O      -0.775  -4.865  -0.325
HETATM    5  O       0.171  -2.604  -0.080
HETATM    6  H       1.945  -4.468  -1.546
HETATM    7  N      -0.053  -0.902  -2.233
HETATM    8  O      -4.381  -2.991  -2.409
HETATM    9  O      -4.041  -3.829   2.465
HETATM   10  O      -5.549  -3.514  -0.521
HETATM   11  C      -2.643  -0.976   1.364
HETATM   12  C       1.582  -0.538   2.600
HETATM   13  C      -2.859  -1.056  -0.902
HETATM   14  C       3.144  -1.123   0.729
HETATM   15  C      -0.678  -0.475   2.845
HETATM   16  C      -2.566  -0.600  -2.265
HETATM   17  C       1.312  -0.008   3.857
HETATM   18  C       2.882  -0.661   2.090
HETATM   19  C      -1.258  -0.500  -2.770
HETATM   20  C       1.006  -0.479  -3.010
HETATM   21  C      -2.065  -0.522   2.627
HETATM   22  C       3.934  -0.253   2.930
HETATM   23  C       2.398   0.386   4.674
HETATM   24  C      -0.113   0.030   4.013
HETATM   25  C      -2.882  -0.067   3.679
HETATM   26  C       3.727   0.261   4.225
HETATM   27  C       0.456   0.049  -4.173
HETATM   28  C       3.871  -2.192   0.212
HETATM   29  C       2.948  -1.037  -1.532
HETATM   30  C      -3.603  -0.142  -3.105
HETATM   31  C       2.391  -0.561  -2.793
HETATM   32  C      -3.655  -1.879   1.070
HETATM   33  C      -0.972   0.473   5.045
HETATM   34  C      -2.371   0.421   4.898
HETATM   35  C       2.726   0.390  -5.051
HETATM   36  C       4.582  -3.232   0.953
HETATM   37  C      -3.378   0.375  -4.392
HETATM   38  O      -5.452  -2.071   2.561
HETATM   39  C      -0.970   0.041  -4.019
```

HETATM	40	C	-3.760	-1.976	-0.367
HETATM	41	C	3.223	-0.125	-3.837
HETATM	42	C	1.328	0.483	-5.198
HETATM	43	C	3.772	-2.111	-1.226
HETATM	44	C	-2.041	0.471	-4.832
HETATM	45	C	-4.651	-2.890	-1.078
HETATM	46	C	-3.337	0.881	6.004
HETATM	47	C	3.705	0.830	-6.154
HETATM	48	C	-4.455	-2.589	2.063
HETATM	49	C	4.748	-4.433	2.966
HETATM	50	C	-5.166	-3.957	-3.109
HETATM	51	C	-4.729	-3.955	-4.561
HETATM	52	C	-4.195	2.056	5.488
HETATM	53	C	-4.264	-0.288	6.405
HETATM	54	C	4.108	-4.473	4.340
HETATM	55	C	-2.629	1.361	7.291
HETATM	56	C	-4.522	0.852	-5.304
HETATM	57	C	4.933	0.670	5.089
HETATM	58	C	4.328	-3.041	-2.194
HETATM	59	C	5.847	-0.553	5.317
HETATM	60	C	4.547	1.216	6.483
HETATM	61	C	5.735	1.778	4.373
HETATM	62	C	-5.933	0.586	-4.731
HETATM	63	C	-4.394	2.373	-5.529
HETATM	64	C	-4.448	0.130	-6.668
HETATM	65	C	-2.878	-4.442	1.878
HETATM	66	C	-2.630	-5.752	2.600
HETATM	67	C	3.011	1.354	-7.432
HETATM	68	C	4.606	1.967	-5.625
HETATM	69	C	4.588	-0.365	-6.574
HETATM	70	O	3.870	-4.311	-1.985
HETATM	71	C	4.420	-5.284	-2.876
HETATM	72	C	3.873	-6.643	-2.483
HETATM	73	O	5.089	-2.711	-3.096
HETATM	74	N	-2.192	-0.493	0.162
HETATM	75	O	4.209	-3.316	2.260
HETATM	76	N	0.370	-0.916	2.063
HETATM	77	N	2.603	-0.451	-0.341
HETATM	78	O	5.444	-3.941	0.442
HETATM	79	H	0.040	-1.465	-1.387
HETATM	80	H	4.948	-0.345	2.544
HETATM	81	H	2.164	0.788	5.653
HETATM	82	H	-3.961	-0.090	3.528
HETATM	83	H	-4.614	-0.198	-2.709

HETATM	84	H	-0.510	0.854	5.948
HETATM	85	H	4.300	-0.194	-3.685
HETATM	86	H	0.877	0.883	-6.100
HETATM	87	H	-1.807	0.889	-5.806
HETATM	88	H	5.833	-4.314	3.066
HETATM	89	H	4.527	-5.363	2.430
HETATM	90	H	-6.227	-3.692	-3.043
HETATM	91	H	-5.005	-4.951	-2.676
HETATM	92	H	-3.662	-4.189	-4.643
HETATM	93	H	-4.871	-2.964	-5.003
HETATM	94	H	-5.298	-4.686	-5.141
HETATM	95	H	-4.821	1.770	4.635
HETATM	96	H	-3.564	2.893	5.166
HETATM	97	H	-4.868	2.427	6.269
HETATM	98	H	-4.896	-0.622	5.575
HETATM	99	H	-4.936	0.003	7.221
HETATM	100	H	-3.683	-1.154	6.744
HETATM	101	H	4.300	-3.542	4.883
HETATM	102	H	4.493	-5.311	4.927
HETATM	103	H	3.020	-4.571	4.254
HETATM	104	H	-1.983	2.225	7.098
HETATM	105	H	-2.016	0.568	7.734
HETATM	106	H	-3.358	1.669	8.051
HETATM	107	H	5.300	-1.366	5.811
HETATM	108	H	6.703	-0.294	5.951
HETATM	109	H	6.252	-0.951	4.380
HETATM	110	H	3.998	0.470	7.070
HETATM	111	H	3.925	2.115	6.406
HETATM	112	H	5.438	1.489	7.061
HETATM	113	H	6.150	1.439	3.418
HETATM	114	H	6.579	2.115	4.986
HETATM	115	H	5.104	2.651	4.166
HETATM	116	H	-6.712	0.917	-5.429
HETATM	117	H	-6.097	1.126	-3.792
HETATM	118	H	-6.097	-0.482	-4.545
HETATM	119	H	-4.425	2.917	-4.577
HETATM	120	H	-5.210	2.751	-6.156
HETATM	121	H	-3.455	2.638	-6.028
HETATM	122	H	-4.489	-0.958	-6.542
HETATM	123	H	-3.528	0.364	-7.215
HETATM	124	H	-5.284	0.422	-7.316
HETATM	125	H	-3.078	-4.642	0.822
HETATM	126	H	-2.007	-3.792	1.998
HETATM	127	H	-2.467	-5.577	3.670

```
HETATM  128  H              -3.500  -6.411   2.516
HETATM  129  H              -1.756  -6.266   2.191
HETATM  130  H               2.394   2.236  -7.224
HETATM  131  H               3.748   1.649  -8.189
HETATM  132  H               2.371   0.589  -7.886
HETATM  133  H               4.006   2.822  -5.290
HETATM  134  H               5.224   1.647  -4.779
HETATM  135  H               5.290   2.324  -6.404
HETATM  136  H               3.975  -1.205  -6.923
HETATM  137  H               5.268  -0.088  -7.387
HETATM  138  H               5.209  -0.733  -5.750
HETATM  139  H               4.126  -5.050  -3.905
HETATM  140  H               5.512  -5.289  -2.795
HETATM  141  H               4.132  -6.877  -1.446
HETATM  142  H               2.780  -6.652  -2.548
HETATM  143  H               4.271  -7.427  -3.134
HETATM  144  H              -1.467   0.203   0.068
HETATM  145  H               0.268  -1.470   1.212
HETATM  146  H               1.995   0.352  -0.257
CONECT    1    2    6
CONECT    2    1    3    4    5
CONECT    3    2
CONECT    4    2
CONECT    5    2
CONECT    6    1
CONECT    7   19   20   79
CONECT    8   45   50
CONECT    9   48   65
CONECT   10   45
CONECT   11   21   32   74
CONECT   12   17   18   76
CONECT   13   16   40   74
CONECT   14   18   28   77
CONECT   15   21   24   76
CONECT   16   13   19   30
CONECT   17   12   23   24
CONECT   18   12   14   22
CONECT   19    7   16   39
CONECT   20    7   27   31
CONECT   21   11   15   25
CONECT   22   18   26   80
CONECT   23   17   26   81
CONECT   24   15   17   33
CONECT   25   21   34   82
```

```
CONECT   26   22   23   57
CONECT   27   20   39   42
CONECT   28   14   36   43
CONECT   29   31   43   77
CONECT   30   16   37   83
CONECT   31   20   29   41
CONECT   32   11   40   48
CONECT   33   24   34   84
CONECT   34   25   33   46
CONECT   35   41   42   47
CONECT   36   28   75   78
CONECT   37   30   44   56
CONECT   38   48
CONECT   39   19   27   44
CONECT   40   13   32   45
CONECT   41   31   35   85
CONECT   42   27   35   86
CONECT   43   28   29   58
CONECT   44   37   39   87
CONECT   45    8   10   40
CONECT   46   34   52   53   55
CONECT   47   35   67   68   69
CONECT   48    9   32   38
CONECT   49   54   75   88   89
CONECT   50    8   51   90   91
CONECT   51   50   92   93   94
CONECT   52   46   95   96   97
CONECT   53   46   98   99  100
CONECT   54   49  101  102  103
CONECT   55   46  104  105  106
CONECT   56   37   62   63   64
CONECT   57   26   59   60   61
CONECT   58   43   70   73
CONECT   59   57  107  108  109
CONECT   60   57  110  111  112
CONECT   61   57  113  114  115
CONECT   62   56  116  117  118
CONECT   63   56  119  120  121
CONECT   64   56  122  123  124
CONECT   65    9   66  125  126
CONECT   66   65  127  128  129
CONECT   67   47  130  131  132
CONECT   68   47  133  134  135
CONECT   69   47  136  137  138
```

```
CONECT   70   58   71
CONECT   71   70   72  139  140
CONECT   72   71  141  142  143
CONECT   73   58
CONECT   74   11   13  144
CONECT   75   36   49
CONECT   76   12   15  145
CONECT   77   14   29  146
CONECT   78   36
CONECT   79    7
CONECT   80   22
CONECT   81   23
CONECT   82   25
CONECT   83   30
CONECT   84   33
CONECT   85   41
CONECT   86   42
CONECT   87   44
CONECT   88   49
CONECT   89   49
CONECT   90   50
CONECT   91   50
CONECT   92   51
CONECT   93   51
CONECT   94   51
CONECT   95   52
CONECT   96   52
CONECT   97   52
CONECT   98   53
CONECT   99   53
CONECT  100   53
CONECT  101   54
CONECT  102   54
CONECT  103   54
CONECT  104   55
CONECT  105   55
CONECT  106   55
CONECT  107   59
CONECT  108   59
CONECT  109   59
CONECT  110   60
CONECT  111   60
CONECT  112   60
CONECT  113   61
```

```
CONECT  114   61
CONECT  115   61
CONECT  116   62
CONECT  117   62
CONECT  118   62
CONECT  119   63
CONECT  120   63
CONECT  121   63
CONECT  122   64
CONECT  123   64
CONECT  124   64
CONECT  125   65
CONECT  126   65
CONECT  127   66
CONECT  128   66
CONECT  129   66
CONECT  130   67
CONECT  131   67
CONECT  132   67
CONECT  133   68
CONECT  134   68
CONECT  135   68
CONECT  136   69
CONECT  137   69
CONECT  138   69
CONECT  139   71
CONECT  140   71
CONECT  141   72
CONECT  142   72
CONECT  143   72
CONECT  144   74
CONECT  145   76
CONECT  146   77
END
```

HEADER MMFF94 OPTIMIZED CALIX[4]PYRROLE:CHLORIDE COMPLEX

COMPND LOWEST ENERGY COMPLEX BY SAMPLING IN VACUO MD RUN

AUTHOR GENERATED BY PCMODEL V 9.30

HETATM	1	Cl	0.000	0.000	2.529
HETATM	2	N	2.391	0.000	0.187
HETATM	4	N	0.000	2.437	0.170
HETATM	6	N	-2.391	0.000	0.188
HETATM	8	N	0.000	-2.437	0.170
HETATM	10	C	2.758	-1.139	-0.511
HETATM	11	C	3.269	-0.705	-1.721
HETATM	13	C	3.269	0.704	-1.721
HETATM	15	C	2.758	1.139	-0.511
HETATM	16	C	3.605	3.497	-0.617
HETATM	20	C	2.541	2.556	0.015
HETATM	21	C	2.787	2.648	1.538
HETATM	25	C	1.139	3.045	-0.329
HETATM	26	C	0.705	4.080	-1.138
HETATM	28	C	-0.706	4.080	-1.138
HETATM	30	C	-1.139	3.045	-0.329
HETATM	31	C	-3.605	3.498	-0.615
HETATM	35	C	-2.540	2.556	0.016
HETATM	36	C	-2.786	2.648	1.539
HETATM	40	C	-2.758	1.139	-0.510
HETATM	41	C	-3.270	0.705	-1.720
HETATM	43	C	-3.270	-0.705	-1.719
HETATM	45	C	-2.758	-1.139	-0.510
HETATM	46	C	-3.605	-3.497	-0.615
HETATM	50	C	-2.540	-2.556	0.016
HETATM	51	C	-2.786	-2.648	1.539
HETATM	55	C	-1.139	-3.045	-0.329
HETATM	56	C	-0.706	-4.080	-1.138
HETATM	58	C	0.705	-4.080	-1.138
HETATM	60	C	1.139	-3.045	-0.329
HETATM	61	C	3.605	-3.498	-0.617
HETATM	65	C	2.541	-2.556	0.015
HETATM	66	C	2.787	-2.648	1.538
HETATM	3	H	1.885	0.000	1.074
HETATM	5	H	0.000	1.640	0.809
HETATM	7	H	-1.884	0.000	1.075
HETATM	9	H	0.000	-1.640	0.809
HETATM	12	H	3.603	-1.340	-2.529
HETATM	14	H	3.603	1.340	-2.529

```
HETATM   17  H              4.623   3.150  -0.400
HETATM   18  H              3.516   4.520  -0.230
HETATM   19  H              3.509   3.559  -1.707
HETATM   22  H              2.072   2.064   2.118
HETATM   23  H              2.700   3.683   1.891
HETATM   24  H              3.788   2.288   1.800
HETATM   27  H              1.339   4.759  -1.691
HETATM   29  H             -1.340   4.759  -1.690
HETATM   32  H             -3.516   4.520  -0.228
HETATM   33  H             -4.623   3.150  -0.396
HETATM   34  H             -3.510   3.559  -1.706
HETATM   37  H             -2.071   2.064   2.119
HETATM   38  H             -3.787   2.288   1.803
HETATM   39  H             -2.698   3.683   1.892
HETATM   42  H             -3.604   1.340  -2.527
HETATM   44  H             -3.604  -1.340  -2.527
HETATM   47  H             -4.623  -3.150  -0.396
HETATM   48  H             -3.516  -4.520  -0.228
HETATM   49  H             -3.510  -3.559  -1.706
HETATM   52  H             -2.071  -2.064   2.119
HETATM   53  H             -2.698  -3.683   1.892
HETATM   54  H             -3.787  -2.288   1.803
HETATM   57  H             -1.340  -4.759  -1.690
HETATM   59  H              1.339  -4.759  -1.691
HETATM   62  H              3.516  -4.520  -0.230
HETATM   63  H              4.623  -3.150  -0.400
HETATM   64  H              3.509  -3.559  -1.707
HETATM   67  H              2.072  -2.064   2.118
HETATM   68  H              3.788  -2.288   1.800
HETATM   69  H              2.700  -3.683   1.891
CONECT    2   10   15    3
CONECT    4   25   30    5
CONECT    6   40   45    7
CONECT    8   55   60    9
CONECT   10    2   11   65
CONECT   11   10   13   12
CONECT   13   11   15   14
CONECT   15    2   13   20
CONECT   16   20   17   18   19
CONECT   20   15   16   21   25
CONECT   21   20   22   23   24
CONECT   25    4   20   26
CONECT   26   25   28   27
CONECT   28   26   30   29
```

```
CONECT   30    4   28   35
CONECT   31   35   32   33   34
CONECT   35   30   31   36   40
CONECT   36   35   37   38   39
CONECT   40    6   35   41
CONECT   41   40   43   42
CONECT   43   41   45   44
CONECT   45    6   43   50
CONECT   46   50   47   48   49
CONECT   50   45   46   51   55
CONECT   51   50   52   53   54
CONECT   55    8   50   56
CONECT   56   55   58   57
CONECT   58   56   60   59
CONECT   60    8   58   65
CONECT   61   65   62   63   64
CONECT   65   10   60   61   66
CONECT   66   65   67   68   69
CONECT    3    2
CONECT    5    4
CONECT    7    6
CONECT    9    8
CONECT   12   11
CONECT   14   13
CONECT   17   16
CONECT   18   16
CONECT   19   16
CONECT   22   21
CONECT   23   21
CONECT   24   21
CONECT   27   26
CONECT   29   28
CONECT   32   31
CONECT   33   31
CONECT   34   31
CONECT   37   36
CONECT   38   36
CONECT   39   36
CONECT   42   41
CONECT   44   43
CONECT   47   46
CONECT   48   46
CONECT   49   46
CONECT   52   51
```

```
CONECT   53   51
CONECT   54   51
CONECT   57   56
CONECT   59   58
CONECT   62   61
CONECT   63   61
CONECT   64   61
CONECT   67   66
CONECT   68   66
CONECT   69   66
END
```

```
HEADER    MMFF94 OPTIMIZED CALIX[4]PYRROLE HOST
COMPND   LOWEST ENERGY 1,3-ALTERNATE CONFORMER BY SAMPLING IN VACUO MD RUN
AUTHOR    GENERATED BY PCMODEL V 9.30
HETATM    1  C     -1.135   2.679  -0.605
HETATM    2  C     -0.707   3.104  -1.849
HETATM    3  C      0.707   3.104  -1.849
HETATM    4  C      1.135   2.679  -0.605
HETATM    5  C      2.520   2.520   0.000
HETATM    6  C      2.679   1.135   0.605
HETATM    7  C      3.105   0.707   1.849
HETATM    8  C      3.105  -0.707   1.849
HETATM    9  C      2.679  -1.135   0.605
HETATM   10  C      2.520  -2.520   0.000
HETATM   11  C      1.135  -2.679  -0.605
HETATM   12  C      0.707  -3.104  -1.850
HETATM   13  C     -0.707  -3.104  -1.850
HETATM   14  C     -1.135  -2.679  -0.605
HETATM   15  C     -2.520  -2.520   0.000
HETATM   16  C     -2.679  -1.135   0.605
HETATM   17  C     -3.104  -0.707   1.850
HETATM   18  C     -3.104   0.707   1.850
HETATM   19  C     -2.679   1.135   0.605
HETATM   20  C     -2.520   2.520   0.000
HETATM   21  C      2.716   3.622   1.067
HETATM   22  C      2.716  -3.622   1.066
HETATM   23  C      3.622  -2.716  -1.067
HETATM   24  C     -2.716  -3.622   1.067
HETATM   25  C     -2.716   3.622   1.067
HETATM   26  C     -3.622   2.716  -1.067
HETATM   27  C      3.621   2.716  -1.067
HETATM   28  C     -3.622  -2.716  -1.067
HETATM   29  N      0.000   2.397   0.125
HETATM   30  N      2.397   0.000  -0.125
HETATM   31  N      0.000  -2.397   0.124
HETATM   32  N     -2.397   0.000  -0.124
HETATM   37  H     -1.343   3.407  -2.672
HETATM   38  H      1.343   3.407  -2.672
HETATM   39  H      3.407   1.343   2.672
HETATM   40  H      3.407  -1.343   2.671
HETATM   41  H      1.343  -3.407  -2.672
HETATM   42  H     -1.343  -3.407  -2.672
HETATM   43  H     -3.406  -1.343   2.672
```

```
HETATM  44  H         -3.406   1.343   2.672
HETATM  45  H          2.000   3.521   1.892
HETATM  46  H          3.723   3.588   1.500
HETATM  47  H          2.576   4.621   0.636
HETATM  48  H          2.000  -3.521   1.892
HETATM  49  H          2.576  -4.621   0.636
HETATM  50  H          3.723  -3.588   1.500
HETATM  51  H          3.521  -2.000  -1.892
HETATM  52  H          4.621  -2.576  -0.637
HETATM  53  H          3.588  -3.722  -1.500
HETATM  54  H         -2.000  -3.522   1.892
HETATM  55  H         -3.722  -3.588   1.500
HETATM  56  H         -2.575  -4.621   0.636
HETATM  57  H         -2.000   3.521   1.892
HETATM  58  H         -2.576   4.621   0.636
HETATM  59  H         -3.722   3.588   1.500
HETATM  60  H         -3.522   2.000  -1.892
HETATM  61  H         -4.621   2.576  -0.636
HETATM  62  H         -3.588   3.723  -1.500
HETATM  63  H          3.521   2.000  -1.892
HETATM  64  H          3.588   3.723  -1.500
HETATM  65  H          4.621   2.576  -0.636
HETATM  66  H         -3.521  -2.000  -1.892
HETATM  67  H         -3.588  -3.723  -1.500
HETATM  68  H         -4.621  -2.576  -0.636
HETATM  33  H          0.000   2.035   1.071
HETATM  34  H          2.035   0.000  -1.071
HETATM  35  H          0.000  -2.035   1.071
HETATM  36  H         -2.035   0.000  -1.071
CONECT   1   2  20  29
CONECT   2   1   3  37
CONECT   3   2   4  38
CONECT   4   3   5  29
CONECT   5   4   6  21  27
CONECT   6   5   7  30
CONECT   7   6   8  39
CONECT   8   7   9  40
CONECT   9   8  10  30
CONECT  10   9  11  22  23
CONECT  11  10  12  31
CONECT  12  11  13  41
CONECT  13  12  14  42
CONECT  14  13  15  31
CONECT  15  14  16  24  28
```

```
CONECT  16  15  17  32
CONECT  17  16  18  43
CONECT  18  17  19  44
CONECT  19  18  20  32
CONECT  20   1  19  25  26
CONECT  21   5  45  46  47
CONECT  22  10  48  49  50
CONECT  23  10  51  52  53
CONECT  24  15  54  55  56
CONECT  25  20  57  58  59
CONECT  26  20  60  61  62
CONECT  27   5  63  64  65
CONECT  28  15  66  67  68
CONECT  29   1   4  33
CONECT  30   6   9  34
CONECT  31  11  14  35
CONECT  32  16  19  36
CONECT  37   2
CONECT  38   3
CONECT  39   7
CONECT  40   8
CONECT  41  12
CONECT  42  13
CONECT  43  17
CONECT  44  18
CONECT  45  21
CONECT  46  21
CONECT  47  21
CONECT  48  22
CONECT  49  22
CONECT  50  22
CONECT  51  23
CONECT  52  23
CONECT  53  23
CONECT  54  24
CONECT  55  24
CONECT  56  24
CONECT  57  25
CONECT  58  25
CONECT  59  25
CONECT  60  26
CONECT  61  26
CONECT  62  26
CONECT  63  27
```

```
CONECT   64   27
CONECT   65   27
CONECT   66   28
CONECT   67   28
CONECT   68   28
CONECT   33   29
CONECT   34   30
CONECT   35   31
CONECT   36   32
END
```

HEADER   MMFF94 OPTIMIZED CALIX[4]PYRROLE HOST

COMPND   1,2-ALTERNATE CONFORMER

AUTHOR   GENERATED BY PCMODEL V 9.30

HETATM	1	N	0.138	2.412	-0.037
HETATM	2	N	-2.508	-0.174	0.283
HETATM	3	C	1.218	2.973	-0.686
HETATM	4	C	0.700	3.777	-1.684
HETATM	5	C	-0.710	3.688	-1.628
HETATM	6	C	-1.049	2.839	-0.589
HETATM	7	C	-2.397	2.360	-0.070
HETATM	8	C	-2.630	0.928	-0.535
HETATM	9	C	-2.961	0.446	-1.790
HETATM	10	C	-3.055	-0.961	-1.715
HETATM	11	C	-2.759	-1.337	-0.418
HETATM	12	C	2.649	2.705	-0.245
HETATM	13	C	-2.446	2.486	1.473
HETATM	14	C	-3.541	3.259	-0.602
HETATM	15	C	3.070	3.826	0.731
HETATM	16	C	3.613	2.756	-1.453
HETATM	35	C	-2.649	-2.705	0.245
HETATM	36	C	-1.218	-2.973	0.686
HETATM	37	N	-0.138	-2.412	0.037
HETATM	38	C	1.049	-2.839	0.589
HETATM	39	C	0.710	-3.688	1.628
HETATM	40	C	-0.700	-3.777	1.684
HETATM	43	C	2.397	-2.360	0.070
HETATM	44	C	2.630	-0.928	0.535
HETATM	45	N	2.508	0.174	-0.283
HETATM	46	C	2.759	1.337	0.418
HETATM	47	C	3.055	0.961	1.714
HETATM	48	C	2.961	-0.446	1.790
HETATM	52	C	2.446	-2.486	-1.473
HETATM	56	C	3.541	-3.259	0.602
HETATM	61	C	-3.070	-3.826	-0.731
HETATM	65	C	-3.613	-2.756	1.453
HETATM	17	H	0.219	1.762	0.736
HETATM	18	H	-2.242	-0.155	1.259
HETATM	19	H	1.275	4.375	-2.379
HETATM	20	H	-1.405	4.197	-2.283
HETATM	21	H	-3.115	1.052	-2.674
HETATM	22	H	-3.296	-1.628	-2.533
HETATM	23	H	-1.676	1.886	1.971

```
HETATM  24  H          -2.292   3.525   1.789
HETATM  25  H          -3.416   2.159   1.868
HETATM  26  H          -3.411   4.300  -0.284
HETATM  27  H          -4.516   2.917  -0.234
HETATM  28  H          -3.595   3.261  -1.697
HETATM  29  H           2.420   3.857   1.615
HETATM  30  H           3.011   4.812   0.255
HETATM  31  H           4.100   3.689   1.081
HETATM  32  H           3.317   2.053  -2.241
HETATM  33  H           4.637   2.502  -1.153
HETATM  34  H           3.643   3.754  -1.905
HETATM  60  H          -0.219  -1.762  -0.736
HETATM  42  H           1.405  -4.197   2.283
HETATM  41  H          -1.275  -4.375   2.379
HETATM  51  H           2.242   0.155  -1.259
HETATM  50  H           3.296   1.627   2.533
HETATM  49  H           3.115  -1.052   2.674
HETATM  53  H           1.676  -1.886  -1.971
HETATM  54  H           2.292  -3.525  -1.789
HETATM  55  H           3.416  -2.159  -1.868
HETATM  57  H           3.411  -4.300   0.284
HETATM  58  H           4.516  -2.917   0.234
HETATM  59  H           3.595  -3.261   1.697
HETATM  62  H          -2.420  -3.857  -1.615
HETATM  63  H          -3.011  -4.812  -0.255
HETATM  64  H          -4.100  -3.689  -1.081
HETATM  66  H          -3.317  -2.053   2.241
HETATM  67  H          -4.637  -2.502   1.153
HETATM  68  H          -3.643  -3.754   1.905
CONECT    1    3    6   17
CONECT    2    8   11   18
CONECT    3    1    4   12
CONECT    4    3    5   19
CONECT    5    4    6   20
CONECT    6    1    5    7
CONECT    7    6    8   13   14
CONECT    8    2    7    9
CONECT    9    8   10   21
CONECT   10    9   11   22
CONECT   11    2   10   35
CONECT   12    3   15   16   46
CONECT   13    7   23   24   25
CONECT   14    7   26   27   28
CONECT   15   12   29   30   31
```

```
CONECT   16   12   32   33   34
CONECT   35   11   36   61   65
CONECT   36   35   37   40
CONECT   37   36   38   60
CONECT   38   37   39   43
CONECT   39   38   40   42
CONECT   40   36   39   41
CONECT   43   38   44   52   56
CONECT   44   43   45   48
CONECT   45   44   46   51
CONECT   46   12   45   47
CONECT   47   46   48   50
CONECT   48   44   47   49
CONECT   52   43   53   54   55
CONECT   56   43   57   58   59
CONECT   61   35   62   63   64
CONECT   65   35   66   67   68
CONECT   17    1
CONECT   18    2
CONECT   19    4
CONECT   20    5
CONECT   21    9
CONECT   22   10
CONECT   23   13
CONECT   24   13
CONECT   25   13
CONECT   26   14
CONECT   27   14
CONECT   28   14
CONECT   29   15
CONECT   30   15
CONECT   31   15
CONECT   32   16
CONECT   33   16
CONECT   34   16
CONECT   60   37
CONECT   42   39
CONECT   41   40
CONECT   51   45
CONECT   50   47
CONECT   49   48
CONECT   53   52
CONECT   54   52
CONECT   55   52
```

```
CONECT   57   56
CONECT   58   56
CONECT   59   56
CONECT   62   61
CONECT   63   61
CONECT   64   61
CONECT   66   65
CONECT   67   65
CONECT   68   65
END
```
